# Small Intestinal Tuft Cell Activity Associates With Energy Metabolism in Diet-Induced Obesity

**DOI:** 10.3389/fimmu.2021.629391

**Published:** 2021-05-28

**Authors:** Pankaj Arora, Daniel Andersen, Janne Marie Moll, Niels Banhos Danneskiold-Samsøe, Liqin Xu, Biaofeng Zhou, Georgios Kladis, Philipp Rausch, Christopher T. Workman, Karsten Kristiansen, Susanne Brix

**Affiliations:** ^1^ Department of Biotechnology and Biomedicine, Technical University of Denmark, Kongens Lyngby, Denmark; ^2^ Laboratory of Genomics and Molecular Biomedicine, Department of Biology, University of Copenhagen, Copenhagen, Denmark; ^3^ BGI-Shenzhen, Shenzhen, China

**Keywords:** tuft cells, high fat diet, gut-brain axis, GABA, neuroserpin, metabolism, type 2 immune responses

## Abstract

Little is known about the involvement of type 2 immune response-promoting intestinal tuft cells in metabolic regulation. We here examined the temporal changes in small intestinal tuft cell number and activity in response to high-fat diet-induced obesity in mice and investigated the relation to whole-body energy metabolism and the immune phenotype of the small intestine and epididymal white adipose tissue. Intake of high fat diet resulted in a reduction in overall numbers of small intestinal epithelial and tuft cells and reduced expression of the intestinal type 2 tuft cell markers *Il25* and *Tslp*. Amongst >1,700 diet-regulated transcripts in tuft cells, we observed an early association between body mass expansion and increased expression of the gene encoding the serine protease inhibitor neuroserpin. By contrast, tuft cell expression of genes encoding gamma aminobutyric acid (GABA)-receptors was coupled to *Tslp* and *Il25* and reduced body mass gain. Combined, our results point to a possible role for small intestinal tuft cells in energy metabolism *via* coupled regulation of tuft cell type 2 markers and GABA signaling receptors, while being independent of type 2 immune cell involvement. These results pave the way for further studies into interventions that elicit anti-obesogenic circuits *via* small intestinal tuft cells.

## Introduction

Intestinal tuft cells constitute a rare epithelial cell type located close to metabolism-regulating enteroendocrine cells and enteric nerve fibers in the gut (1). Therefore, tuft cells have been suggested to be involved in transmitting gut-brain axis signals and in regulating satiety and energy metabolism, but little is known about their actual involvement. Today, small intestinal tuft cells are acknowledged as key players in the initiation of potent type 2 immune responses against helminth infections in mice (2–4) *via* specific interaction with lamina propria type 2 innate lymphoid cells (ILC2s), with whom they form a tuft cell-ILC2 circuit *via* paracrine cytokine interactions. This tuft-ILC2 circuit has been shown to regulate adaptive intestinal remodeling in response to dietary cues (5). Thus, a constitutively activated tuft-ILC2 circuit results in increased small intestinal length with an increased ratio of secretory (tuft and goblet cells) to absorptive (enterocytes) cells, which severely reduces absorptive capacity. Additionally, mice fasted for 48 hours had increased levels of tuft cells that remained elevated after refeeding (6), altogether suggesting that tuft cells may adapt to dietary conditions.

Diet-induced obesity in mice and men is characterized by a chronic low-grade systemic inflammation coupled with alterations in the gut microbiota, increased levels of pro-inflammatory cytokines as well as altered immune cell profiles in visceral adipose tissue (7). A few studies have also shown that high-fat diet (HFD) feeding increased frequencies of Th1 and Th17 cells along with a decreased number of regulatory T cells (Tregs) and eosinophils in the small intestinal lamina propria (8–10). HFD feeding also results in considerable changes in cellular functions within the intestine, including higher intestinal stem cell numbers and self-renewal capacity (11), lower goblet cell differentiation and higher levels of misfolded Muc2 due to oxidative stress (12), lower number of small intestinal Paneth cells and antimicrobial peptides (13, 14), lower number of enteroendocrine cells with altered gut hormone production (15), as well as reduced enterocyte proliferation with altered metabolic activity (16, 17).

We here aimed to characterize functional and temporal aspects of intestinal tuft cells in response to HFD feeding focusing on their role in relaying immune-metabolic signals. To this end, we examined how HFD-associated effects on tuft cells coupled with small intestinal and epididymal white adipose tissue (eWAT) immune profiles as well as whole-body metabolic parameters. Our results suggest novel mechanisms whereby tuft cells may be involved in modulating whole-body energy metabolism.

## Materials and Methods

### Experimental Design

Male C57BL/6J (Taconic Biosciences A/S, Lille Skensved, Denmark) mice aged 5 weeks at arrival (week 0) were housed (4 mice per cage) in a climate-controlled room (temperature: 30°C and humidity: 55 ± 5%) subjected to a 12 h light/dark cycle (6:00 a.m. to 6:00 p.m.) with ad libitum access to water and food. During 1 week of acclimatization (week 0 to week 1), all mice were fed a normal chow diet (Altromin 1324, Brogaarden, Denmark). After acclimatization, the mice were distributed into 4 treatment groups with 12 mice in each group and offered one of two irradiated experimental diets: a synthetic low-fat reference diet (RFD) (EF D12450J) or a high-fat diet (HFD) (EF D12492) (ssniff, Germany, composition in **Table S1**). Mice were assigned into the four treatment groups based on MRI scans at arrival and distributed into cages based on their body mass, resulting in similar sums of body masses in each cage at study initiation. The RFD contained 10% fat (4.4% from lard and 5.6% from soybean oil). The HFD contained 60% fat (54.4% from lard and 5.6% from soybean oil). The mice were fed the two diets for either 9 or 22 weeks. All animals were handled similarly during standard procedures including weekly cage changes and body weight measurements, and general cleaning procedures at the animal facility. At the end of each experimental period, mice were terminated on three consecutive days, with one cage from each group each day, and randomized between treatment groups. Liver, eWAT, iWAT, BAT, small intestine and cecum were harvested at termination. Liver, cecum, eWAT, iWAT and BAT were weighed, and single-cell suspension for flow cytometry analysis were prepared from the small intestinal epithelium for sorting of tuft cells, and from the small intestinal lamina propria and parts of eWAT for immune cell quantification. Other parts of the eWAT and ileum were used for protein analyses. The samples collected at study termination were randomly distributed before analysis of the biomaterials described below.

### MRI Scan, Glucose, and Insulin Tolerance Tests

To measure lean and fat mass development during the experiments, MRI was performed on mice at specific time points using EchoMRI as per the manufacturer’s instructions. Oral glucose tolerance test (OGTT) and insulin tolerance test (ITT) were conducted in the 22 week groups at week 20 (OGTT) and week 21 (ITT). Prior to ITT and OGTT, co-housed mice were transferred to clean cages, and provided with bedding and free access to water. For OGTT, mice were feed-deprived for 5 hours (OGTT) and then gavaged with a 45% glucose solution (Sigma-Aldrich, G8769) corresponding to 3 g/kg of lean mass. For ITT, mice were feed-deprived for 2 hours, as recommended (18), and injected intraperitoneally with 0.75 U human insulin (Atrapid, Novo Nordisk, Denmark) per kg lean mass. Blood glucose levels were measured at 0, 15, 30, 45, 60, 90 and 120 min with a glucometer and glucose strips (Contour, Bayer) on samples collected from the tail vein tip.

### Single-Cell Preparation

Single-cell suspensions were prepared from 20 cm small intestine and epididymal (eWAT) white adipose tissue. Single-cell epithelial as well as lamina propria (LP) preparations were prepared from the small intestine in two steps. In all single-cell preparations, DMEM (Faculty of Health and Medical Sciences, University of Copenhagen, 1865), PBS (Mg^2+^ and Ca^2+^-free, pH 7.4, Gibco, 14190), fetal bovine serum (FBS, heat-inactivated, Gibco, 10270) and FACS buffer (3 mM EDTA in PBS + 5% FBS) were used. Harvested small intestines (1 cm distal to stomach) were flushed with ice-cold PBS, made devoid of visible mesenteric fat and Peyer’s patches, cut opened and washed manually with PBS to remove remaining intestinal contents. Intestinal tissue was cut into 2 cm pieces and incubated with rocking (900 rpm) and occasional flicking at 37°C for 20 min in 2*1.5 mL 30 mM EDTA (Sigma, ED2SS) in PBS. Obtained epithelial cell suspensions were washed (500g, 5 min at 5°C) in 3 mL PBS and incubated with rocking (300 rpm) and occasional flicking at 37°C for 10 min in 2*1.5 mL PBS supplemented with 20 μg/mL Dispase DH (Sigma-Aldrich, D4818) and 20 μg/mL DNase I (Sigma-Aldrich, 11284932001). Afterwards, epithelial cell suspensions were strained through 70-µm nylon filters while homogenizing the particulate matter, washed in 15 mL DMEM supplemented with 5% FBS, and suspended in FACS buffer.

For lamina propria single-cell preparation, the left-over intestinal tissues after EDTA treatment were rinsed with 10 mL PBS containing 5% FBS, minced into 1–2 mm pieces with scissors, incubated with rocking (500 rpm) and occasional flicking at 37°C for 30 min in 2*1.5 mL LP digestion buffer (DMEM supplemented with 5% FBS, 40 μg/mL Liberase DH (Sigma-Aldrich, 05401054001) and 50 μg/mL DNase I). Afterwards, 1 mL supernatant was transferred to a new vial, while the remaining cell suspension was repeatedly drawn through an 18-gauge needle to further mince the intestinal pieces. Fresh 1 mL LP digestion buffer was added, and the cell suspension was incubated again as described above. Afterwards, the LP cell suspension was passed through a 70-µm nylon filter while homogenizing the particulate matter, washed in 20 mL DMEM containing 5% FBS and suspended in FACS buffer.

For eWAT single-cell preparation, the tissues were harvested, finely chopped with scalpel blades and incubated at 37°C for 40 min in 1 mL DMEM containing 10% FBS and 20 mg/mL collagenase (Sigma-Aldrich, C6885) in a shaking incubator (150 rpm). 14 mL PBS containing 1% FBS was added to dilute the digestion reaction and afterwards digesta was strained through 70-µm nylon filters, washed (400g, 10 min at 5°C) in PBS, and subjected to red blood cell lysis using in-house made 1 mL lysis buffer (ACK buffer, 154.95 mM ammonium chloride, 9.99 mM sodium hydrogen carbonate, 0.0995 mM disodium EDTA, in PBS) for 5 minutes at room temperature. Finally, the cell suspensions were washed again in 14 mL PBS and suspended in FACS buffer. All single-cell suspensions were counted on a Nucleocounter (NC-200, ChemoMetec, Denmark) and plated for staining with antibodies before flow cytometric analysis.

### Staining of Cells for Flow Cytometry and Cell Sorting

All cell suspensions were incubated with Fc-block (anti-CD16/CD32, BD Biosciences) followed by staining with antibodies against surface markers. Cells were kept at 4°C throughout the staining procedure. Single-cell epithelial preparations were stained [CD45-eVolve 605 (eBiosciences, 30-F11), EpCAM-AF647(Biolegend, G8.8) and Siglec-F-PE (BD Biosciences, E50-2440)] for 30 min at 4°C and washed in FACS buffer followed by 0.3 µM 4’, 6-diamidine-20-phenylindole dihydrochloride (DAPI, 1.2 µL/10e6 cells) addition for dead cell exclusion. Live EpCAM+CD45+ Siglec-F+ tuft and EpCAM+CD45+ Siglec-F- non-tuft cell populations were sorted using Aria III (BD Biosciences) with a four laser (405nm, 488nm, 562nm and 633nm) configuration directly into RNA lysis buffer from a Quick-RNA Microprep Kit (Zymogen, R1050, R1051) and were stored at -20°C immediately after enrichment of tuft cells and at -80°C afterwards. Flow cytometric counting beads (CountBright Absolute; Life Technologies, C36950) were used to enumerate total live cell numbers. Intestinal tuft cell analysis and sorting were performed using the epithelial cell adhesion molecule (EPCAM) and sialic acid binding Ig-like lectin F (SIGLEC-F) as positive markers for identification of tuft cells. Siglec-F is also expressed on intestinal M-cells (19), and we therefore used CD45 expression to exclude cells co-expressing CD45 to leave out M-cells. To confirm that the analyzed and sorted cells were tuft cells, we used the common tuft and M-cell specific markers doublecortin-like kinase 1 (Dclk1) and Il25 and the M-cell specific marker glycoprotein 2 (Gp2), and performed RT-qPCR on RNA extracted from sorted cells.

Respective LP and eWAT single-cell preparations were divided into 2 fractions and were first stained with fixable violet live/dead stain (Life Technologies, L34955) as per manufacturer’s instructions and then incubated with Fc-block as described above. Afterwards, one fraction was stained for surface markers with the following antibody cocktails in FACS buffer (30 min at 4°C) supplemented with 2 µM monensin (Sigma-Aldrich, M5273) for intercellular cytokine detection and other fraction without monensin. The monensin supplemented antibody cocktail included CD45-PerCp (Biolegend, clone: 30-F11), CD11c-BV650 (Biolegend, N418), CD11b-AF 700 (eBiosciences, M1/70), F4/80-PE-Cy7 (Biolegend, BM8), CD206-BV605 (Biolegend, C068C2), Siglec-F-PE (BD, E50-2440), FceR1-FITC (eBiosciences, MAR-1), CD117-PE-CF594 (BD, 2B8), TNF-α-APC (Biolegend, Mp6-XT22). The antibody cocktail without monensin included NKp46-PE-Cy7 (eBiosciences, 29A1.4), CD90.2 (Biolegend, 30-H12), ST2-PE (Mdbiosciences, DJ8), KLRG1-BV605 (BD, 2F1), IL-17Rb-BV510 (BD, 6B7), CD45-PerCp (Biolegend, 30-F11), CD4-AF700 (BD, RM4-5), TCRab-APC (Biolegend, H57-597), GATA3-PerCp-eFluor 710 (eBiosciences, TWAJ), Tbet-PE-CF594 (BD, O4-46), Rorgt-BV650 (BD, Q31-378), and lineage cocktail [B220-FITC (eBiosciences, RA3-6B2), Ter-119-FITC (BioLegend, TER-119), CD8a-FITC (BD, 53-6.7), CD49b-FITC (eBiosciences, HMa2), Nk1.1-FITC (BD, PK136), CD11b-FITC (eBiosciences, M1/70), CD11c-FITC (BD, HL3), FceR1-FITC (eBiosciences, MAR-1)]. For intracellular cytokine (TNF-a) and intranuclear (GATA-3, T-bet, Rorgt) staining, a fixation/permeabilization solution kit (BD Cytofix/Cytoperm, 554714) and FoxP3/Transcription Factor staining buffer (eBiosciences, 00-5523-00) were used according to manufacturer’s instructions, respectively. The immune cell samples were analyzed on an LSR II (BD Biosciences) with a four laser (355nm, 405nm, 488nm and 633nm) configuration. All data were analyzed using FlowJo software (V10.0.7, Treestar). Gating strategies are shown in **Figure S4**.

### RNA Extraction for RT-qPCR and RNA Sequencing

Single-cell epithelial suspensions were prepared, stained and sorted into live EpCAM+CD45+ Siglec-F+ and live EpCAM+CD45+ Siglec-F- small intestinal epithelial cell populations to enrich for tuft cells in the EpCAM+CD45+ Siglec-F+ population as described above. Total RNA was isolated from cells using Quick-RNA Microprep Kit (Zymogen, R1050, and R1051). RNA concentrations were determined using a Qubit 2.0 fluorometer (ThermoFisher Scientific), and quality checked using a Bioanalyzer (HS RNA Pico Bioanalyzer chip, Agilent). Reverse transcription of RNA was performed with 5.6 μL of purified RNA and the resulting cDNA was amplified following the Smart-seq2 protocol with minor modifications. The primers and reagents listed in **Table S2** were used. Briefly, cDNA synthesis reactions were run as follows: incubating the RT Master mix and input RNA (72°C for 3 min), addition of template switching oligo master mix to the reaction mixture, reverse transcription (42°C for 90 min, followed by 10 cycles (50°C for 2 min, 42°C for 2 min), and inhibition of the reaction (70°C for 15 min). Afterwards, cDNA amplification was performed by incubating the cDNA and cDNA preamplification master mix at 98°C for 3 min, then 19 cycles (98°C 15s, 67°C 20s, 72°C 6 min), with a final extension at 72°C for 5 min using the PCR cycler and reagents (**Table S2**). Resultant cDNA was purified using AMPure XP beads (Beckman Coulter, A63881), and subsequently quantified using Qubit HS dsDNA Assay Kit (Life Technologies), checked for quality and library size using a High-Sensitivity DNA chip (Agilent Bioanalyzer) and used for quantitative PCR (qPCR) and RNA sequencing.

The qPCR of the amplified cDNA obtained from the two fractions of intestinal epithelial cells was performed with TaqMan Fast Universal PCR Master Mix (Applied Biosystems, 4352042) and a 7900HT Fast Real-time PCR system (Applied Biosystems) using primers and probes (**Table S3**) purchased from Integrated DNA Technologies (Leuven, Belgium). The PCR reactions were run under the following conditions: 95°C for 20 sec; 40 cycles of 95°C for 1 sec and 60°C for 20 sec. Transcripts were normalized to either *Gapdh* or *Dclk1* expression and relative expression was calculated based on ΔCt.

For RNA sequencing, the sequencing libraries from amplified cDNA of enriched small intestinal tuft cells were generated using the Nextera XT DNA library preparation kit with multiplexing primers, according to the manufacturer’s protocol (Illumina, FC-131-1096). Resultant cDNA libraries were purified using AMPure XP beads (Beckman Coulter, A63881), quantified using Qubit HS dsDNA Assay Kit (ThermoFisher Scientific), checked for library fragment size distributions using the High-Sensitivity DNA chip (Agilent Bioanalyzer). Afterwards, libraries (having insert size of 200-700 bp) were subjected to paired-end sequencing on the Illumina NovaSeq 6000 sequencing platform (Novogene Corporation, China). For analysis, the raw data were de-multiplexed and then filtered using Cutadapt (v1.15) to remove sequencing adapters and low quality reads. Mapping and alignment of the clean reads were performed using the STAR software program (v2.6.0) and transcript quantification was determined using the RSEM software package. The resulting gene matrix contained 47,729 genes of which 11,557 genes were not expressed in any samples and removed. Of the remaining 36,172 genes, 79 genes had duplicate gene symbols, and only the gene with the highest total read count was retained. To filter out lowly expressed genes, we filtered out genes that did not have more than five reads in at least six samples, leaving 14,015 genes for further analysis. Cell type deconvolution was made using BSEQ-sc v1.0 and CIBERSORT. To remove gene count contributions from contaminating cell types, we weighed the read counts of all genes that were expressed by multiple cell types in Haber et al. (20), by the cell type proportion estimated by BSEQ-sc. Furthermore, using the log10 read count distribution of Haber et al. (20), we estimated the mode and the lower and upper read count cutoff that would contain 95% of the reads below and above the mode, respectively. Using the lower cutoff to exclude genes whose read counts were too low to confidently estimate tuft cell read proportions, we estimated the proportion of reads that originated from tuft cells and used this factor as an additional weight parameter when removing contaminating reads. Finally, we used the read count distribution of non-tuft cell marker genes [as identified in (20)] to estimate which genes that were not expressed in tuft cells and thus could be removed; there were 853 genes with at least three reads in at least 20% of any non-tuft cell types and with at least three reads in less than 20% of tuft cells, and together with the non-tuft marker genes, these were removed from the dataset. Genes were annotated with pathway information using Reactome, and differentially expressed genes and pathways were identified using SAM-seq v.1.0. Differentially expressed genes that were not part of any Reactome pathway (termed orphan genes) were designated immune/metabolism-associated if they were annotated with either the GO term “GO:0008152 metabolic process” or “GO:0002376 immune system process”.

### Tissue Cytokine Analysis

During mice dissection small intestinal and colon (flushed and mesenteric fat free) and eWAT tissue samples were harvested and snap frozen at -80°C until further analysis. For cytokine analysis approximately 50 mg of tissues were weighed, added into the extraction buffer (100 mg tissue/mL extraction buffer (w/v) containing 1 protease-inhibitor cocktail tablet [Complete ULTRA, Roche (05892791001)] per 5 mL PBS (Mg^2+^ and Ca^2+^-free, pH 7.4, Gibco, 14190) and homogenized using a homogenizer (Biospec 1001) until a uniform tissue homogenization was achieved. Afterwards, the homogenized mixture was centrifuged at 500g at 4°C for 10 min, and the supernatant was collected and stored at -80°C until analysis. Homogenate concentrations of IL-4, IL-5, IL-13, IL-25, IL-33 and TNF-α cytokines were measured using electro chemoluminescence measurements based on the Meso Scale Discovery platform (Meso Scale Discovery) according to the manufacturer’s instructions. Cytokine levels were calculated as pg per g of tissue.

### Statistical Analysis

Statistical analyses were performed in Prism 8.0.2 (GraphPad Software) and R v3.5.2 (R Core Team, 2018). While using Prism, the Holm-Sidak method was used to correct for multiple comparisons with *p < 0.05, **p < 0.01, ***p < 0.001. Unless otherwise stated, Spearman’s rank correlation was used for correlations with p-values < 0.05 and q-values < 0.1 as statistically significant. Network analyses were based on Spearman rank correlation coefficients (SCC) with (SCC) > 0.7 or SCC < -0.7 and q-values < 0.05 as statistically significant. Correlation networks are shown using Cytoscape (v. 3.8.2).

## Results

### Diet-Induced Obesity Reduces Expression of *Il25* and *Tslp* in Small Intestinal Tuft Cells

**Figure 1:**
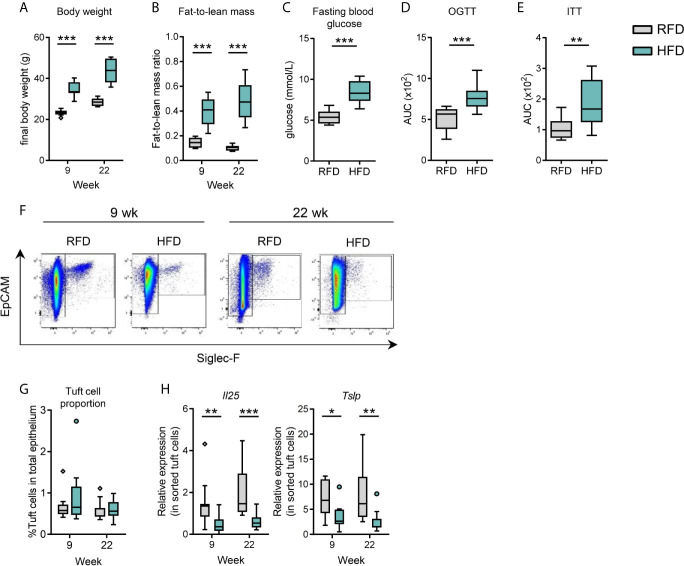
Diet-induced obesity results in decreased small intestinal tuft cell activity. C57BL/6J mice were fed either RFD or HFD for 9 and 22 weeks, respectively. **(A)** Body weight and **(B)** fat-to-lean mass ratio at week 9 and 22. **(C)** Fasting blood glucose at week 20, **(D)** oral glucose tolerance test (OGTT) at week 20, and **(E)** insulin tolerance test (ITT) at week 21 in RFD and HFD. **(F)** Flow cytometry-based analysis of the number of small intestinal tuft cells (EpCAM+ Siglec-F+). **(G)** Tuft cells quantified as % of tuft cells out of the total number of EpCAM+ epithelium cells. **(H)** Expression of *Il25* and *Tslp* mRNA (relative to the tuft cell marker *Dclk1*) as determined by RT-qPCR in sorted intestinal tuft cells. N=10-12 per group, derived from three repeated experiments. *p < 0.05, **p < 0.01, ***p < 0.001 by Mann-Whitney *U* test. Graphs depict boxplots showing median (center line), 25th to 75th percentiles (inter-quartile range) (box limits), +/- 1.5 times IQR (Tukey whiskers) and outliers. AUC, area under the curve; RFD, reference fat diet; HFD, high fat diet.

To examine the temporal effects of diet-induced obesity on small intestinal tuft cells, we provided C57BL/6J mice housed at thermoneutrality with either a high fat diet (HFD, 60% kcal fat) or low-fat reference diet (RFD, 10% kcal fat) for 9 and 22 weeks. Although obesity was more significant at 22 weeks, both time periods were sufficient to induce significant changes in systemic metabolic parameters between HFD and RFD, as signified by total body weight and fat-to-lean mass ratio (**Figures 1A, B** and **S1A, B**), as well as changes in liver, eWAT, iWAT and BAT masses (**Figure S1C**). After 20 weeks, HFD mice also displayed elevated fasting blood glucose, and impaired glucose tolerance and insulin sensitivity (**Figures 1C–E** and **S1D**). Flow cytometry-based identification of small intestinal epithelial cells (EpCAM+SiglecF-) and tuft cells (EpCAM+SiglecF+) (**Figures 1F** and **S2A, B**) revealed no significant effect of HFD for 9 or 22 weeks on the proportion of tuft cells out of total epithelial cells (**Figure 1G**), although HFD significantly decreased the absolute number of intestinal tuft cells at 9 (*p*=0.0016) and 22 weeks (*p*<0.0001) (**Figure S2C**). The lack of effect of HFD on tuft cell-to-epithelial cell proportions was due to a simultaneous decline in the absolute number of total epithelial cells at 9 (*p*=0.0023) and 22 weeks (*p*=0.0006) in HFD *vs.* RFD (**Figure S2C**). Among intestinal epithelial cells, tuft cells are the principal source of the type 2 immune-inducing proteins IL-25 (2–4) and thymic stromal lymphopoietin (TSLP) (20). HFD feeding for 9 and 22 weeks significantly reduced tuft cell-specific *Il25* (*p*=0.0032 for 9 weeks, *p*=0.0001 for 22 weeks) and *Tslp* (*p*=0.0233 for 9 weeks, *p*=0.0026 for 22 weeks) mRNA levels in FACS-purified tuft cells as normalized to the mRNA level of the tuft cell marker *Dclk1* (**Figure 1H**). Similarly, transcript levels of *Il25* (*p*=0.0002 for 9 weeks, *p*=0.0002 for 22 weeks) and *Tslp* (*p*=0.0019 for 9 weeks, *p*=0.0002 for 22 weeks) were significantly reduced compared to RFD using *Gapdh* mRNA for normalization (**Figure S2D**). The absolute tuft cell number correlated with *Il25* (*p*=0.006 for 9 weeks, *p*=0.005 for 22 weeks) and *Tslp* (*p*=0.001 for 9 weeks, *p*=0.019 for 22 weeks) expression levels (normalized to *Dclk1*) at both 9 and 22 weeks (**Figures S2E, F**).

### Temporal Diet-Induced Changes in the Transcriptional Profile of Small Intestinal Tuft Cells Identify Involvement of Several Gut-Brain Signaling Relays

**Figure 2:**
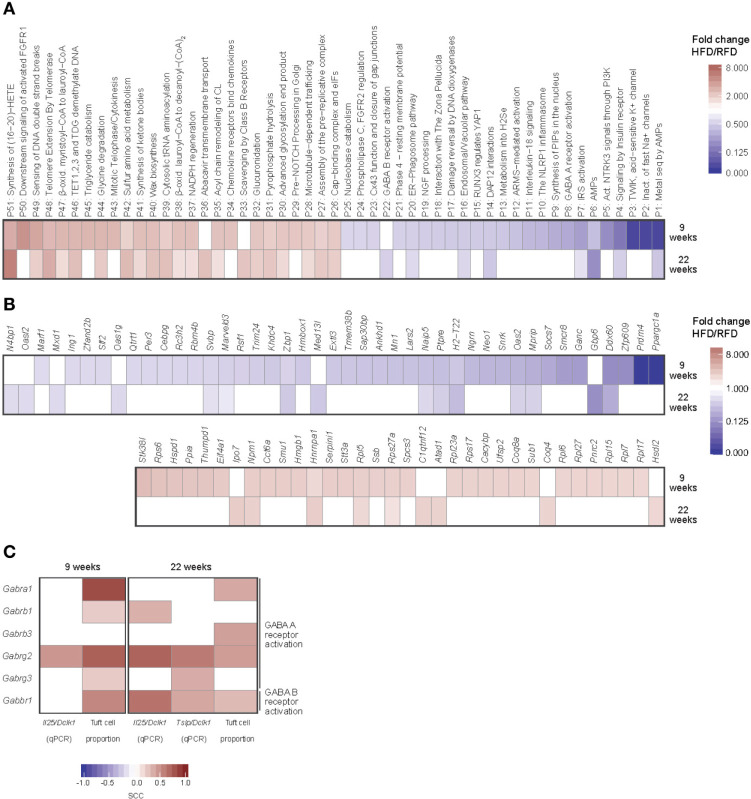
Induction of distinct transcriptional changes in small intestinal tuft cells during diet-induced obesity development. RNA-seq-based transcriptional profiles of small intestinal tuft cells purified by FACS after 9 and 22 weeks of HFD or RFD feeding. **(A)** Significantly up- (red) or down-regulated (blue) pathways (*q-*value < 0.05, fold change HFD/RFD > 2 or < 0.5), and **(B)** immune and/or metabolism relevant orphan genes, arranged by fold change. **(C)** Correlation analysis of expression levels of GABA receptor A and B encoding genes in tuft cells *vs* tuft cell numbers and expression levels of *Il25* and *Tslp* (both relative to *Dclk1*). Spearman’s rank correlation was used for correlation analysis, with *q*-values < 0.1 considered statistically significant. The inserted color codes indicate the direction of the correlation (red: positive, blue: negative). N=10-12 per group, derived from three repeated experiments. SCC, Spearman correlation coefficient; RFD, reference fat diet; HFD, high fat diet.

Using bulk RNA-sequencing of FACS sorted small intestinal tuft cells, we next examined the effect of HFD on the transcriptional profile of small intestinal tuft cells. We identified an early effect of HFD on tuft cells with 1,510 genes exhibiting differential expression in tuft cells in HFD versus RFD fed mice after 9 weeks of feeding, while only 554 genes were differentially expressed after 22 weeks (**Figure S3A, Supplementary Data Sheet 1**). Of the early regulated genes, 402 (27%) were up-regulated after 9 weeks of HFD feeding and 1,108 (73%) were down-regulated by HFD feeding as compared to RFD. After 22 weeks of HFD feeding, 209 (38%) were up-regulated and 345 (62%) down-regulated. Except for one gene [Kelch domain-containing protein 3 (*Klhdc3*)], regulation of 166 of these genes was similar at both time points and all of the shared genes were similarly up- or down-regulated (**Figure S3A**). To gain further insight into the effect of HFD intake and diet-induced obesity on tuft cell biology, we performed a pathway analysis using the Reactome pathway database (21), which includes expert annotations of immune, metabolic and neuroendocrine signaling pathways, showing that after 9 weeks of HFD, 480 pathways were significantly regulated, of which 219 were up-regulated and 261 down-regulated compared to RFD (**Supplementary Data Sheet 2**). At 22 weeks, 196 pathways were significantly regulated, out of which 157 were up-regulated and 39 down-regulated compared to RFD. Many of the significantly up-regulated pathways were associated with lipid metabolism, corresponding to adaptation of the cells to the HFD condition (**Figure 2A**).

We next examined the differentially regulated genes that were not associated with a pathway in the Reactome pathway database (here referred to as orphan genes, **Supplementary Data Sheet 3**). Since tuft cells have been suggested to relay immune-metabolic signals in the gut-brain axis (1, 22), we next focused on the differentially regulated genes in the subset of pathways annotated as relevant for immune responses and/or metabolism. Out of a total of 337 (9 weeks) and 74 (22 weeks) orphan genes (with *q*-value < 0.1), we identified 174 (9 weeks) and 28 (22 weeks) such genes based on GO term annotation of the murine genome. When filtering for *q*-value < 0.05 and fold change [(HFD/RFD) > 2 or < 0.5)], 34 of these were up-regulated at one of the time points and 43 were down-regulated (**Figure 2B**). Intriguingly, we found a strong down-regulation of *Ppargc1a* after 9 weeks of HFD. This was not seen after 22 weeks of feeding due to decreased repression of the gene in the HFD group (**Figure S3B**). Altogether, gene expression in tuft cells was found to be most strongly affected by 9 weeks of HFD feeding and to adapt to HFD conditions after prolonged feeding.

To complement the orphan gene analysis, we also identified the differentially expressed and immune-/metabolism-relevant genes that were assigned to Reactome pathways and found genes in the pathways for γ-aminobutyric acid (GABA) A and GABA B receptor activation, DNAX activation protein of 12kDa (DAP 12) interactions, and specific eicosanoid synthesis of 19(S)-hydroxy-eicosatetraenoic acid (19-HETE) and 11,12-epoxyeicosatrienoic acid (11,12-EET). Out of these four pathways, only the two GABA receptor activation pathways were also differentially regulated at the overall pathway level at both time points (**Figure 2A**). Thus, the GABA receptor activation pathways represented the most robust signature of the transcriptional changes induced in tuft cells by HFD conditions. To examine how the regulation of the genes involved in GABA A or B receptor activation linked to small intestinal tuft cell numbers and activity under HFD conditions, we correlated tuft cell numbers as well as the tuft cell signature genes *Il25* and *Tslp* with the genes implicated in these pathways (**Figure 2C**). Tuft cell numbers correlated positively with expression of genes in the GABA A and B receptor both after 9 and 22 weeks, implying a link between tuft cell numbers and expression of genes involved in GABA receptor signaling. After 9 weeks, tuft cell-specific *Il25* levels showed positive correlation with expression levels of genes in the GABA A activation pathway, whereas *Tslp* expression levels did not correlate with any of the genes (data not shown). In contrast, both *Il25* and *Tslp* expression levels at 22 weeks showed positive correlation with transcript levels of genes in all of the pathways. Combined, our findings pointed to a relationship between reduced expression of *Il25* and *Tslp* and especially the GABA receptor A receptor transcript *Gabrg2* and the GABA B receptor transcript *Gabbr1* after prolonged HFD feeding.

### The Small Intestine Represents a Robust Non-Inflammatory Immune Compartment in Response to Intake of a High Fat Diet and Obesity Development

**Figure 3:**
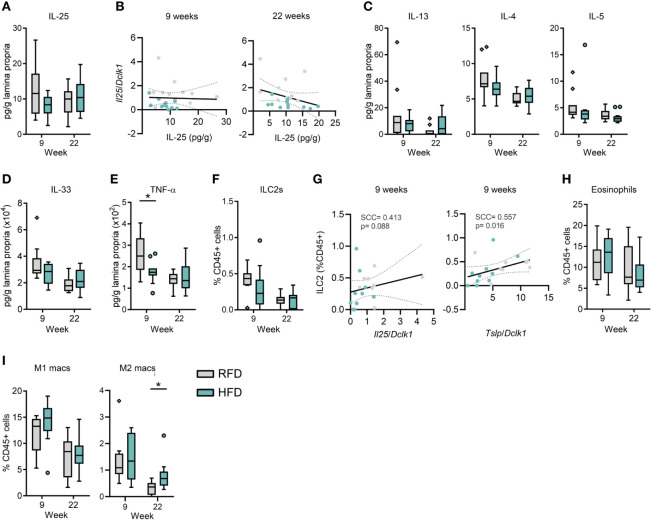
The small intestinal immune system remains homeostatic during diet-induced obesity despite changes in tuft cell numbers and activity. **(A)** Concentration of IL-25 protein levels in ileal tissue. **(B)** Spearman rank based correlation analysis between expression levels of (*Il25 *relative to *Dclk1*) in purified tuft cells and ileal IL-25 protein levels upon 9 weeks and 22 weeks of HFD or RFD. Protein concentrations of **(C)** IL-13, IL-4 and IL-5, **(D)** IL-33, and **(E)** TNF-α in ileal tissue. **(F)** ILC2 quantification (as % of total CD45+ cells) by flow cytometry of small intestinal lamina propria cells. **(G)** Spearman rank-based correlation analysis between proportions of ILC2s (as % of total CD45+ cells) and expression levels of *Il25* and *Tslp* (both relative to *Dclk1*) in purified tuft cells after 9 weeks of HFD or RFD. Quantification of **(H)** eosinophils, **(I)** M1 and M2 type macrophages (as % of total CD45+ cells) by flow cytometry of small intestinal lamina propria cells. N=10-12 per group, derived from three repeated experiments. *p < 0.05 by Mann-Whitney *U* test. Graphs depict boxplots showing median (center line), 25th to 75th percentiles (inter-quartile range) (box limits), +/- 1.5 times IQR (Tukey’s whiskers) and outliers. Spearman’s rank correlation was used for correlation analyses with *p*-values < 0.05 considered statistically significant. SCC, Spearman correlation coefficient; RFD, reference fat diet; HFD, high fat diet.

Given that IL-25 is involved in propagating type 2 immune responses in the gut *via* activation of IL-25 receptor (IL-17RB)-expressing ILC2s (2–4), we next examined the HFD-induced changes in the immune phenotype of the small intestinal lamina propria with a focus on type 2 immune response-related cytokines and immune cells. Although IL-25 is known to be produced not only by tuft cells but also by lamina propria cells (23), it was surprising to find that HFD feeding for 9 or 22 weeks did not affect IL-25 protein levels in the ileum (**Figure 3A**), neither did sorted tuft cell-specific *Il25* mRNA levels correlate significantly with IL-25 protein levels in the ileum after 9 and 22 weeks of HFD feeding (**Figure 3B**). Furthermore, no significant changes were found in the ileal concentrations of the immune cell-derived cytokines IL-13, IL-4 and IL-5 (**Figure 3C**) or in epithelial/fibroblast/endothelial cell-derived IL-33 (**Figure 3D**). Notably, ileal levels of the pro-inflammatory cytokine TNF-α were significantly decreased at 9 weeks (*p*=0.0128) after the onset of HFD feeding and remained low even after HFD feeding for 22 weeks (**Figure 3E**).

We next addressed if HFD feeding affected the proportions of immune cell subsets in the small intestinal lamina propria (**Figures S4A–C** for gating strategy). In accordance with the overall cytokine levels, we did not find any significant changes in the proportion of ILC2s in the small intestinal lamina propria after HFD feeding for 9 or 22 weeks (**Figure 3F**). Since ILC2s can be activated by IL-25, IL-33, and TSLP, we examined if proportions of small intestinal ILC2s correlated with *Il25* mRNA and *Tslp* mRNA levels in sorted tuft cells and concentrations of IL-25 and IL-33 in the ileum. Whereas the proportions of ILC2s in the small intestine did not correlate significantly with *Il25* mRNA levels in sorted tuft cells (SCC=0.413, *p*=0.088), they correlated with *Tslp* mRNA levels (SCC=0.557, *p*=0.016) (**Figure 3G**) after 9 weeks of HFD feeding, but not after 22 weeks (SCC=-0.293, p=0.197; **Figure S5A**). Similarly, small intestinal ILC2 proportions did not correlate with IL-25 (SCC=-0.265, p=0.287; **Figure S5B**) and IL-33 (SCC=-0.557, p=0.813; **Figure S5C**) levels in the ileum at 9 and 22 weeks. Activated intestinal ILC2s produce IL-5 that promotes eosinophilia (24). As expected from the unchanged ILC2 and IL-5 levels, intestinal eosinophil proportions were unchanged by HFD feeding for 9 and 22 weeks (**Figure 3H**). Furthermore, eosinophil proportions in the small intestine did not correlate with *Il25* (SCC=0.090, p=0.695, 9w, **Figure S5D**) and *Tslp* (SCC=0.187, p=0.417; **Figure S5E**) mRNA levels in sorted tuft cells at 9 or 22 weeks. HFD feeding is generally associated with an increased proportion of M1 type pro-inflammatory and decreased proportions of M2 type anti-inflammatory macrophages in adipose tissues (25). However, M1/M2 proportions in the small intestinal environment are poorly characterized under HFD feeding conditions. In the small intestine, the proportion of the M1 type pro-inflammatory macrophages was not different after 9 and 22 weeks of RFD and HFD feeding, whereas the proportion of M2 type anti-inflammatory macrophages was significantly increased under HFD conditions at 22 weeks (*p*=0.0109), although generally smaller than the levels at 9 weeks (**Figure 3I**). Notably, no significant changes were seen for most of the other intestinal immune cell subsets; monocytes, macrophages, dendritic cells, T-helper (Th) and ILCs after 9 and 22 weeks of HFD feeding (**Figures S5F–K**). Amongst the identified populations, the non-ILC1/2/3 and non-Th1/2/17 immune cell populations may include regulatory ILCs and Tregs, respectively, but these were not identified by specific markers in the current study.

Sub-clinical inflammation in eWAT, manifested by local alterations in immune cell composition, is a key marker and driver for metabolic dysregulation. In addition, intestinal helminth infection drives a type 2 immune phenotype in eWAT *via* ILC2s and eosinophils that ameliorates obesity-associated metabolic dysfunction (26, 27). As expected from previous studies (25), the pro-inflammatory environment in eWAT was amplified over time with enhanced levels of M1 type macrophages and CD4+RORγT+Tbet-, defining Th17 cells, at 9 weeks, while TNF-α, total dendritic cells and their subsets (CD11b^int^, CD11b^hi^) increased after 22 weeks of HFD feeding (**Figures S6A–G**). The anti-inflammatory M2 macrophages were also temporally enhanced by HFD at 9 weeks (**Figure S6B**). These changes occurred at the expense of monocytes and eosinophils that were significantly decreased at both 9 and 22 weeks, and ILCs after 22 weeks of HFD feeding (**Figures S6D, G**). Overall, these data indicate that HFD exerts stronger temporal immunomodulatory changes in eWAT in comparison to the small intestinal environment during 22 weeks of feeding.

### Distinct Small Intestinal Tuft Cell Transcripts Link Strongly to Obesity-Related Metabolic Parameters but Not to Inflammatory Markers of Epididymal Adipose Tissue

**Figure 4:**
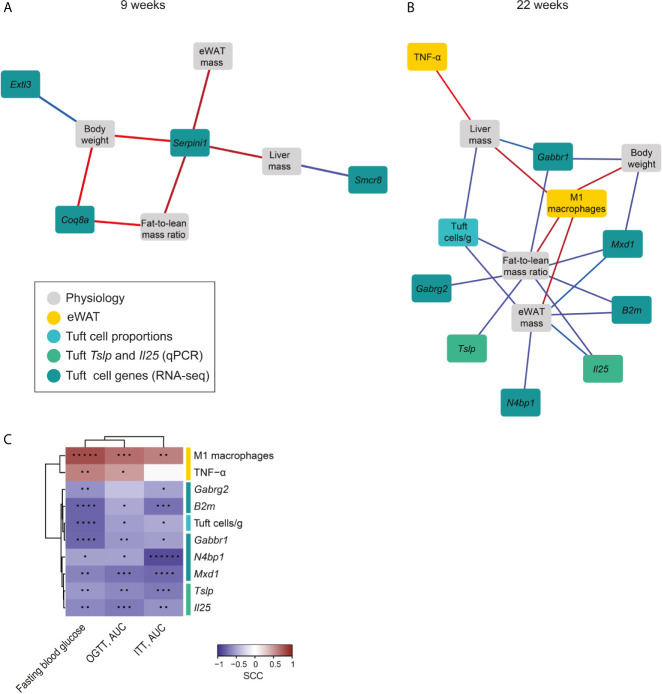
Network analysis interconnects small intestinal tuft cell responses with systemic diet-induced metabolic changes. Network analysis of identified highly significant factors from small intestinal tuft cells (numbers and transcripts), key immune cell subsets (ILC2, eosinophils, M2 and M1 macrophages), tissue cytokines and metabolic parameters (total body, eWAT and liver weight and fat-to-lean mass ratio) at 9 **(A)** and 22 weeks **(B)** after onset of HFD feeding. The networks display the strongest statistically significant correlations, 0.7<SCC<-0.7, *q-*value<0.05, illustrated using Cytoscape. Associations are presented by the color of the lines, where red represents positive and blue negative correlations, and the intensity of the color represents the strength of the correlation. **(C)** Correlation analysis between the 22 weeks markers and fasting blood glucose (at w20), glucose (at w20) and insulin tolerance levels (at w21) in mice fed HFD for 22 weeks. The heatmap shows spearman rank correlation coefficients (SCC; blue: negative, red: positive). Statistically significant correlations are noted by an asterisk (*p < 0.05; **p < 0.01; ***p < 0.001; ****p < 0.0001; *****p < 0.00001; ******p < 0.000001). N=10-12 mice per group from three repeated experiments.

We next examined if the HFD-induced changes in tuft cell transcripts linked to the identified changes in eWAT immune responses and metabolic parameters. We found numerous correlations, and therefore decided to narrow down the analysis to include only the most significant of these associations (0.7<SCC<-0.7, and q<0.05) after 9 weeks and 22 weeks of feeding, respectively to highlight only the most important relationships in a network analysis (**Figure 4**). We found a larger number of associations between small intestinal tuft cell gene expression and body mass parameters at 22 weeks compared to 9 weeks (**Figures 4A, B**). After 9 weeks of HFD, tuft cell expression of exostosin-like glycosyltransferase 3 (*Extl3*) negatively associated with total body weight, and the SMCR8-C9orf72 complex subunit (*Smcr8*) negatively associated with liver mass, whereas total body weight and fat-to-lean mass ratio both associated positively with tuft cell expression of coenzyme Q8A (*Coq8a*) (**Figure 4A**). Notably, all metabolic parameters associated positively with tuft cell expression of neuroserpin (*Serpini1*), which is exclusively expressed in intestinal tuft cells (20), indicating that HFD-induced enhancement of *Serpini1* expression may influence obesity development. After 22 weeks of HFD, the network revealed well known positive associations between body, liver and eWAT mass and fat-to-lean mass ratio and eWAT-M1 macrophages as well as positive associations between liver mass and eWAT-TNF-α (**Figure 4B**). Intriguingly, the absolute number of small intestinal tuft cells associated strongly negatively with liver and eWAT mass and fat-to-lean mass ratio. Since these associations are based on correlation analysis using data from individual mice, this finding is not just a coincidence stemming from higher and lower numbers of both factors in RFD *vs.* HFD, respectively. The expression level of several genes downregulated by HFD in tuft cells also correlated negatively with the body composition parameters; *Il25* and *B2m*, involved in DAP12, both associated inversely with eWAT mass and fat-to-lean mass ratio. *Tslp* likewise associated inversely with fat-to-lean mass ratio, *Gabbr1* associated inversely with fat-to-lean mass ratio and total body and liver mass, *Gabrg2* associated inversely with fat-to-lean mass ratio, *Mxd1* associated inversely with fat-to-lean mass ratio and total body and eWAT mass, and *N4bp1* associated inversely with eWAT mass.

To examine how the tuft cell-related factors that linked with the specific tissues (i.e. liver mass, body weight, fat-to-lean mass ratio and eWAT mass) associated with systemic regulation of glucose metabolism, we next examined the relationship between these factors upon 22 weeks of HFD feeding and fasting blood glucose levels and glucose tolerance and insulin tolerance, determined at 20 and 21 weeks, respectively. We found that tuft-cell related factors were also linked to regulation of glucose metabolism, with eWAT pro-inflammatory markers (M1 macrophages and TNF-α) being positively associated to fasting blood glucose and OGTT after 22 weeks HFD feeding (**Figure 4C**). Additionally, eWAT-M1 macrophages also correlated significantly with an impaired insulin response. Notably, all the tuft cell parameters showed significantly inverse correlations with fasting blood glucose, glucose and insulin responses, except for *Gabrg2*, which as the sole factor did not correlate significantly with glucose tolerance. Combined this showed that HFD feeding for 22 weeks resulted in reduced expression of the type 2-response mediators *Il25* and *Tslp* in small intestinal tuft cells with concomitant and associated reduction in expression of the transcripts for GABA A and B receptors, *Gabrg2* and *Gabbr 1*, in small intestinal tuft cells (the latter shown in **Figure 2C**). Improved insulin tolerance and blood glucose levels, and lower weights of liver, eWAT, total body mass and fat-to-lean mass ratio all correlated strongly to enhanced levels of these four tuft cell-derived transcripts, hence linking tuft-cell derived type 2 regulation and GABA receptor expression to metabolic regulation. The small intestinal tuft cell association to metabolic parameters was found to be independent of type 2 immune cell involvement in both the small intestinal lamina propria and in eWAT.

## Discussion

We here demonstrated an effect of HFD feeding on small intestinal tuft cell biology that links to systemic metabolism independently of immune cell involvement. We identified differential and temporal expression of >1,700 genes in small intestinal tuft cells in response to intake of HFD and diet-induced obesity development. Notably, we found that several of the tuft cell expressed genes, including genes involved in the gut-brain axis such as the GABA receptor pathways, associated strongly with whole-body and glucose metabolism, while they did not associate with the comprehensively analyzed small intestinal and eWAT immune parameters. This suggests the existence of a small intestinal tuft-cell-to-whole-body metabolic relay that involves gut-brain signaling without contribution of intestinal or eWAT residing immune cells. Moreover, identification of small intestinal tuft cell-derived *Serpini1* as an early marker of HFD intake and obesity development points to the involvement of a direct tuft cell-neuro circuit in diet-induced obesity.

*Serpini1* encodes the serine protease inhibitor neuroserpin, previously identified in neurons (28), endocrine tissues (29), and immune cells (30). However, based on information from single cell sequenced intestinal cells, in the gut *Serpini1* is expressed exclusively in tuft cells (20). This finding supports the idea of intestinal tuft cells acting as epi-neuronal cells capable of relaying signals between gut epithelial cells and the brain, as intestinal tuft cells are known to also express other markers than *Serpini1* that affect afferent and efferent nerve synapses (31). While we were unable to find previous reports regarding the role of *Serpini1* in intestinal tuft cells, brain-derived neuroserpin is reported to be involved in mood regulation, where both under- and over-expression of neuroserpin in the brain results in development of depression-like symptoms in mice (32). Whether or not HFD-induced intestinal tuft cell expression of *Serpini1* may play a part in HFD-induced depression (33) or other neurodegenerative disorders like Parkinson’s disease, Alzheimer’s disease, multiple sclerosis, and amyotrophic lateral sclerosis (34) will need further investigations. We identified a significant elevation in neuroserpin expression at 9 weeks, but not at 22 weeks after onset of HFD feeding. A possible role for neuroserpin in upregulation of lipid absorption in small intestinal enterocytes might explain this difference as early adaptation to HFD may mediate increased fat storage (35–37), while longer periods of HFD intake may result in decreased neuroserpin expression to mitigate long-term metabolic consequences.

The neurotransmitter GABA, acting through its receptors GABA A and GABA B, stimulates feed intake (38), but has also been shown to ameliorate metabolic dysregulation under HFD conditions, such as insulin resistance and glucose intolerance (39). We found that HFD feeding resulted in significantly reduced expression of genes encoding the subunits of both of these GABA receptors in small intestinal tuft cells, possibly reducing GABA-mediated activation, suggesting that intestinal tuft cells might hold nutrition-adaptive properties during HFD conditions. The intestinal presence of tuft cells in close contact with enteroendocrine cells and nerve fibers involved in satiety induction (1) supports this notion. In addition to this, the intestinal gut microbiota have also been reported to produce GABA *in vitro*, including *Bacteroides*, *Parabacteroides*, *Eubacterium* and *Bifidobacterium* spp. (40), but also *Lactobacillus brevis* and *Bifidobacterium dentium* (41). Indeed, oral administration of GABA-producing *Lactobacillus brevis* for 10 weeks to obese mice improved systemic glucose and cholesterol levels (42).

Amongst all intestinal epithelial cells, tuft cells are the only source of the cytokine IL-25 (20). Since HFD feeding resulted in decreased tuft cell specific transcript levels of *Il25* and *Tslp*, the tuft-IL-25-ILC2 circuit would likely be disrupted. Still, our observations suggest the presence of additional intestinal signals besides the tuft cell-derived IL-25 that affect the tuft-IL-25-ILC2 circuit. One such may relate to tuft cell-derived TSLP for which we identified an association to the small intestinal ILC2 proportion. This finding is further supported by the fact that even in intestinal epithelial specific *Il25* ^-/-^ mice, small intestinal lamina propria ILC2s were not completely absent (4). Furthermore, IL-25 secreted from non-tuft cells, such as macrophages and eosinophils (23), could be involved in maintaining an IL-25-ILC2 circuit during HFD feeding conditions, although we did not observe any associations between ileal IL-25 protein levels and ILC2 proportions. Since we identified no effect of HFD feeding on the small intestinal lamina propria ILC2 or eosinophil populations, nor on production of type 2 cytokines, the HFD effects on the small intestinal type 2 response seemed to be mediated alone *via* type 2 relays in small intestinal tuft cells.

In our study, the small intestine represented a robust non-inflammatory environment after 9 and 22 weeks of HFD feeding. This is in contrast to some previous studies investigating inflammation in the small intestine after HFD feeding in C57BL/6 mice that reported type 1 proinflammatory changes in T-cells (8, 10) and eosinophil numbers (9). However, since the previous studies used normal chow diets as the reference control, and we here used defined diets with equal amounts of dietary fibers, it is difficult to compare our results to previous studies. While the gut immune profile presented an apparently stable environment, the phenotype in eWAT was akin to the well-established pro-inflammatory environment induced by HFD feeding (7). IL-33 has been associated with protective effects in relation to the adipose tissue during obesogenic conditions by e.g. maintaining Tregs (43) and the ILC2 pool (27). Importantly, we observed high levels of IL-33 in eWAT, but no significant changes in IL-33 levels between RFD and HFD fed mice when using these defined diets. Likewise, ILC2 and Treg proportions did not differ between RFD and HFD fed mice. This is in contrast to previous reports (27, 43), which again might be explained by the previous use of regular fiber-rich chow as the reference diet.

Combined, we here demonstrated that several key metabolic parameters related to intake of HFD and diet-induced obesity associated with decreased *Il25* and *Tslp* expression levels in small intestinal tuft cells without affecting immune homeostasis of the connected lamina propria. Rather, the small intestinal tuft cells may mediate metabolic regulatory effects *via* neuro-involvement, as exemplified by induction of the gene encoding neuroserpin and reduced expression of the GABA receptors in small intestinal tuft cells during obesogenic conditions. These findings provide important novel insight into the dietary-sensing role of small intestinal tuft cells and their relation to whole-body metabolism and offer a basis for further investigations.

## Code Availability

Please refer to Materials and Methods section.

## Data Availability Statement

RNA-sequencing data is available in NCBI GEO with accession number GSE141002.

## Ethics Statement

The animal study was reviewed and approved by The Danish Animal Experiment Inspectorate (Permission Number 2014-15-2934-01027/C2) and carried out in compliance with the ARRIVE guidelines and Danish guidelines for experimental animal welfare.

## Author Contributions

Conceived and designed the study: PA, DA, KK, and SB. Performed the experiments: PA, DA, ND-S, GK, and PR. Analyzed the data: PA (flow cytometry, metabolic phenotyping, cytokines), DA (qPCR, data integration, flow cytometry), JM, LX, and BZ (RNA-seq analysis). Wrote the paper: PA and SB. Revised the paper: JM, DA, and KK. All authors contributed to the article and approved the submitted version.

## Funding

This project was funded by the Technical University of Denmark (DTU), the Danish Research Foundation *via* the Molecular Pathology PhD Programme at DTU, and The Novo Nordisk Foundation (Reference number: NNF15OC0016406), all to SB.

## Conflict of Interest

Author KK is employed by BGI-Shenzhen.

The remaining authors declare that the research was conducted in the absence of any commercial or financial relationships that could be construed as a potential conflict of interest.
