# Supplementary material for: Small Intestinal Tuft Cell Activity Associates With Energy Metabolism in Diet-Induced Obesity
Source: Front Immunol. 2021 May 28;12:629391. doi: 10.3389/fimmu.2021.629391 (PMC8195285; doi:10.3389/fimmu.2021.629391)
Supplement: Supplementary file 1 [file DataSheet_1.docx]

**SUPPLEMENTARY MATERIAL**

**Intestinal tuft cell activity associates with energy metabolism in diet-induced obesity**

**Pankaj Arora^1¤^, Daniel Andersen^1¤^, Janne Marie Moll^1¤^, Niels Banhos Danneskiold-Samsøe^2^, Liqin Xu^1,3^, Biaofeng Zhou^3^, Georgios Kladis^1^, Philipp Rausch^2^, Christopher T Workman^1^, Karsten Kristiansen^2,3^, Susanne Brix^1*^**

^1^Department of Biotechnology and Biomedicine, Technical University of Denmark, Kgs. Lyngby, Denmark

^2^Laboratory of Genomics and Molecular Biomedicine, Department of Biology, University of Copenhagen, Copenhagen, Denmark

^3^BGI-Shenzhen, Shenzhen, China

¤ These authors contributed equally.

***Correspondence**: Susanne Brix, Department of Biotechnology and Biomedicine, Technical University of Denmark, Søltofts Plads, building 224, DK-2800 Kgs. Lyngby, Denmark; e-mail: [sbp@bio.dtu.dk](mailto:sbp@bio.dtu.dk); Telephone number: +45 45252784, ORCID: 0000-0001-8951-6705

**SUPPLEMENTARY FIGURES**

**Supplementary figure 1**

**RFD**

**HFD**

22wk OGTT and ITT

9 and 22wk organ mass

**D**

**C**

**B**

22wk body weight, fat and lean mass

**A**

9wk body weight, fat and lean mass

**Fig. S1 Metabolic characteristics of C57BL/6J mice fed HFD or RFD for 9 and 22 weeks.** Progress of body weight, fat and lean mass during (A) 9wk and (B) 22wk of experimental feeding with RFD and HFD. (C) Organ masses at the end of 9 and 22wk. (D) OGTT (at week 20) and ITT (at week 21) in the 22wk groups. N=10-12 per group, derived from three experiments. ***p* < 0.01, ****p* < 0.001 by Mann-Whitney *U* test. Graphs depict boxplots showing median (center line), upper and lower quartiles (box limits), minimum and maximum (whiskers).

iWAT mass (g)

BAT mass (g)

Cecum mass (g) (g)

eWAT mass (g)

Liver mass (g)

**
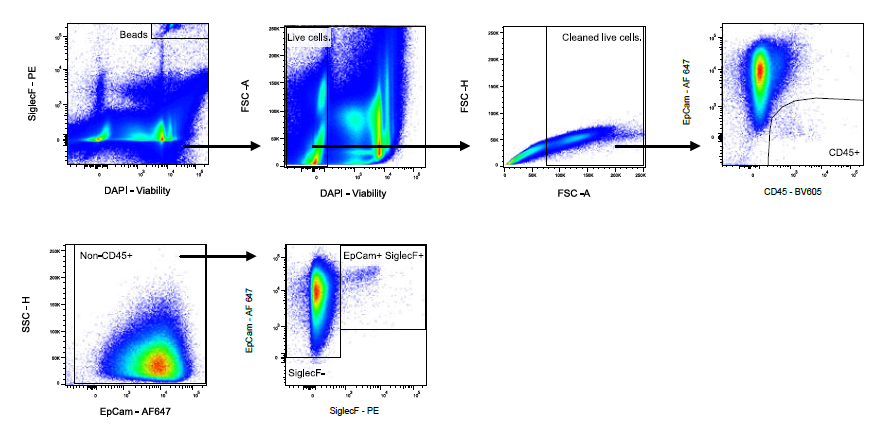
Supplementary figure 2**

**Fig. S2 HFD feeding is associated wth decreased small intestinal tuft cell number and activity in C57BL/6J mice.** Tuft cells (EpCAM+ Siglec-F+) were sorted from the small intestine (20 cm) by flow cytometry. Non-tuft intestinal epithelial cells (EpCAM+ Siglec-F-) were sorted as negative control. *Dclk1*, *IL25*, *Tslp* and *Gp2* mRNA expression was quantified by RT-qPCR in sorted non-tuft and tuft cells from C57BL/6J mice fed either HFD or RFD for 9wk and 22wk. (A) Representative gating strategy for tuft cells obtained from small intestines. (B) Indicated genes analyzed by RT-qPCR (relative to *Gapdh*) in sorted tuft and non-tuft epithelial cells. (C) Tuft and total epithelial cell quantification (as cells per g intestinal tissue) using count beads during flow cytometry. (D) Indicated genes analyzed by RT-qPCR (relative to *Gapdh*) in sorted tuft cells. Tuft cells/g intestine positively correlates with mRNA expression level of *Il25* and *Tslp* (both relative to *Dclk1*) in sorted tuft cells at (E) 9 wk and (F) 22 wk. N=10-12 per group, derived from three experiments. ***p* < 0.01, ****p* < 0.001 by Mann-Whitney *U* test followed by correction for multiple comparisons using Benjamini-Hochberg method. Graphs depict boxplots showing median (center line), upper and lower quartiles (box limits), minimum and maximum (whiskers) and outliers. Spearman’s rank correlation was used for correlations with *p*-values < 0.05 considered statistically significant. SCC: Spearman’s rank correlation coefficient.

**A**

**B**

**E**

**F**

9 weeks

22 weeks

**Tuft**

**Non-tuft**

**C**

**D**

**RFD**

**HFD**

**Supplementary figure 3**

**A**

**B**

**
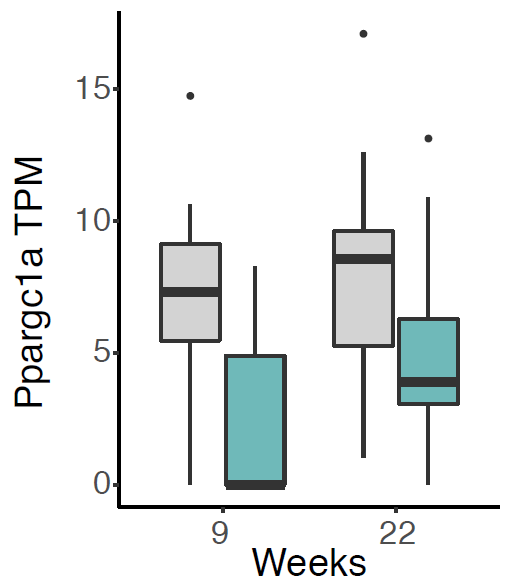
**

*p*= 0.07

*p*= 0.01

**HFD**

**RFD**

9wk

22wk

58

108

59

108

**Fig. S3 Temporal changes in gene expression in small intestinal tuft cells during HFD-fed conditions in C57BL/6J mice.** (A) Venn diagram of the temporal gene expression levels in sorted small intestinal tuft cells under HFD-fed conditions at 9 and 22 weeks, relative to RFD-fed conditions. (B) Expression levels of *Ppargc1a* in small intestinal tuft cells at 9 and 22 weeks. N=10-12 per group, derived from three experiments. Graphs depict boxplots showing median (center line), upper and lower quartiles (box limits), minimum and maximum (whiskers) and outliers.

1000

344

**22 wk**

**9 wk**

1344

387

237

150

**Supplementary figure 4**


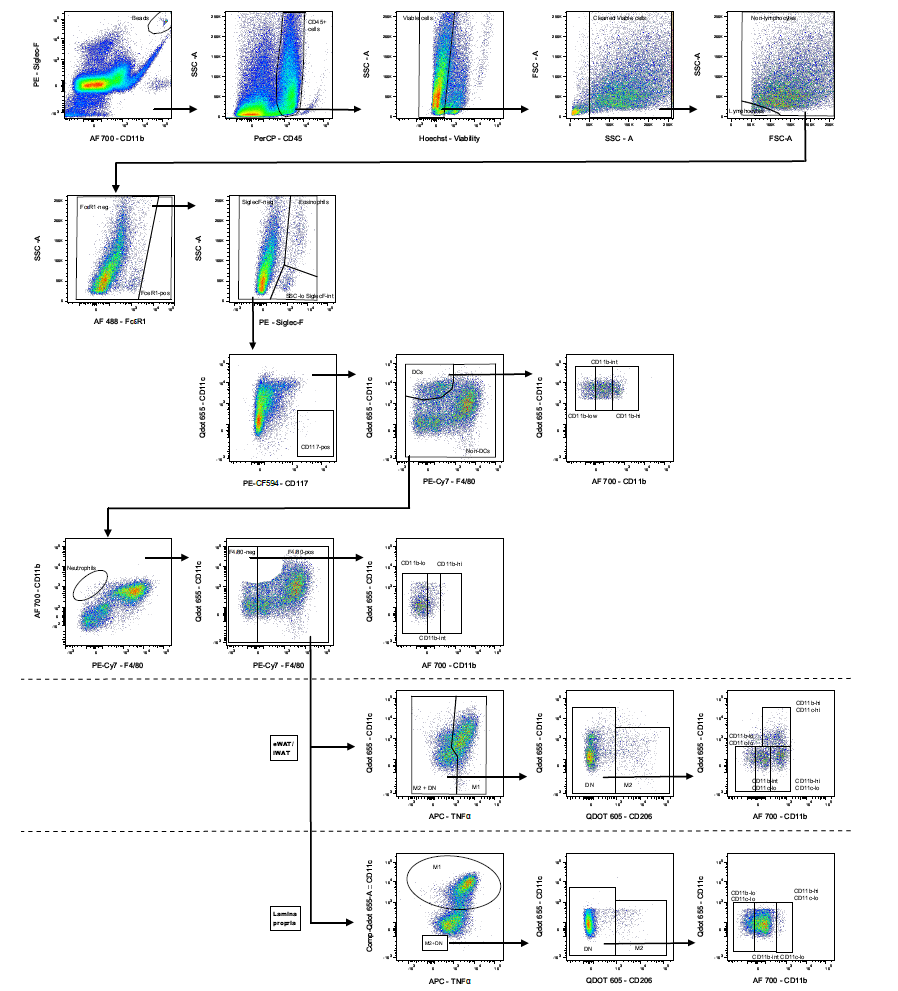


**A**


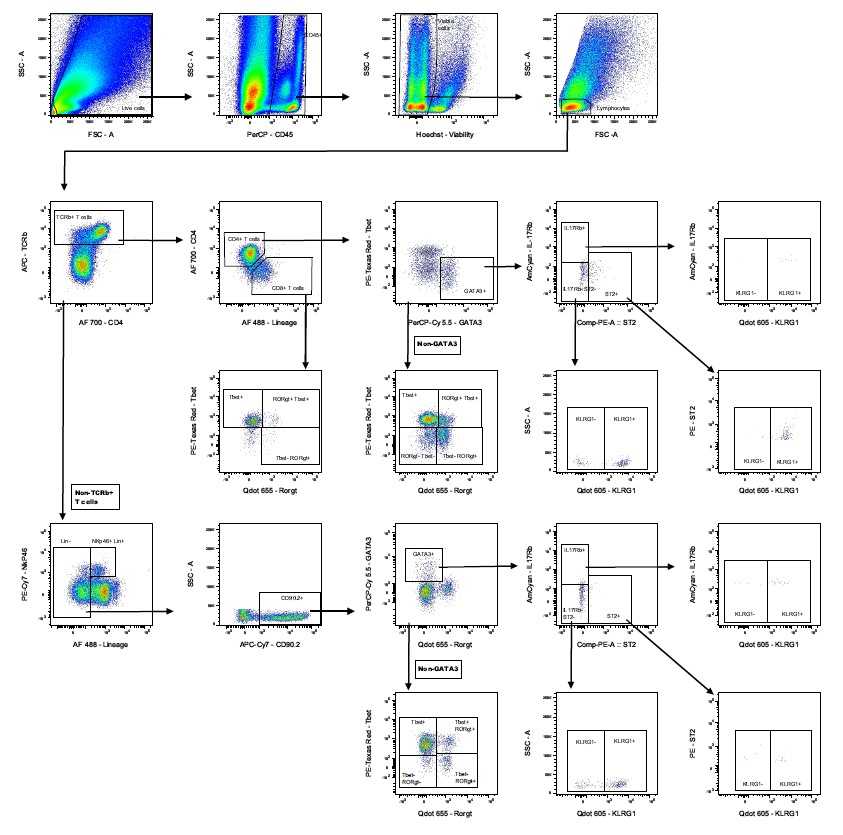


**B**

**ILCs**

**C**


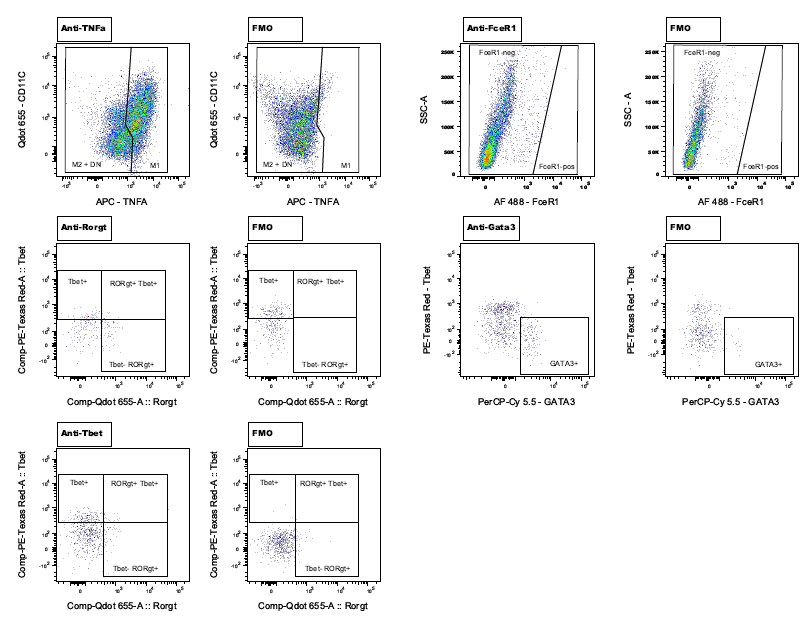


**Fig. S4 Representative flow cytometry gating strategy.** Major and minor immune cell subsets of (A) monocytes, macrophages, dendritic cells, eosinophils and neutrophils (mast cell and basophils were undetected). (B) ILC1/2/3 and non-ILC1/2/3s (~ILCreg), CD4+ Th1/2/17/Tregs and CD8+ T-cells in small intestinal lamina propria and white epididymal adipose tissue. (C) Representative intracellular and intranuclear staining and the fluorescence minus one (FMO) controls for indicated markers.

**Supplementary figure 5**

**A**

**B**

**C**

**D**

**E**

**J**

**K**

**F**

**G**

**H**

**I**

**Fig. S5 The small intestine represents an immunological robust tissue under HFD-fed conditions in C57BL/6J mice.** Immediately after study termination, the small intestine (20 cm) was processed to prepare single-cell suspensions for flow cytometry. Intestines were processed in two steps: first, epithelial cells were isolated and afterwards the remaining intestinal tissue was digested to obtain bulk lamina propria immune cells. Spearman’s rank-based correlation of the proportion of ILC2s (as % of total CD45+ cells) with (A) mRNA expression levels of *Il25* and *Tslp (*both relative to *Dclk1*) in sorted tuft cells at 22wk, (B) ileal IL-25 and (C) ileal IL-33 concentrations at 9wk and 22wk. Quantification by flow cytometry (as % of total CD45+ cells) (D) total monocytes and indicated subsets, (E) total macrophages and indicated susbsets, (F) ILCs and indicated subsets, (G) dendritic cells and indicated subsets, (H) CD4+ T-cells and indicated subsets, and (I) CD8+ T-cells in SI lamina propria. Correlation between the proportion of eosinophils (as % of total CD45+ cells) with mRNA expression levels of (J) *Il25* and (K) *Tslp (*both relative to *Dclk1*) in sorted tuft cells at 9wk and 22 wk. N=10-12 per group, derived from three experiments. Statistical analyses were performed by Mann-Whitney *U* test followed by correction for multiple comparisons using Benjamini-Hochberg method. Graphs depict boxplots showing median (center line), upper and lower quartiles (box limits), minimum and maximum (whiskers) and outliers. Spearman’s rank correlation was used for correlations with *p*-values < 0.05 as statistically significant.

**Supplementary figure 6**

**Fig. S6 Immune profiling of eWAT during HFD-fed conditions in C57BL/6J mice.** (A) Concentrations of TNF-α, IL-33 and IL-5 in eWAT. IL-25, IL-13 and IL-4 were undetectable. Quantification by flow cytometry (as % of total CD45+ cells) of (B) total macrophages and indicated subsets, (C) eosinophils, (D) total monocytes and indicated subsets, (E) dendritic cells and indicated subsets, (F) CD8+ T-cells, CD4+ T-cells and indicated subsets, and (G) ILCs and indicated subsets in eWAT. N=10-12 per group, derived from three experiments. **p* < 0.05, ***p* < 0.01, ****p* < 0.001 by Mann-Whitney *U* test followed by correction for multiple comparisons using Benjamini-Hochberg method. Graphs depict boxplots showing median (center line), upper and lower quartiles (box limits), minimum and maximum (whiskers) and outliers. Spearman’s rank correlation was used for correlations with *p*-values < 0.05 as statistically significant.

**A**

**F**

**E**

**G**

**B**

**C**

**D**

**SUPPLEMENTARY TABLES**

**Supplementary table 1.** Composition of experimental diets.

| Diet | **HFD** | **RFD** |  | Diet | **HFD** | **RFD** |
| --- | --- | --- | --- | --- | --- | --- |
| Gross Energy (GE) | kJ% | kJ% |  | Fatty acids | [%] | [%] |
| Fat | 60 | 10 |  | C 12:0 | 0.07 | 0.01 |
| Protein | 20 | 20 |  | C 14:0 | 0.44 | 0.04 |
| Carbohydrates | 20 | 70 |  | C 16:0 | 7.93 | 0.69 |
| Crude Nutrients | [%] | [%] |  | C 18:0 | 4.37 | 0.31 |
| Crude protein (N x 6.25) | 24.4 | 18.2 |  | C 20:0 | 0.11 | 0.02 |
| Crude fat | 34.6 | 4.1 |  | C 16:1 | 0.94 | 0.05 |
| Crude fibre | 6 | 5 |  | C 18:1 | 13.97 | 1.3 |
| Crude ash | 5.3 | 5.3 |  | C 18:2 | 4.64 | 1.43 |
| Starch | 0.1 | 43.5 |  | C 18:3 | 0.49 | 0.15 |
| Sugar | 9.4 | 6.8 |  | Vitamins | per kg | per kg |
| N free extracts | 26.3 | 63 |  | Vitamin A | 15000 IU | 15000 IU |
| Minerals | [%] | [%] |  | Vitamin D3 | 1500 IU | 1500 IU |
| Calcium | 0.92 | 0.92 |  | Vitamin E | 150 mg | 150 mg |
| Phosphorus | 0.64 | 0.63 |  | Vitamin K (as MNB) | 20 mg | 20 mg |
| Ca/P | 1.44 | 1.46:1 |  | Thiamine (B1) | 25 mg | 25 mg |
| Sodium | 0.2 | 0.2 |  | Riboflavin (B2) | 16 mg | 16 mg |
| Magnesium | 0.23 | 0.23 |  | Pyridoxine (B6) | 16 mg | 16 mg |
| Potassium | 0.97 | 0.97 |  | Cobalamin (B12) | 30 μg | 31 μg |
| Amino acids | [%] | [%] |  | Nicotinic acid | 47 mg | 47 mg |
| Lysine | 2.02 | 1.52 |  | Pantothenic acid | 55 mg | 55 mg |
| Methionine | 0.86 | 0.66 |  | Folic acid | 16 mg | 16 mg |
| Cystine | 0.45 | 0.33 |  | Biotin | 300 μg | 301 μg |
| Met+Cys | 1.31 | 0.99 |  | Choline | 920 mg | 921 mg |
| Threonine | 1.07 | 0.8 |  | Trace elements | per kg | per kg |
| Tryptophan | 0.33 | 0.24 |  | Iron | 168 mg | 168 mg |
| Arginine | 0.95 | 0.71 |  | Manganese | 95 mg | 95 mg |
| Histidine | 0.74 | 0.55 |  | Zinc | 65 mg | 65 mg |
| Valine | 1.7 | 1.27 |  | Copper | 13mg | 13mg |
| Isoleucine | 1.38 | 1.02 |  | Iodine | 1.2 mg | 1.2 mg |
| Leucine | 2.42 | 1.8 |  | Selenium | 0.2 mg | 0.2 mg |
| Phenylalanine | 1.27 | 0.95 |  |  |  |  |
| Phe+Tyr | 2.56 | 1.9 |  |  |  |  |
| Glycine | 0.52 | 0.39 |  |  |  |  |
| Glycine | 5.5 | 4.1 |  |  |  |  |
| Aspartic acid | 1.82 | 1.35 |  |  |  |  |
| Proline | 2.8 | 2.09 |  |  |  |  |
| Serine | 1.46 | 1.09 |  |  |  |  |
| Alanine | 0.81 | 0.55 |  |  |  |  |

**Supplementary table 2.** Reagents and primers used for reverse transcription and cDNA amplification.

| **Reagents** | **Supplier** | **Cat/Ref No.** |
| --- | --- | --- |
| cDNA synthesis and amplification |  |  |
| SuperScript II Reverse transcription primer | IDT | NA |
| 5′-AAGCAGTGGTATCAACGCAGAGTACT30VN-3′ |  |  |
| dNTPs | Thermo Fisher | R0192 |
| TSO-primer | Exiqon | NA |
| 5′-AAGCAGTGGTATCAACGCAGAGTACATrGrG+G-3′ |  |  |
| SuperScript II reverse transcriptase | Invitrogen | 18064-071 |
| RNAse inhibitor | Clontech | 23013-A |
| Superscript II First-Strand Buffer | Invitrogen | 18064-071 |
| DTT | Invitrogen | 18064-014 |
| Betaine | Sigma-Aldrich | B0300 |
| MgCl_2_ | Sigma-Aldrich | M8266 |
|  |  |  |
| cDNA amplification |  |  |
| KAPA HotStart HIFI 2× ReadyMix | Roche | KK2602 |
| cDNA preamplification primer |  |  |
| 5′-AAGCAGTGGTATCAACGCAGAGT-3′ |  |  |
|  |  |  |
| cDNA purification |  |  |
| AMPure XP beads | Beckman Coulter | A63881 |

**Supplementary table 3.** Reagents and primers used for qPCR.

| **Reagents** | **Supplier** | **Cat./Ref No.** |
| --- | --- | --- |
| qPCR |  |  |
| TaqMan Fast Universal PCR Master Mix | Applied Biosystems | 4352042 |
| TaqMan Gene Expression Assay Mix | Applied Biosystems | 4369016 |
| *Gapdh* | IDT DNA | Mm.PT.39a.1 |
| Forward primer, 5' - GTG GAG TCA TAC TGG AAC ATG TAG |  |  |
| Reverse primer, 5' - AAT GGT GAA GGT CGG TGT G |  |  |
| *Dclk1* | IDT DNA | Mm.PT.58.21899992 |
| Forward primer, 5' - CCA TCA CAA ACC ATA CAC ATC G |  |  |
| Reverse primer, 5' - GCA AGT CAC CAA GTC CAT CA |  |  |
| *Il25* | IDT DNA | Mm.PT.58.28942186 |
| Forward primer, 5' - CGA TTC AAG TCC CTG TCC AA |  |  |
| Reverse primer, 5' - AAG TGG AGC TCT GCA TCT G |  |  |
| *Tslp* | IDT DNA | Mm.PT.58.41321689 |
| Forward primer, 5' - TTG TGC CAT TTC CTG AGT ACC |  |  |
| Reverse primer, 5' - TCT CAA TCC TAT CCC TGG CT |  |  |
| *Gp2* | IDT DNA | Mm.PT.56a.8772151 |
| Forward primer, 5' - GTC AAC TTC CAG TGT GCC TAC |  |  |
| Reverse primer, 5' - CTC CGT CCA CAT CAA CAG TC |  |  |

**Supplementary table 4.** List of Reactome pathways (related to figure 2)

P1: Metal sequestration by antimicrobial proteins

P2: Phase 1 − inactivation of fast Na+ channels

P3: TWIK−releated acid−sensitive K+ channel (TASK)

P4: Signaling by Insulin receptor

P5: Activated NTRK3 signals through PI3K

P6: Antimicrobial peptides

P7: IRS activation

P8: GABA A receptor activation

P9: Synthesis of PIPs in the nucleus

P10: The NLRP1 inflammasome

P11: Interleukin−18 signaling

P12: ARMS−mediated activation

P13: Metabolism of ingested SeMet, Sec, MeSec into H2Se

P14: DAP12 interactions

P15: RUNX3 regulates YAP1−mediated transcription

P16: Endosomal/Vacuolar pathway

P17: Reversal of alkylation damage by DNA dioxygenases

P18: Interaction with The Zona Pellucida

P19: NGF processing

P20: ER−Phagosome pathway

P21: Phase 4 − resting membrane potential

P22: GABA B receptor activation

P23: c−src mediated regulation of Cx43 function and closure of gap junctions

P24: Phospholipase C−mediated cascade; FGFR2

P25: Nucleobase catabolism

P26: Activation of the mRNA upon binding of the cap−binding complex and eIFs, and subsequent binding to 43S

P27: Assembly of the pre−replicative complex

P28: Microtubule−dependent trafficking of connexons from Golgi to the plasma membrane

P29: Pre−NOTCH Processing in Golgi

P30: Advanced glycosylation endproduct receptor signaling

P31: Pyrophosphate hydrolysis

P32: Glucuronidation

P33: Scavenging by Class B Receptors

P34: Chemokine receptors bind chemokines

P35: Acyl chain remodeling of CL

P36: Abacavir transmembrane transport

P37: NADPH regeneration

P38: Beta oxidation of lauroyl−CoA to decanoyl−CoA−CoA

P39: Cytosolic tRNA aminoacylation

P40: Wax biosynthesis

P41: Synthesis of Ketone Bodies

P42: Sulfur amino acid metabolism

P43: Mitotic Telophase/Cytokinesis

P44: Glycine degradation

P45: Triglyceride catabolism

P46: TET1,2,3 and TDG demethylate DNA

P47: Beta oxidation of myristoyl−CoA to lauroyl−CoA

P48: Telomere Extension By Telomerase

P49: Sensing of DNA Double Strand Breaks

P50: Downstream signaling of activated FGFR1

P51: Synthesis of (16−20)−hydroxyeicosatetraenoic acids (HETE)
