# Supplementary material for: Small Intestinal Tuft Cell Activity Associates With Energy Metabolism in Diet-Induced Obesity
Source: Front Immunol. 2021 May 28;12:629391. doi: 10.3389/fimmu.2021.629391 (PMC8195285; doi:10.3389/fimmu.2021.629391)
Supplement: Supplementary file 2 [file DataSheet_2.pdf]

Supplementary data 1: List of differently regulated genes in small intestinal tuft cells under HFD-feeding vs RFD-feeding conditions for 9 weeks.

GeneID	GeneName	GeneSymbol	HFD/RFD 9wk_fold change	*q-value*_ HFD/RFD_9wk
ENSMUSG00000094362	defensin, alpha, 33	Defa33	12376100490	0.02628
ENSMUSG00000066072	cytochrome P450, family 4, subfamily a, polypeptide 10	Cyp4a10	1616259515	0.01673
ENSMUSG00000029762	aldo-keto reductase family 1, member B8	Akr1b8	901140147.7	0.06232
ENSMUSG00000072949	acyl-CoA thioesterase 1	Acot1	48.209	0.01673
ENSMUSG00000069309	histone cluster 1, H2an	Hist1h2an	29.806	0.0324
ENSMUSG00000038482	transcription factor Dp 1	Tfdp1	14.892	0.01673
ENSMUSG00000027875	3-hydroxy-3-methylglutaryl-Coenzyme A synthase 2	Hmgcs2	14.681	0
ENSMUSG00000067150	exportin 5	Xpo5	14.354	0.07841
ENSMUSG00000054630	UDP glucuronosyltransferase 2 family, polypeptide B5	Ugt2b5	13.264	0.01673
ENSMUSG00000105987	expressed sequence AI506816	AI506816	11.704	0
ENSMUSG00000012640	zinc finger protein 715	Zfp715	8.434	0.02933
ENSMUSG00000042834	neuronal regeneration related protein	Nrep	7.194	0.01875
ENSMUSG00000051379	fibronectin leucine rich transmembrane protein 3	Flrt3	6.425	0.05219
ENSMUSG00000022322	Shc SH2-domain binding protein 1	Shcbp1	6.092	0.05284
ENSMUSG00000049396	gem nuclear organelle associated protein 4	Gemin4	6.01	0.0324
ENSMUSG00000008450	nuclear transport factor 2	Nutf2	5.401	0.06678
ENSMUSG00000019988	neural precursor cell expressed, developmentally down-regulated gene 1	Nedd1	5.189	0.0497
ENSMUSG00000086513	RIKEN cDNA 9130208D14 gene	9130208D14Rik	4.921	0.01228
ENSMUSG00000048007	translocase of inner mitochondrial membrane 8A1	Timm8a1	4.9	0.01779
ENSMUSG00000011752	phosphoglycerate mutase 1	Pgam1	4.723	0.0324
ENSMUSG00000081058	histone cluster 2, H3c2	Hist2h3c2	4.576	0.05656
ENSMUSG00000045996	polymerase (RNA) II (DNA directed) polypeptide K	Polr2k	4.34	0.05219
ENSMUSG00000017057	interleukin 13 receptor, alpha 1	Il13ra1	4.267	0.05997
ENSMUSG00000020319	WD repeat containing planar cell polarity effector	Wdpcp	4.231	0.01228
ENSMUSG00000022031	elongator acetyltransferase complex subunit 3	Elp3	4.231	0.05834
ENSMUSG00000095649	predicted gene 8979	Gm8979	3.985	0.00769
ENSMUSG00000018362	karyopherin (importin) alpha 2	Kpna2	3.943	0.01779
ENSMUSG00000090124	UDP glucuronosyltransferase 1 family, polypeptide A7C	Ugt1a7c	3.925	0.01228
ENSMUSG00000021238	aldehyde dehydrogenase family 6, subfamily A1	Aldh6a1	3.727	0.0223
ENSMUSG00000021131	ERH mRNA splicing and mitosis factor	Erh	3.584	0
ENSMUSG00000040728	epithelial splicing regulatory protein 1	Esrp1	3.454	0.00769
ENSMUSG00000073676	heat shock protein 1 (chaperonin 10)	Hspe1	3.406	0
ENSMUSG00000008540	microsomal glutathione S-transferase 1	Mgst1	3.396	0
ENSMUSG00000042271	nuclear transport factor 2-like export factor 2	Nxt2	3.363	0.01779
ENSMUSG00000001630	serine/threonine kinase 38 like	Stk38l	3.174	0.01673
ENSMUSG00000025739	guanine nucleotide binding protein (G protein), gamma 13	Gng13	3.115	0.01875
ENSMUSG00000064289	TRAF family member-associated Nf-kappa B activator	Tank	3.098	0.06168
ENSMUSG00000005481	DEAD (Asp-Glu-Ala-Asp) box polypeptide 39	Ddx39	2.991	0.00769
ENSMUSG00000030303	fatty acyl CoA reductase 2	Far2	2.956	0.01228
ENSMUSG00000034674	thymine DNA glycosylase	Tdg	2.948	0.05736
ENSMUSG00000025240	SAC1 suppressor of actin mutations 1-like (yeast)	Sacm1l	2.942	0.02933
ENSMUSG00000028495	ribosomal protein S6	Rps6	2.831	0
ENSMUSG00000022336	eukaryotic translation initiation factor 3, subunit E	Eif3e	2.82	0.01228
ENSMUSG00000023110	protein arginine N-methyltransferase 5	Prmt5	2.776	0.02516
ENSMUSG00000030793	PYD and CARD domain containing	Pycard	2.772	0.0223
ENSMUSG00000025980	heat shock protein 1 (chaperonin)	Hspd1	2.77	0.01779
ENSMUSG00000061477	ribosomal protein S7	Rps7	2.767	0
ENSMUSG00000054717	high mobility group box 2	Hmgb2	2.757	0
ENSMUSG00000022026	olfactomedin 4	Olfm4	2.755	0.01875
ENSMUSG00000020290	exportin 1	Xpo1	2.723	0.0223
ENSMUSG00000071866	peptidylprolyl isomerase A	Ppia	2.72	0.01523
ENSMUSG00000027195	hydroxysteroid (17-beta) dehydrogenase 12	Hsd17b12	2.713	0.01779
ENSMUSG00000030942	THUMP domain containing 1	Thumpd1	2.702	0.02681
ENSMUSG00000042590	importin 11	Ipo11	2.701	0.05656
ENSMUSG00000002550	uridine-cytidine kinase 1	Uck1	2.673	0.01779
ENSMUSG00000019373	COP9 signalosome subunit 3	Cops3	2.636	0.01875
ENSMUSG00000030357	FK506 binding protein 4	Fkbp4	2.624	0
ENSMUSG00000003779	kinesin family member 20A	Kif20a	2.622	0.05203
ENSMUSG00000059447	hydroxyacyl-Coenzyme A dehydrogenase/3-ketoacyl-Coenzyme A thiolase/enoyl-Co	Hadhb	2.612	0
ENSMUSG00000068039	t-complex protein 1	Tcp1	2.611	0.00769
ENSMUSG00000018740	solute carrier family 25, member 35	Slc25a35	2.592	0.0717
ENSMUSG00000079435	ribosomal protein L36A	Rpl36a	2.591	0
ENSMUSG00000059796	eukaryotic translation initiation factor 4A1	Eif4a1	2.525	0
ENSMUSG00000023057	fatty acid binding protein 2, intestinal	Fabp2	2.497	0.02628
ENSMUSG00000035754	WD repeat domain 18	Wdr18	2.468	0.00769
ENSMUSG00000021832	proteasome (prosome, macropain) 26S subunit, ATPase, 6	Psmc6	2.467	0.01673
ENSMUSG00000026003	acyl-Coenzyme A dehydrogenase, long-chain	Acadl	2.463	0
ENSMUSG00000010205	ribonucleoprotein, PTB-binding 1	Raver1	2.451	0.01779
ENSMUSG00000074656	eukaryotic translation initiation factor 2, subunit 2 (beta)	Eif2s2	2.436	0.02516
ENSMUSG00000026360	regulator of G-protein signaling 2	Rgs2	2.432	0.02516
ENSMUSG00000005732	RAN binding protein 1	Ranbp1	2.419	0.01673
ENSMUSG00000020078	VPS26 retromer complex component A	Vps26a	2.404	0.01875
ENSMUSG00000020911	keratin 19	Krt19	2.393	0.01673
ENSMUSG00000057113	nucleophosmin 1	Npm1	2.391	0.00769
ENSMUSG00000029447	chaperonin containing Tcp1, subunit 6a (zeta)	Cct6a	2.387	0.00769
ENSMUSG00000015120	ubiquitin-conjugating enzyme E2I	Ube2i	2.377	0.02516
ENSMUSG00000028494	perilipin 2	Plin2	2.37	0.00769
ENSMUSG00000028409	smu-1 suppressor of mec-8 and unc-52 homolog (C. elegans)	Smu1	2.356	0.01779
ENSMUSG00000074781	ubiquitin-conjugating enzyme E2N	Ube2n	2.352	0.0223
ENSMUSG00000066551	high mobility group box 1	Hmgb1	2.346	0
ENSMUSG00000037894	H2A histone family, member Z	H2afz	2.344	0.05219
ENSMUSG00000022403	suppression of tumorigenicity 13	St13	2.341	0
ENSMUSG00000027834	serine (or cysteine) peptidase inhibitor, clade I, member 1	Serpini1	2.341	0
ENSMUSG00000030105	ADP-ribosylation factor-like 8B	Arl8b	2.337	0.01779

ENSMUSG00000021546	heterogeneous nuclear ribonucleoprotein K	Hnrnpk	2.336	0.01779
ENSMUSG00000032116	STT3, subunit of the oligosaccharyltransferase complex, homolog A (S. cerevisiae)	Stt3a	2.336	0.02933
ENSMUSG00000049517	ribosomal protein S23	Rps23	2.326	0
ENSMUSG00000072235	tubulin, alpha 1A	Tuba1a	2.303	0.02516
ENSMUSG00000036257	patatin-like phospholipase domain containing 8	Pnpla8	2.283	0
ENSMUSG00000058558	ribosomal protein L5	Rpl5	2.278	0.01875
ENSMUSG00000068882	Sjogren syndrome antigen B	Ssb	2.266	0
ENSMUSG00000032279	isocitrate dehydrogenase 3 (NAD+) alpha	Idh3a	2.231	0.02516
ENSMUSG00000020460	ribosomal protein S27A	Rps27a	2.23	0.00769
ENSMUSG00000054408	signal peptidase complex subunit 3 homolog (S. cerevisiae)	Spcs3	2.215	0.0324
ENSMUSG00000090035	polypeptide N-acetylgalactosaminyltransferase 4	Galnt4	2.214	0.0223
ENSMUSG00000032314	electron transferring flavoprotein, alpha polypeptide	Etfa	2.211	0.01673
ENSMUSG00000031246	SH3-binding domain glutamic acid-rich protein like	Sh3bgrl	2.209	0.05111
ENSMUSG00000060073	proteasome (prosome, macropain) subunit, alpha type 3	Psma3	2.2	0.00769
ENSMUSG00000016921	serine/arginine-rich splicing factor 6	Srsf6	2.199	0
ENSMUSG00000045427	heterogeneous nuclear ribonucleoprotein H2	Hnrnph2	2.198	0.05656
ENSMUSG00000026926	peptidase (mitochondrial processing) alpha	Pmpca	2.192	0.01779
ENSMUSG00000031980	angiotensinogen (serpin peptidase inhibitor, clade A, member 8)	Agt	2.192	0.02681
ENSMUSG00000027193	apoptosis inhibitor 5	Api5	2.178	0.0324
ENSMUSG00000028607	carnitine palmitoyltransferase 2	Cpt2	2.175	0.01875
ENSMUSG00000040681	high mobility group nucleosomal binding domain 1	Hmgn1	2.174	0.02806
ENSMUSG00000016534	lysosomal-associated membrane protein 2	Lamp2	2.173	0.05656
ENSMUSG00000006728	cyclin-dependent kinase 4	Cdk4	2.172	0.00769
ENSMUSG00000058546	ribosomal protein L23A	Rpl23a	2.165	0.00769
ENSMUSG00000061787	ribosomal protein S17	Rps17	2.161	0.00769
ENSMUSG00000014226	calcyclin binding protein	Cacybp	2.16	0.02933
ENSMUSG00000031634	UFM1-specific peptidase 2	Ufsp2	2.155	0.02681
ENSMUSG00000078941	adenylate kinase 6	Ak6	2.152	0.00769
ENSMUSG00000026489	coenzyme Q8A	Coq8a	2.148	0.01779
ENSMUSG00000058258	isopentenyl-diphosphate delta isomerase	Idi1	2.144	0.01673
ENSMUSG00000057421	LAS1-like (S. cerevisiae)	Las1l	2.137	0.00769
ENSMUSG00000047213	YTH N6-methyladenosine RNA binding protein 3	Ythdf3	2.122	0.06385
ENSMUSG00000028622	mitochondrial ribosomal protein L37	Mrpl37	2.117	0.02516
ENSMUSG00000022205	SUB1 homolog (S. cerevisiae)	Sub1	2.113	0.0223
ENSMUSG00000020664	dihydrolipoamide dehydrogenase	Dld	2.112	0.05992
ENSMUSG00000029634	ring finger protein (C3H2C3 type) 6	Rnf6	2.098	0.01779
ENSMUSG00000021660	basic transcription factor 3	Btf3	2.088	0
ENSMUSG00000071172	serine/arginine-rich splicing factor 3	Srsf3	2.088	0.0324
ENSMUSG00000029614	ribosomal protein L6	Rpl6	2.082	0
ENSMUSG00000025156	G protein pathway suppressor 1	Gps1	2.081	0.02516
ENSMUSG00000063316	ribosomal protein L27	Rpl27	2.073	0
ENSMUSG00000028675	proline-rich nuclear receptor coactivator 2	Pnrc2	2.07	0.04476
ENSMUSG00000037601	NME/NM23 nucleoside diphosphate kinase 1	Nme1	2.068	0.00769
ENSMUSG00000020720	proteasome (prosome, macropain) 26S subunit, non-ATPase, 12	Psmd12	2.055	0.01779
ENSMUSG00000012405	ribosomal protein L15	Rpl15	2.033	0
ENSMUSG00000025950	isocitrate dehydrogenase 1 (NADP+), soluble	Idh1	2.032	0
ENSMUSG00000020929	elongation factor Tu GTP binding domain containing 2	Eftud2	2.032	0.00769
ENSMUSG00000043716	ribosomal protein L7	Rpl7	2.031	0
ENSMUSG00000062328	ribosomal protein L17	Rpl17	2.012	0
ENSMUSG00000013701	translocase of inner mitochondrial membrane 23	Timm23	2.002	0.0223
ENSMUSG00000047866	lon peptidase 2, peroxisomal	Lonp2	2.001	0.05992
ENSMUSG00000024997	peroxiredoxin 3	Prdx3	1.983	0.02516
ENSMUSG00000024097	serine/arginine-rich splicing factor 7	Srsf7	1.979	0.00769
ENSMUSG00000060373	heterogeneous nuclear ribonucleoprotein C	Hnrnpc	1.977	0.06111
ENSMUSG00000091896	ubiquitin-conjugating enzyme E2D 2A	Ube2d2a	1.97	0.05665
ENSMUSG00000030754	coatomer protein complex, subunit beta 1	Copb1	1.969	0.01228
ENSMUSG00000020946	golgi SNAP receptor complex member 2	Gosr2	1.968	0.01228
ENSMUSG00000032959	phosphatidylethanolamine binding protein 1	Pebp1	1.963	0
ENSMUSG00000007458	mannose-6-phosphate receptor, cation dependent	M6pr	1.961	0
ENSMUSG00000005656	sorting nexin 6	Snx6	1.961	0.02628
ENSMUSG00000046434	heterogeneous nuclear ribonucleoprotein A1	Hnrnpa1	1.953	0.0223
ENSMUSG00000000171	succinate dehydrogenase complex, subunit D, integral membrane protein	Sdhd	1.951	0.05616
ENSMUSG00000027597	S-adenosylhomocysteine hydrolase	Ahcy	1.95	0
ENSMUSG00000031320	ribosomal protein S4, X-linked	Rps4x	1.95	0.0223
ENSMUSG00000041453	ribosomal protein L21	Rpl21	1.943	0
ENSMUSG00000030403	vasodilator-stimulated phosphoprotein	Vasp	1.938	0.02806
ENSMUSG00000027405	NOP56 ribonucleoprotein	Nop56	1.923	0.02516
ENSMUSG00000023004	tubulin, alpha 1B	Tuba1b	1.922	0
ENSMUSG00000025979	MOB family member 4, phocein	Mob4	1.921	0.00769
ENSMUSG00000028081	ribosomal protein S3A1	Rps3a1	1.92	0
ENSMUSG00000090862	ribosomal protein S13	Rps13	1.915	0
ENSMUSG00000028156	eukaryotic translation initiation factor 4E	Eif4e	1.912	0.02933
ENSMUSG00000024165	Jupiter microtubule associated homolog 2	Jpt2	1.912	0.0324
ENSMUSG00000063856	glutathione peroxidase 1	Gpx1	1.909	0.01779
ENSMUSG00000047675	ribosomal protein S8	Rps8	1.905	0
ENSMUSG00000025745	hydroxyacyl-Coenzyme A dehydrogenase/3-ketoacyl-Coenzyme A thiolase/enoyl-Co	Hadha	1.9	0
ENSMUSG00000006057	ATP synthase, H+ transporting, mitochondrial F0 complex, subunit C1 (subunit 9)	Atp5g1	1.895	0.05656
ENSMUSG00000062647	ribosomal protein L7A	Rpl7a	1.891	0
ENSMUSG00000030751	proteasome (prosome, macropain) subunit, alpha type 1	Psma1	1.889	0.0223
ENSMUSG00000062867	inosine monophosphate dehydrogenase 2	Impdh2	1.883	0.01875
ENSMUSG00000028405	aconitase 1	Aco1	1.877	0.0324
ENSMUSG00000029430	RAN, member RAS oncogene family	Ran	1.875	0.00769
ENSMUSG00000025613	chaperonin containing Tcp1, subunit 8 (theta)	Cct8	1.875	0.01228
ENSMUSG00000026238	prothymosin alpha	Ptma	1.874	0
ENSMUSG00000006998	proteasome (prosome, macropain) 26S subunit, non-ATPase, 2	Psmd2	1.864	0.05656
ENSMUSG00000060743	H3 histone, family 3A	H3f3a	1.861	0.01228
ENSMUSG00000060036	ribosomal protein L3	Rpl3	1.86	0

ENSMUSG00000023832	acetyl-Coenzyme A acetyltransferase 2	Acat2	1.858	0.0717
ENSMUSG00000022010	TSC22 domain family, member 1	Tsc22d1	1.851	0.06868
ENSMUSG00000008682	ribosomal protein L10	Rpl10	1.838	0.01228
ENSMUSG00000028691	peroxiredoxin 1	Prdx1	1.831	0.01779
ENSMUSG00000087260	late endosomal/lysosomal adaptor, MAPK and MTOR activator 5	Lamtor5	1.83	0.01228
ENSMUSG00000073639	RAB18, member RAS oncogene family	Rab18	1.829	0.02516
ENSMUSG00000032399	ribosomal protein L4	Rpl4	1.826	0
ENSMUSG00000062981	mitochondrial ribosomal protein L42	Mrpl42	1.824	0.01228
ENSMUSG00000021417	enoyl-Coenzyme A delta isomerase 2	Eci2	1.823	0
ENSMUSG00000008668	ribosomal protein S18	Rps18	1.819	0
ENSMUSG00000020089	pyrophosphatase (inorganic) 1	Ppa1	1.816	0.01228
ENSMUSG00000061666	glycerophosphodiester phosphodiesterase domain containing 1	Gdpd1	1.811	0.01228
ENSMUSG00000005610	eukaryotic translation initiation factor 4, gamma 2	Eif4g2	1.811	0.01779
ENSMUSG00000053332	growth arrest specific 5	Gas5	1.804	0.02516
ENSMUSG00000004264	prohibitin 2	Phb2	1.791	0
ENSMUSG00000018583	GTPase activating protein (SH3 domain) binding protein 1	G3bp1	1.777	0.05219
ENSMUSG00000036752	tubulin, beta 4B class IVB	Tubb4b	1.774	0
ENSMUSG00000030884	ubiquinol cytochrome c reductase core protein 2	Uqcrc2	1.77	0.02681
ENSMUSG00000024900	carnitine palmitoyltransferase 1a, liver	Cpt1a	1.768	0
ENSMUSG00000028837	proteasome (prosome, macropain) subunit, beta type 2	Psmb2	1.767	0.00769
ENSMUSG00000076432	tyrosine 3-monooxygenase/tryptophan 5-monooxygenase activation protein theta	Ywhaq	1.765	0.01228
ENSMUSG00000015733	capping protein (actin filament) muscle Z-line, alpha 2	Capza2	1.765	0.0223
ENSMUSG00000024583	thioredoxin-like 1	Txnl1	1.748	0
ENSMUSG00000030869	NADH:ubiquinone oxidoreductase subunit AB1	Ndufab1	1.748	0.06678
ENSMUSG00000098274	ribosomal protein L24	Rpl24	1.743	0.01673
ENSMUSG00000027282	mitochondrial carrier 2	Mtch2	1.74	0.02933
ENSMUSG00000003038	high mobility group nucleosomal binding domain 2	Hmgn2	1.736	0.0223
ENSMUSG00000061838	succinate-Coenzyme A ligase, GDP-forming, beta subunit	Suclg2	1.73	0.00769
ENSMUSG00000034892	ribosomal protein S29	Rps29	1.728	0
ENSMUSG00000020372	receptor for activated C kinase 1	Rack1	1.726	0.02806
ENSMUSG00000037805	ribosomal protein L10A	Rpl10a	1.72	0
ENSMUSG00000022744	claudin domain containing 1	Cldnd1	1.717	0.02516
ENSMUSG00000055302	Morf4 family associated protein 1	Mrfap1	1.711	0.0223
ENSMUSG00000031696	VPS35 retromer complex component	Vps35	1.703	0.01228
ENSMUSG00000047215	ribosomal protein L9	Rpl9	1.695	0
ENSMUSG00000057841	ribosomal protein L32	Rpl32	1.694	0
ENSMUSG00000020361	heat shock protein 4	Hspa4	1.69	0.00769
ENSMUSG00000002741	YKT6 v-SNARE homolog (S. cerevisiae)	Ykt6	1.683	0.0223
ENSMUSG00000020267	histidine triad nucleotide binding protein 1	Hint1	1.683	0.04376
ENSMUSG00000049775	thymosin, beta 4, X chromosome	Tmsb4x	1.682	0.05665
ENSMUSG00000028851	nudC nuclear distribution protein	Nudc	1.676	0.01228
ENSMUSG00000026229	proteasome (prosome, macropain) 26S subunit, non-ATPase, 1	Psmd1	1.676	0.05203
ENSMUSG00000014077	calcineurin-like EF hand protein 1	Chp1	1.673	0.02628
ENSMUSG00000028452	valosin containing protein	Vcp	1.668	0.0223
ENSMUSG00000020849	tyrosine 3-monooxygenase/tryptophan 5-monooxygenase activation protein, epsilor	Ywhae	1.665	0.01875
ENSMUSG00000030057	cellular nucleic acid binding protein	Cnbp	1.656	0.02681
ENSMUSG00000059291	ribosomal protein L11	Rpl11	1.655	0
ENSMUSG00000000563	ATP synthase, H+ transporting, mitochondrial F0 complex, subunit B1	Atp5f1	1.654	0.02933
ENSMUSG00000009927	ribosomal protein S25	Rps25	1.647	0
ENSMUSG00000021218	guanosine diphosphate (GDP) dissociation inhibitor 2	Gdi2	1.645	0.02516
ENSMUSG00000078812	eukaryotic translation initiation factor 5A	Eif5a	1.642	0
ENSMUSG00000068749	proteasome (prosome, macropain) subunit, alpha type 5	Psma5	1.641	0.02681
ENSMUSG00000067274	ribosomal protein, large, P0	Rplp0	1.639	0
ENSMUSG00000027170	eukaryotic translation initiation factor 3, subunit M	Eif3m	1.637	0.0223
ENSMUSG00000062006	ribosomal protein L34	Rpl34	1.636	0
ENSMUSG00000009079	Ewing sarcoma breakpoint region 1	Ewsr1	1.626	0.01673
ENSMUSG00000005161	peroxiredoxin 2	Prdx2	1.626	0.02933
ENSMUSG00000037742	eukaryotic translation elongation factor 1 alpha 1	Eef1a1	1.623	0
ENSMUSG00000025290	ribosomal protein S24	Rps24	1.621	0
ENSMUSG00000025351	CD63 antigen	Cd63	1.615	0.02933
ENSMUSG00000020149	RAB1A, member RAS oncogene family	Rab1a	1.614	0.01779
ENSMUSG00000015671	proteasome (prosome, macropain) subunit, alpha type 2	Psma2	1.612	0.01673
ENSMUSG00000070372	capping protein (actin filament) muscle Z-line, alpha 1	Capza1	1.611	0
ENSMUSG00000000740	ribosomal protein L13	Rpl13	1.61	0
ENSMUSG00000025544	transmembrane 9 superfamily member 2	Tm9sf2	1.61	0.06111
ENSMUSG00000024370	CDC23 cell division cycle 23	Cdc23	1.601	0.05738
ENSMUSG00000023944	heat shock protein 90 alpha (cytosolic), class B member 1	Hsp90ab1	1.6	0.0717
ENSMUSG00000003623	carnitine O-octanoyltransferase	Crot	1.591	0.01228
ENSMUSG00000006498	polypyrimidine tract binding protein 1	Ptbp1	1.574	0.01228
ENSMUSG00000029632	Ndufa4, mitochondrial complex associated	Ndufa4	1.574	0.01673
ENSMUSG00000037563	ribosomal protein S16	Rps16	1.572	0
ENSMUSG00000022477	aconitase 2, mitochondrial	Aco2	1.571	0
ENSMUSG00000014195	DnaJ heat shock protein family (Hsp40) member C7	Dnajc7	1.566	0.06718
ENSMUSG00000093904	translocase of outer mitochondrial membrane 20	Tomm20	1.565	0.02628
ENSMUSG00000020402	voltage-dependent anion channel 1	Vdac1	1.562	0
ENSMUSG00000073702	ribosomal protein L31	Rpl31	1.558	0.01228
ENSMUSG00000030654	ADP-ribosylation factor-like 6 interacting protein 1	Arl6ip1	1.558	0.02516
ENSMUSG00000032324	tetraspanin 3	Tspan3	1.554	0.06168
ENSMUSG00000040952	ribosomal protein S19	Rps19	1.548	0.00769
ENSMUSG00000022858	transformer 2 beta	Tra2b	1.543	0.01673
ENSMUSG00000020048	heat shock protein 90, beta (Grp94), member 1	Hsp90b1	1.541	0.05992
ENSMUSG00000037072	selenoprotein F	Selenof	1.54	0.0324
ENSMUSG00000025794	ribosomal protein L14	Rpl14	1.536	0.04652
ENSMUSG00000035561	aldehyde dehydrogenase 1 family, member B1	Aldh1b1	1.533	0.00769
ENSMUSG00000026234	nucleolin	Ncl	1.532	0.01228
ENSMUSG00000038845	prohibitin	Phb	1.528	0.06232
ENSMUSG00000053898	enoyl coenzyme A hydratase 1, peroxisomal	Ech1	1.521	0.01673

ENSMUSG00000024740	damage specific DNA binding protein 1	Ddb1	1.52	0.04476
ENSMUSG00000045128	ribosomal protein L18A	Rpl18a	1.519	0.00769
ENSMUSG00000071644	eukaryotic translation elongation factor 1 gamma	Eef1g	1.515	0.00769
ENSMUSG00000022312	eukaryotic translation initiation factor 3, subunit H	Eif3h	1.512	0.01228
ENSMUSG00000052738	succinate-CoA ligase, GDP-forming, alpha subunit	Suclg1	1.512	0.05219
ENSMUSG00000039865	solute carrier family 44, member 3	Slc44a3	1.511	0.0717
ENSMUSG00000032096	archain 1	Arcn1	1.506	0.02681
ENSMUSG00000027406	isocitrate dehydrogenase 3 (NAD+) beta	Idh3b	1.505	0
ENSMUSG00000044573	acid phosphatase 1, soluble	Acp1	1.505	0.02516
ENSMUSG00000004980	heterogeneous nuclear ribonucleoprotein A2/B1	Hnrnpa2b1	1.502	0
ENSMUSG00000042079	heterogeneous nuclear ribonucleoprotein F	Hnrnpf	1.5	0.05465
ENSMUSG00000021520	ubiquinol-cytochrome c reductase binding protein	Uqcrb	1.493	0.02516
ENSMUSG00000046364	ribosomal protein L27A	Rpl27a	1.49	0.00769
ENSMUSG00000036309	S-phase kinase-associated protein 1A	Skp1a	1.488	0.0223
ENSMUSG00000021065	fucosyltransferase 8	Fut8	1.487	0.01875
ENSMUSG00000003429	ribosomal protein S11	Rps11	1.485	0.01875
ENSMUSG00000024608	ribosomal protein S14	Rps14	1.484	0
ENSMUSG00000021917	signal peptidase complex subunit 1 homolog (S. cerevisiae)	Spcs1	1.478	0.01228
ENSMUSG00000058600	ribosomal protein L30	Rpl30	1.477	0.05219
ENSMUSG00000026341	ARP3 actin-related protein 3	Actr3	1.466	0
ENSMUSG00000033047	eukaryotic translation initiation factor 3, subunit L	Eif3l	1.465	0.02394
ENSMUSG00000036371	serpine1 mRNA binding protein 1	Serbp1	1.46	0.01228
ENSMUSG00000028367	thioredoxin 1	Txn1	1.459	0.01673
ENSMUSG00000029198	GrpE-like 1, mitochondrial	Grpel1	1.458	0.06168
ENSMUSG00000012848	ribosomal protein S5	Rps5	1.457	0.00769
ENSMUSG00000007739	chaperonin containing Tcp1, subunit 4 (delta)	Cct4	1.451	0.00769
ENSMUSG00000032042	signal recognition particle receptor ('docking protein')	Srpr	1.45	0.01779
ENSMUSG00000025393	ATP synthase, H+ transporting mitochondrial F1 complex, beta subunit	Atp5b	1.449	0.01779
ENSMUSG00000029686	cullin 1	Cul1	1.447	0.01779
ENSMUSG00000032383	peptidylprolyl isomerase B	Ppib	1.446	0
ENSMUSG00000049091	selenophosphate synthetase 2	Sephs2	1.443	0.01875
ENSMUSG00000074129	ribosomal protein L13A	Rpl13a	1.438	0
ENSMUSG00000000399	NADH:ubiquinone oxidoreductase subunit A9	Ndufa9	1.435	0.07864
ENSMUSG00000015889	leukotriene A4 hydrolase	Lta4h	1.422	0.02933
ENSMUSG00000025362	ribosomal protein S26	Rps26	1.42	0
ENSMUSG00000002010	isocitrate dehydrogenase 3 (NAD+), gamma	Idh3g	1.42	0.0223
ENSMUSG00000022536	glyoxylate reductase 1 homolog (Arabidopsis)	Glyr1	1.42	0.0223
ENSMUSG00000022437	SAMM50 sorting and assembly machinery component	Samm50	1.419	0.02516
ENSMUSG00000048758	ribosomal protein L29	Rpl29	1.415	0.0223
ENSMUSG00000030007	chaperonin containing Tcp1, subunit 7 (eta)	Cct7	1.415	0.02394
ENSMUSG00000030868	dynactin 5	Dctn5	1.413	0.06948
ENSMUSG00000031701	DnaJ heat shock protein family (Hsp40) member A2	Dnaja2	1.408	0.06111
ENSMUSG00000031438	ring finger protein 128	Rnf128	1.407	0.0223
ENSMUSG00000009549	signal recognition particle 14	Srp14	1.407	0.06385
ENSMUSG00000025968	NADH:ubiquinone oxidoreductase core subunit S1	Ndufs1	1.405	0.09538
ENSMUSG00000006699	cell division cycle 42	Cdc42	1.399	0.01673
ENSMUSG00000060126	tumor protein, translationally-controlled 1	Tpt1	1.382	0.01673
ENSMUSG00000068823	cold shock domain containing E1, RNA binding	Csde1	1.381	0.05997
ENSMUSG00000059119	nucleosome assembly protein 1-like 4	Nap1l4	1.379	0.05616
ENSMUSG00000027882	syntaxin binding protein 3	Stxbp3	1.376	0.06678
ENSMUSG00000007815	ras homolog family member A	Rhoa	1.369	0.01228
ENSMUSG00000048076	ADP-ribosylation factor 1	Arf1	1.369	0.02516
ENSMUSG00000060938	ribosomal protein L26	Rpl26	1.367	0.05736
ENSMUSG00000000088	cytochrome c oxidase subunit 5A	Cox5a	1.366	0.06948
ENSMUSG00000030744	ribosomal protein S3	Rps3	1.356	0
ENSMUSG00000002102	proteasome (prosome, macropain) 26S subunit, ATPase 3	Psmc3	1.355	0.05834
ENSMUSG00000062825	actin, gamma, cytoplasmic 1	Actg1	1.354	0.01779
ENSMUSG00000044600	small integral membrane protein 7	Smim7	1.353	0.05992
ENSMUSG00000090733	ribosomal protein S27	Rps27	1.352	0.01673
ENSMUSG00000027900	DNA-damage regulated autophagy modulator 2	Dram2	1.35	0.0223
ENSMUSG00000036781	ribosomal protein S27-like	Rps27l	1.345	0.05997
ENSMUSG00000019370	calmodulin 3	Calm3	1.34	0.05656
ENSMUSG00000003309	adaptor protein complex AP-1, mu 2 subunit	Ap1m2	1.34	0.05997
ENSMUSG00000023456	triosephosphate isomerase 1	Tpi1	1.337	0.01779
ENSMUSG00000017801	MAX-like protein X	Mlx	1.33	0.0324
ENSMUSG00000056201	cofilin 1, non-muscle	Cfl1	1.326	0.01673
ENSMUSG00000022283	poly(A) binding protein, cytoplasmic 1	Pabpc1	1.323	0.01779
ENSMUSG00000022747	ST3 beta-galactoside alpha-2,3-sialyltransferase 6	St3gal6	1.321	0.02516
ENSMUSG00000040025	YTH N6-methyladenosine RNA binding protein 2	Ythdf2	1.319	0.05736
ENSMUSG00000037149	DEAD (Asp-Glu-Ala-Asp) box polypeptide 1	Ddx1	1.308	0.02628
ENSMUSG00000030432	ribosomal protein L28	Rpl28	1.307	0.0223
ENSMUSG00000070493	coiled-coil-helix-coiled-coil-helix domain containing 2	Chchd2	1.3	0.00769
ENSMUSG00000018286	proteasome (prosome, macropain) subunit, beta type 6	Psmb6	1.29	0.02628
ENSMUSG00000026021	small ubiquitin-like modifier 1	Sumo1	1.284	0.07864
ENSMUSG00000019494	COP9 signalosome subunit 6	Cops6	1.282	0.02681
ENSMUSG00000020738	small ubiquitin-like modifier 2	Sumo2	1.271	0
ENSMUSG00000006333	ribosomal protein S9	Rps9	1.265	0.01673
ENSMUSG00000062070	phosphoglycerate kinase 1	Pgk1	1.253	0.01673
ENSMUSG00000017404	ribosomal protein L19	Rpl19	1.244	0.01228
ENSMUSG00000014313	cytochrome c oxidase subunit 6C	Cox6c	1.24	0.03068
ENSMUSG00000025503	transaldolase 1	Taldo1	1.24	0.07008
ENSMUSG00000029994	annexin A4	Anxa4	1.237	0
ENSMUSG00000040385	protein phosphatase 1 catalytic subunit alpha	Ppp1ca	1.233	0.06111
ENSMUSG00000007892	ribosomal protein, large, P1	Rplp1	1.226	0.02516
ENSMUSG00000059070	ribosomal protein L18	Rpl18	1.171	0.01673
ENSMUSG00000028798	eukaryotic translation initiation factor 3, subunit I	Eif3i	1.162	0.07008
ENSMUSG00000038664	HECT and RLD domain containing E3 ubiquitin protein ligase family member 1	Herc1	0.965	0.0751

ENSMUSG00000003435	suppressor of Ty 5	Supt5	0.911	0.09235
ENSMUSG00000021497	thioredoxin domain containing 15	Txndc15	0.855	0.07412
ENSMUSG00000018750	zinc finger and BTB domain containing 4	Zbtb4	0.845	0.05527
ENSMUSG00000031622	transcriptional regulator, SIN3B (yeast)	Sin3b	0.84	0.07625
ENSMUSG00000054302	E2F-associated phosphoprotein	Eapp	0.823	0.10127
ENSMUSG00000063870	chromodomain helicase DNA binding protein 4	Chd4	0.817	0.09836
ENSMUSG00000037465	Kruppel-like factor 10	Klf10	0.815	0.09505
ENSMUSG00000027881	PRP38 pre-mRNA processing factor 38 (yeast) domain containing B	Prpf38b	0.81	0.07849
ENSMUSG00000001665	glutathione S-transferase, theta 3	Gstt3	0.808	0.10127
ENSMUSG00000032411	transcription factor Dp 2	Tfdp2	0.802	0.10127
ENSMUSG00000081094	ribosomal protein L19, pseudogene 11	Rpl19-ps11	0.797	0.07777
ENSMUSG00000028099	polymerase (RNA) III (DNA directed) polypeptide C	Polr3c	0.794	0.0788
ENSMUSG00000063576	kelch domain containing 3	Klhdc3	0.792	0.06311
ENSMUSG00000018167	START domain containing 3	Stard3	0.788	0.0788
ENSMUSG00000002820	autophagy related 4D, cysteine peptidase	Atg4d	0.78	0.09235
ENSMUSG00000009647	mitochondrial calcium uniporter	Mcu	0.777	0.05637
ENSMUSG00000030126	transmembrane and coiled coil domains 1	Tmcc1	0.776	0.09235
ENSMUSG00000069089	cyclin-dependent kinase 7	Cdk7	0.775	0.10127
ENSMUSG00000008036	adaptor-related protein complex 2, sigma 1 subunit	Ap2s1	0.773	0.09235
ENSMUSG00000009894	synaptosomal-associated protein, 47	Snap47	0.764	0.09505
ENSMUSG00000058586	serine hydrolase-like	Serhl	0.764	0.10127
ENSMUSG00000032187	SWI/SNF related, matrix associated, actin dependent regulator of chromatin, subfam	Smarca4	0.76	0.07412
ENSMUSG00000025369	SWI/SNF related, matrix associated, actin dependent regulator of chromatin, subfam	Smarcc2	0.759	0.07584
ENSMUSG00000022014	epithelial stromal interaction 1 (breast)	Epsti1	0.757	0.07584
ENSMUSG00000010453	KAT8 regulatory NSL complex subunit 3	Kansl3	0.757	0.09235
ENSMUSG00000039988	ankyrin repeat domain 13c	Ankrd13c	0.757	0.09505
ENSMUSG00000015766	epidermal growth factor receptor pathway substrate 8	Eps8	0.753	0.09235
ENSMUSG00000026466	torsin A interacting protein 1	Tor1aip1	0.751	0.04894
ENSMUSG00000054823	nuclear receptor binding SET domain protein 3	Nsd3	0.746	0.05968
ENSMUSG00000034083	coiled-coil domain containing 174	Ccdc174	0.739	0.05746
ENSMUSG00000028673	fucosidase, alpha-L- 1, tissue	Fuca1	0.739	0.09836
ENSMUSG00000074212	DnaJ heat shock protein family (Hsp40) member B14	Dnajb14	0.738	0.09235
ENSMUSG00000036188	ankyrin repeat and MYND domain containing 2	Ankmy2	0.736	0.10127
ENSMUSG00000027177	homeodomain interacting protein kinase 3	Hipk3	0.735	0.06525
ENSMUSG00000027010	solute carrier family 25 (mitochondrial carrier, Aralar), member 12	Slc25a12	0.734	0.07165
ENSMUSG00000071654	ubiquinol-cytochrome c reductase complex assembly factor 3	Uqcc3	0.734	0.07412
ENSMUSG00000005262	ubiquitin recognition factor in ER-associated degradation 1	Ufd1	0.729	0.09235
ENSMUSG00000017734	dysbindin (dystrobrevin binding protein 1) domain containing 2	Dbndd2	0.728	0.09235
ENSMUSG00000003559	arsenic (+3 oxidation state) methyltransferase	As3mt	0.727	0.09235
ENSMUSG00000049800	SERTA domain containing 2	Sertad2	0.725	0.06918
ENSMUSG00000056050	melanoma inhibitory activity 3	Mia3	0.724	0.09235
ENSMUSG00000027952	phosphomevalonate kinase	Pmvk	0.722	0.09235
ENSMUSG00000029427	zinc finger, CCHC domain containing 8	Zcchc8	0.718	0.10127
ENSMUSG00000079036	alkB homolog 1, histone H2A dioxygenase	Alkbh1	0.717	0.09235
ENSMUSG00000020311	endoplasmic reticulum lectin 1	Erlec1	0.716	0.05637
ENSMUSG00000033159	cyclin Pas1/PHO80 domain containing 1	Cnppd1	0.716	0.0788
ENSMUSG00000060538	transmembrane protein 219	Tmem219	0.715	0.09235
ENSMUSG00000042502	CD2 antigen (cytoplasmic tail) binding protein 2	Cd2bp2	0.714	0.09235
ENSMUSG00000057963	inositol 1,3,4-triphosphate 5/6 kinase	Itpk1	0.712	0.10127
ENSMUSG00000062906	histone deacetylase 10	Hdac10	0.71	0.09505
ENSMUSG00000025509	patatin-like phospholipase domain containing 2	Pnpla2	0.71	0.09836
ENSMUSG00000002329	magnesium-dependent phosphatase 1	Mdp1	0.709	0.05638
ENSMUSG00000055839	elongin B	Elob	0.709	0.0788
ENSMUSG00000031979	component of oligomeric golgi complex 2	Cog2	0.707	0.09235
ENSMUSG00000018287	sperm associated antigen 7	Spag7	0.706	0.05638
ENSMUSG00000040667	nucleoporin 88	Nup88	0.704	0.07016
ENSMUSG00000018322	translocase of outer mitochondrial membrane 34	Tomm34	0.703	0.10127
ENSMUSG00000031681	SMAD family member 1	Smad1	0.702	0.0975
ENSMUSG00000031557	pleckstrin homology domain-containing, family A (phosphoinositide binding specific)	Plekha2	0.696	0.05896
ENSMUSG00000002661	alkB homolog 7	Alkbh7	0.696	0.07453
ENSMUSG00000020644	inhibitor of DNA binding 2	Id2	0.696	0.07849
ENSMUSG00000015790	surfeit gene 1	Surf1	0.696	0.0788
ENSMUSG00000071796	RIKEN cDNA 6820431F20 gene	6820431F20Rik	0.696	0.0788
ENSMUSG00000026896	interferon induced with helicase C domain 1	Ifih1	0.696	0.10127
ENSMUSG00000032557	ubiquitin-like modifier activating enzyme 5	Uba5	0.695	0.07453
ENSMUSG00000069520	transmembrane protein 19	Tmem19	0.694	0.07234
ENSMUSG00000029190	DNA segment, Chr 5, ERATO Doi 579, expressed	D5Ertd579e	0.694	0.09836
ENSMUSG00000022216	proteasome (prosome, macropain) activator subunit 1 (PA28 alpha)	Psme1	0.692	0.07453
ENSMUSG00000066442	5, 10-methenyltetrahydrofolate synthetase	Mthfs	0.692	0.0788
ENSMUSG00000022201	zinc finger RNA binding protein	Zfr	0.691	0.07412
ENSMUSG00000020903	syntaxin 8	Stx8	0.688	0.10127
ENSMUSG00000039967	zinc finger protein 292	Zfp292	0.686	0.0788
ENSMUSG00000027951	adenosine deaminase, RNA-specific	Adar	0.684	0.05638
ENSMUSG00000091971	heat shock protein 1A	Hspa1a	0.684	0.06918
ENSMUSG00000020530	gametogenetin binding protein 2	Ggnbp2	0.684	0.10127
ENSMUSG00000042726	TRAF type zinc finger domain containing 1	Trafd1	0.682	0.06606
ENSMUSG00000062638	butyrophilin-like 1	Btnl1	0.682	0.07625
ENSMUSG00000020580	Rho-associated coiled-coil containing protein kinase 2	Rock2	0.681	0.07412
ENSMUSG00000030595	nuclear factor of kappa light polypeptide gene enhancer in B cells inhibitor, beta	Nfkbib	0.678	0.05636
ENSMUSG00000055200	SERTA domain containing 3	Sertad3	0.676	0.07412
ENSMUSG00000032712	retroelement silencing factor 1	Resf1	0.676	0.0788
ENSMUSG00000024044	erythrocyte membrane protein band 4.1 like 3	Epb41l3	0.675	0.07412
ENSMUSG00000024816	FERM domain containing 8	Frmd8	0.674	0.0751
ENSMUSG00000055720	ubiquitin-like 7 (bone marrow stromal cell-derived)	Ubl7	0.674	0.0788
ENSMUSG00000024055	cytochrome P450, family 4, subfamily f, polypeptide 13	Cyp4f13	0.673	0.07453
ENSMUSG00000025759	major facilitator superfamily domain containing 8	Mfsd8	0.669	0.0788
ENSMUSG00000026305	leucine rich repeat (in FLII) interacting protein 1	Lrrfip1	0.668	0.05638

ENSMUSG00000048379	suppressor of cytokine signaling 4	Socs4	0.668	0.05638
ENSMUSG00000033326	lysine (K)-specific demethylase 4A	Kdm4a	0.668	0.06525
ENSMUSG00000035268	protein kinase inhibitor, gamma	Pkig	0.667	0.06124
ENSMUSG00000046794	protein phosphatase 1, regulatory subunit 3B	Ppp1r3b	0.667	0.0788
ENSMUSG00000053929	cysteine and histidine rich 1	Cyhr1	0.667	0.09394
ENSMUSG00000071657	Berardinelli-Seip congenital lipodystrophy 2 (seipin)	Bscl2	0.662	0.05003
ENSMUSG00000014039	PR domain containing 15	Prdm15	0.662	0.06918
ENSMUSG00000048154	lysine (K)-specific methyltransferase 2D	Kmt2d	0.662	0.07453
ENSMUSG00000107068	predicted gene 42742	Gm42742	0.662	0.09235
ENSMUSG00000035152	adaptor-related protein complex 2, beta 1 subunit	Ap2b1	0.662	0.09836
ENSMUSG00000055204	ankyrin repeat domain 17	Ankrd17	0.661	0.09394
ENSMUSG00000050931	sphingomyelin synthase 2	Sgms2	0.658	0.09235
ENSMUSG00000036813	ectonucleoside triphosphate diphosphohydrolase 8	Entpd8	0.658	0.10127
ENSMUSG00000034269	SET domain containing 5	Setd5	0.656	0.09394
ENSMUSG00000003847	nuclear factor of activated T cells 5	Nfat5	0.655	0.09505
ENSMUSG00000038170	phosphodiesterase 4D interacting protein (myomegalin)	Pde4dip	0.654	0.07165
ENSMUSG00000067873	HIV TAT specific factor 1	Htatsf1	0.654	0.0788
ENSMUSG00000021188	thyroid hormone receptor interactor 11	Trip11	0.653	0.05191
ENSMUSG00000028274	RNA guanylyltransferase and 5'-phosphatase	Rngtt	0.653	0.07777
ENSMUSG00000040225	proline-rich coiled-coil 2C	Prrc2c	0.651	0.07777
ENSMUSG00000050103	alkylglycerol monooxygenase	Agmo	0.651	0.09235
ENSMUSG00000032508	myeloid differentiation primary response gene 88	Myd88	0.651	0.09836
ENSMUSG00000001323	serine racemase	Srr	0.65	0.04894
ENSMUSG00000019189	ring finger protein 145	Rnf145	0.65	0.05896
ENSMUSG00000034194	R3H domain and coiled-coil containing 1	R3hcc1	0.65	0.069
ENSMUSG00000030688	START domain containing 10	Stard10	0.65	0.10127
ENSMUSG00000040600	EPS8-like 3	Eps8l3	0.649	0.09235
ENSMUSG00000041733	coenzyme Q5 methyltransferase	Coq5	0.649	0.09505
ENSMUSG00000037295	low density lipoprotein receptor adaptor protein 1	Ldlrap1	0.648	0.07453
ENSMUSG00000029073	ceramide-1-phosphate transfer protein	Cptp	0.647	0.05003
ENSMUSG00000021809	nudix (nucleoside diphosphate linked moiety X)-type motif 13	Nudt13	0.647	0.07584
ENSMUSG00000042770	heme binding protein 1	Hebp1	0.646	0.10127
ENSMUSG00000010362	RAD52 motif 1	Rdm1	0.645	0.0577
ENSMUSG00000027078	ubiquitin-conjugating enzyme E2L 6	Ube2l6	0.644	0.05191
ENSMUSG00000038611	PHD and ring finger domains 1	Phrf1	0.644	0.07412
ENSMUSG00000019907	protein phosphatase 1, regulatory subunit 12A	Ppp1r12a	0.643	0.05003
ENSMUSG00000024130	ATP-binding cassette, sub-family A (ABC1), member 3	Abca3	0.642	0.0788
ENSMUSG00000018899	interferon regulatory factor 1	Irf1	0.641	0.09805
ENSMUSG00000024335	bromodomain containing 2	Brd2	0.64	0.07453
ENSMUSG00000024789	Janus kinase 2	Jak2	0.639	0.07412
ENSMUSG00000037416	Dmx-like 1	Dmxl1	0.639	0.09836
ENSMUSG00000074653	leucine rich repeat containing 31	Lrrc31	0.637	0.05947
ENSMUSG00000058056	palladin, cytoskeletal associated protein	Palld	0.636	0.06124
ENSMUSG00000015165	heterogeneous nuclear ribonucleoprotein L	Hnrnpl	0.636	0.07016
ENSMUSG00000068876	cingulin	Cgn	0.636	0.09235
ENSMUSG00000028251	thiosulfate sulfurtransferase (rhodanese)-like domain containing 3	Tstd3	0.636	0.09505
ENSMUSG00000019528	glycogenin	Gyg	0.635	0.09235
ENSMUSG00000028423	nuclear transcription factor, X-box binding 1	Nfx1	0.635	0.10127
ENSMUSG00000024002	bromodomain containing 4	Brd4	0.634	0.0788
ENSMUSG00000034390	c-Maf inducing protein	Cmip	0.631	0.04894
ENSMUSG00000037965	zinc finger CCCH type containing 7 A	Zc3h7a	0.631	0.05637
ENSMUSG00000039611	transmembrane protein 246	Tmem246	0.631	0.05968
ENSMUSG00000000134	transcription factor E3	Tfe3	0.631	0.07412
ENSMUSG00000001415	Smg-5 homolog, nonsense mediated mRNA decay factor (C. elegans)	Smg5	0.631	0.09235
ENSMUSG00000001794	calpain, small subunit 1	Capns1	0.63	0.06124
ENSMUSG00000024194	cutA divalent cation tolerance homolog	Cuta	0.63	0.09836
ENSMUSG00000042613	pre B cell leukemia transcription factor interacting protein 1	Pbxip1	0.629	0.05638
ENSMUSG00000018707	dynein cytoplasmic 1 heavy chain 1	Dync1h1	0.628	0.06405
ENSMUSG00000029684	Wiskott-Aldrich syndrome-like (human)	Wasl	0.628	0.09235
ENSMUSG00000054128	histocompatibility 2, T region locus 3	H2-T3	0.627	0.0788
ENSMUSG00000058833	required for excision 1-B domain containing	Rex1bd	0.627	0.10127
ENSMUSG00000001143	lectin, mannose-binding 2-like	Lman2l	0.626	0.09235
ENSMUSG00000022952	runt related transcription factor 1	Runx1	0.625	0.06525
ENSMUSG00000025139	toll interacting protein	Tollip	0.625	0.09235
ENSMUSG00000006494	pyruvate dehydrogenase kinase, isoenzyme 1	Pdk1	0.624	0.07453
ENSMUSG00000003423	PIH1 domain containing 1	Pih1d1	0.623	0.06405
ENSMUSG00000024867	phosphatidylinositol-4-phosphate 5-kinase, type 1 beta	Pip5k1b	0.623	0.09235
ENSMUSG00000041688	angiomotin	Amot	0.623	0.09235
ENSMUSG00000030982	VPS35 endosomal protein sorting factor like	Vps35l	0.621	0.09836
ENSMUSG00000038301	sorting nexin 10	Snx10	0.62	0.09235
ENSMUSG00000020385	CDC like kinase 4	Clk4	0.619	0.06806
ENSMUSG00000062729	protoporphyrinogen oxidase	Ppox	0.619	0.0788
ENSMUSG00000025261	HECT, UBA and WWE domain containing 1	Huwe1	0.619	0.09235
ENSMUSG00000022094	solute carrier family 39 (zinc transporter), member 14	Slc39a14	0.618	0.05265
ENSMUSG00000033444	sperm antigen with calponin homology and coiled-coil domains 1-like	Specc1l	0.618	0.05638
ENSMUSG00000052752	TNF receptor-associated factor 7	Traf7	0.617	0.04663
ENSMUSG00000028969	cyclin-dependent kinase 5	Cdk5	0.616	0.07453
ENSMUSG00000030909	ankyrin repeat and sterile alpha motif domain containing 4B	Anks4b	0.614	0.09235
ENSMUSG00000031393	methyl CpG binding protein 2	Mecp2	0.613	0.05003
ENSMUSG00000014355	anaphase promoting complex subunit 1	Anapc1	0.613	0.09836
ENSMUSG00000023939	mitochondrial ribosomal protein L14	Mrpl14	0.613	0.09836
ENSMUSG00000028248	PNN interacting serine/arginine-rich	Pnisr	0.612	0.05265
ENSMUSG00000040483	XIAP associated factor 1	Xaf1	0.61	0.05249
ENSMUSG00000005506	CUGBP, Elav-like family member 1	Celf1	0.61	0.07849
ENSMUSG00000073684	Fanconi anemia core complex associated protein 20	Faap20	0.61	0.0975
ENSMUSG00000001089	leucine zipper protein 1	Luzp1	0.609	0.02871
ENSMUSG00000037989	WNK lysine deficient protein kinase 2	Wnk2	0.609	0.06311

ENSMUSG00000028649	microtubule-actin crosslinking factor 1	Macf1	0.609	0.0788
ENSMUSG00000003346	abhydrolase domain containing 17A	Abhd17a	0.609	0.09235
ENSMUSG00000024845	transmembrane protein 134	Tmem134	0.608	0.04663
ENSMUSG00000020747	transmembrane protein 94	Tmem94	0.608	0.05527
ENSMUSG00000058793	CDP-diacylglycerol synthase (phosphatidate cytidylyltransferase) 2	Cds2	0.608	0.09235
ENSMUSG00000022914	bromodomain and WD repeat domain containing 1	Brwd1	0.606	0.07234
ENSMUSG00000033885	PX domain containing serine/threonine kinase	Pxk	0.606	0.09235
ENSMUSG00000033066	growth arrest specific 7	Gas7	0.605	0.10127
ENSMUSG00000106106		CT010467.1	0.604	0.05003
ENSMUSG00000029136	ribokinase	Rbks	0.604	0.07453
ENSMUSG00000032182	Yip1 domain family, member 2	Yipf2	0.604	0.07453
ENSMUSG00000047888	trinucleotide repeat containing 6b	Tnrc6b	0.603	0.07412
ENSMUSG00000045435	transmembrane protein 60	Tmem60	0.603	0.09235
ENSMUSG00000061650	mediator complex subunit 9	Med9	0.603	0.09235
ENSMUSG00000003226	RAN binding protein 2	Ranbp2	0.602	0.06311
ENSMUSG00000026634	angel homolog 2	Angel2	0.601	0.09235
ENSMUSG00000038872	zinc finger homeobox 3	Zfhx3	0.601	0.10127
ENSMUSG00000021930	SPRY domain containing 7	Spryd7	0.6	0.09836
ENSMUSG00000037750	family with sequence similarity 222, member B	Fam222b	0.599	0.07453
ENSMUSG00000092341	metastasis associated lung adenocarcinoma transcript 1 (non-coding RNA)	Malat1	0.598	0.05265
ENSMUSG00000022971	interferon (alpha and beta) receptor 2	Ifnar2	0.598	0.05968
ENSMUSG00000027365	transient receptor potential cation channel, subfamily M, member 7	Trpm7	0.597	0.07453
ENSMUSG00000048920	fukutin related protein	Fkrp	0.597	0.07777
ENSMUSG00000029407	USO1 vesicle docking factor	Uso1	0.597	0.10127
ENSMUSG00000044285	predicted gene 1821	Gm1821	0.596	0.04894
ENSMUSG00000056124	UDP-Gal:betaGlcNAc beta 1,4-galactosyltransferase, polypeptide 6	B4galt6	0.596	0.10127
ENSMUSG00000061758	aldo-keto reductase family 1, member B10 (aldose reductase)	Akr1b10	0.595	0.0788
ENSMUSG00000020638	cytidine monophosphate (UMP-CMP) kinase 2, mitochondrial	Cmpk2	0.595	0.09836
ENSMUSG00000026965	anaphase promoting complex subunit 2	Anapc2	0.594	0.07412
ENSMUSG00000034377	tubby like protein 4	Tulp4	0.593	0.05638
ENSMUSG00000003812	deoxyribonuclease II alpha	Dnase2a	0.593	0.10127
ENSMUSG00000032540	abhydrolase domain containing 5	Abhd5	0.592	0.05265
ENSMUSG00000030850	arginyltransferase 1	Ate1	0.592	0.07849
ENSMUSG00000022443	myosin, heavy polypeptide 9, non-muscle	Myh9	0.591	0.04376
ENSMUSG00000042202	solute carrier family 35, member E2	Slc35e2	0.591	0.0516
ENSMUSG00000025959	Kruppel-like factor 7 (ubiquitous)	Klf7	0.591	0.09235
ENSMUSG00000038708	golgi autoantigen, golgin subfamily a, 4	Golga4	0.591	0.09235
ENSMUSG00000063281	zinc finger protein 35	Zfp35	0.591	0.09235
ENSMUSG00000042682	selenoprotein K	Selenok	0.59	0.07412
ENSMUSG00000058952	complement component factor i	Cfi	0.59	0.0788
ENSMUSG00000041237	pyruvate kinase liver and red blood cell	Pklr	0.589	0.09836
ENSMUSG00000024963	DnaJ heat shock protein family (Hsp40) member C4	Dnajc4	0.587	0.05003
ENSMUSG00000034820	cleavage and polyadenylation specific factor 7	Cpsf7	0.587	0.0788
ENSMUSG00000026027	STE20-related kinase adaptor beta	Stradb	0.587	0.09394
ENSMUSG00000004455	protein phosphatase 1 catalytic subunit gamma	Ppp1cc	0.586	0.05638
ENSMUSG00000022949	chloride intracellular channel 6	Clic6	0.584	0.05249
ENSMUSG00000057133	chromodomain helicase DNA binding protein 6	Chd6	0.584	0.05249
ENSMUSG00000040725	heterogeneous nuclear ribonucleoprotein U-like 1	Hnrnpul1	0.584	0.07849
ENSMUSG00000025145	leucine rich repeat containing 45	Lrrc45	0.583	0.0975
ENSMUSG00000032437	STT3, subunit of the oligosaccharyltransferase complex, homolog B (S. cerevisiae)	Stt3b	0.582	0.04487
ENSMUSG00000015501	human immunodeficiency virus type I enhancer binding protein 2	Hivep2	0.582	0.05638
ENSMUSG00000022756	solute carrier family 7 (cationic amino acid transporter, y+ system), member 4	Slc7a4	0.582	0.09836
ENSMUSG00000037600	keratinocyte differentiation factor 1	Kdf1	0.582	0.09836
ENSMUSG00000039220	protein phosphatase 1, regulatory subunit 10	Ppp1r10	0.582	0.09836
ENSMUSG00000040945	regulator of chromosome condensation 2	Rcc2	0.581	0.0788
ENSMUSG00000045962	WNK lysine deficient protein kinase 1	Wnk1	0.58	0.06248
ENSMUSG00000033060	LIM domain only 7	Lmo7	0.579	0.07849
ENSMUSG00000023707	2-oxoglutarate and iron-dependent oxygenase domain containing 2	Ogfod2	0.579	0.0788
ENSMUSG00000002043	trafficking protein particle complex 6A	Trappc6a	0.578	0.05249
ENSMUSG00000073420	butyrophilin-like 5, pseudogene	Btnl5-ps	0.577	0.05638
ENSMUSG00000029364	WD repeat and SOCS box-containing 2	Wsb2	0.577	0.07453
ENSMUSG00000061859	PATJ, crumbs cell polarity complex component	Patj	0.576	0.06606
ENSMUSG00000030213	activating transcription factor 7 interacting protein	Atf7ip	0.576	0.07016
ENSMUSG00000019039	DALR anticodon binding domain containing 3	Dalrd3	0.576	0.0788
ENSMUSG00000050619	zinc finger SCAN domains 29	Zscan29	0.576	0.0788
ENSMUSG00000014873	surfeit gene 2	Surf2	0.576	0.10127
ENSMUSG00000066043	phosphatase and actin regulator 4	Phactr4	0.575	0.09235
ENSMUSG00000073411	histocompatibility 2, D region locus 1	H2-D1	0.575	0.09235
ENSMUSG00000054499	death effector domain-containing DNA binding protein 2	Dedd2	0.574	0.07453
ENSMUSG00000066621	tectonin beta-propeller repeat containing 1	Tecpr1	0.572	0.04894
ENSMUSG00000024787	sorting nexin 15	Snx15	0.572	0.07165
ENSMUSG00000037204	autophagy related 101	Atg101	0.572	0.0788
ENSMUSG00000078517	ER membrane protein complex subunit 1	Emc1	0.571	0.04376
ENSMUSG00000020190	MAP kinase-interacting serine/threonine kinase 2	Mknk2	0.571	0.09235
ENSMUSG00000101249	predicted gene 29216	Gm29216	0.57	0.04376
ENSMUSG00000038335	TSR1 20S rRNA accumulation	Tsr1	0.57	0.06405
ENSMUSG00000031391	L1 cell adhesion molecule	L1cam	0.569	0.06124
ENSMUSG00000002844	ADP-ribosylarginine hydrolase	Adprh	0.569	0.0788
ENSMUSG00000025533	argininosuccinate lyase	Asl	0.568	0.05638
ENSMUSG00000032615	5',3'-nucleotidase, mitochondrial	Nt5m	0.568	0.06124
ENSMUSG00000031154	OTU domain containing 5	Otud5	0.567	0.09514
ENSMUSG00000068246	apolipoprotein L 9b	Apol9b	0.566	0.05638
ENSMUSG00000026303	melanophilin	Mlph	0.566	0.06918
ENSMUSG00000020462	cilia and flagella associated protein 36	Cfap36	0.566	0.09235
ENSMUSG00000075520	MAM and LDL receptor class A domain containing 1	Malrd1	0.565	0.09235
ENSMUSG00000022964	transmembrane protein 50B	Tmem50b	0.564	0.04376
ENSMUSG00000045659	pleckstrin homology domain containing, family A member 7	Plekha7	0.564	0.04376

ENSMUSG00000035692	ISG15 ubiquitin-like modifier	Isg15	0.564	0.06015
ENSMUSG00000027829	cyclin L1	Ccnl1	0.564	0.09235
ENSMUSG00000046456	transmembrane protein 150B	Tmem150b	0.563	0.04894
ENSMUSG00000022109	mediator complex subunit 4	Med4	0.563	0.0516
ENSMUSG00000024036	solute carrier family 37 (glycerol-3-phosphate transporter), member 1	Slc37a1	0.562	0.05638
ENSMUSG00000026987	bromodomain adjacent to zinc finger domain, 2B	Baz2b	0.562	0.07016
ENSMUSG00000026867	GTPase activating protein and VPS9 domains 1	Gapvd1	0.562	0.09836
ENSMUSG00000043432	leukocyte receptor cluster (LRC) member 9	Leng9	0.56	0.06405
ENSMUSG00000030002	dual specificity phosphatase 11 (RNA/RNP complex 1-interacting)	Dusp11	0.56	0.09235
ENSMUSG00000036617	enhancer trap locus 4	Etl4	0.559	0.06606
ENSMUSG00000033306	LIM domain containing preferred translocation partner in lipoma	Lpp	0.559	0.09235
ENSMUSG00000038582	PTC7 protein phosphatase homolog	Pptc7	0.558	0.07849
ENSMUSG00000002365	sorting nexin 9	Snx9	0.557	0.06124
ENSMUSG00000042510	expressed sequence AA986860	AA986860	0.557	0.06124
ENSMUSG00000036698	argonaute RISC catalytic subunit 2	Ago2	0.557	0.07777
ENSMUSG00000031993	sorting nexin 19	Snx19	0.557	0.09235
ENSMUSG00000024290	Rho-associated coiled-coil containing protein kinase 1	Rock1	0.556	0.0788
ENSMUSG00000027806	TSC22 domain family, member 2	Tsc22d2	0.555	0.06328
ENSMUSG00000063652	solute carrier family 22 (organic cation transporter), member 21	Slc22a21	0.555	0.09235
ENSMUSG00000024369	negative elongation factor complex member E, Rdbp	Nelfe	0.554	0.04663
ENSMUSG00000053291	RAB4B, member RAS oncogene family	Rab4b	0.554	0.05249
ENSMUSG00000032582	RNA binding motif protein 6	Rbm6	0.554	0.06328
ENSMUSG00000062202	BTB (POZ) domain containing 9	Btbd9	0.554	0.06606
ENSMUSG00000029149	keratinocyte associated protein 3	Krtcap3	0.554	0.07165
ENSMUSG00000053754	chromodomain helicase DNA binding protein 8	Chd8	0.554	0.0788
ENSMUSG00000020564	ataxin 7-like 1	Atxn7l1	0.553	0.07584
ENSMUSG00000025571	trinucleotide repeat containing 6C	Tnrc6c	0.553	0.09836
ENSMUSG00000025534	glucuronidase, beta	Gusb	0.552	0.06015
ENSMUSG00000031600	vacuolar protein sorting 37A	Vps37a	0.551	0.07453
ENSMUSG00000018401	myotubularin related protein 4	Mtmr4	0.551	0.0788
ENSMUSG00000039361	phosphatidylinositol binding clathrin assembly protein	Picalm	0.55	0.04432
ENSMUSG00000005442	capicua transcriptional repressor	Cic	0.55	0.09235
ENSMUSG00000001127	Araf proto-oncogene, serine/threonine kinase	Araf	0.549	0.04376
ENSMUSG00000030779	retinoblastoma binding protein 6, ubiquitin ligase	Rbbp6	0.549	0.05947
ENSMUSG00000022761	leucine-zipper-like transcriptional regulator, 1	Lztr1	0.549	0.07849
ENSMUSG00000067212	histocompatibility 2, T region locus 23	H2-T23	0.548	0.10127
ENSMUSG00000036026	transmembrane protein 63b	Tmem63b	0.547	0.04894
ENSMUSG00000022906	poly (ADP-ribose) polymerase family, member 9	Parp9	0.547	0.05191
ENSMUSG00000034832	tet methylcytosine dioxygenase 3	Tet3	0.547	0.05638
ENSMUSG00000002812	flightless I actin binding protein	Flii	0.546	0.09235
ENSMUSG00000042323	polybromo 1	Pbrm1	0.546	0.09235
ENSMUSG00000032312	c-src tyrosine kinase	Csk	0.545	0.04376
ENSMUSG00000037210	family with sequence similarity 193, member A	Fam193a	0.545	0.05003
ENSMUSG00000068250	antagonist of mitotic exit network 1	Amn1	0.545	0.07453
ENSMUSG00000036712	CYLD lysine 63 deubiquitinase	Cyld	0.545	0.09235
ENSMUSG00000059555	torsin family 4, member A	Tor4a	0.544	0.05003
ENSMUSG00000027865	ganglioside-induced differentiation-associated-protein 2	Gdap2	0.543	0.04376
ENSMUSG00000039431	myotubularin related protein 7	Mtmr7	0.543	0.04376
ENSMUSG00000028488	SH3-domain GRB2-like 2	Sh3gl2	0.543	0.06405
ENSMUSG00000036528	PTPRF interacting protein, binding protein 2 (liprin beta 2)	Ppfibp2	0.543	0.0975
ENSMUSG00000030374	striatin, calmodulin binding protein 4	Strn4	0.542	0.04894
ENSMUSG00000037818	abhydrolase domain containing 18	Abhd18	0.542	0.05947
ENSMUSG00000006920	enhancer of zeste 1 polycomb repressive complex 2 subunit	Ezh1	0.542	0.06311
ENSMUSG00000024937	EH domain binding protein 1-like 1	Ehbp1l1	0.542	0.07453
ENSMUSG00000097354	RIKEN cDNA 2310001H17 gene	2310001H17Rik	0.541	0.05191
ENSMUSG00000039637	coronin 7	Coro7	0.541	0.07412
ENSMUSG00000019802	SEC63-like (S. cerevisiae)	Sec63	0.541	0.09235
ENSMUSG00000102349	predicted gene, 37376	Gm37376	0.54	0.05636
ENSMUSG00000056413	ArfGAP with dual PH domains 1	Adap1	0.54	0.06124
ENSMUSG00000028016	integrator complex subunit 12	Ints12	0.54	0.07412
ENSMUSG00000036552	ER membrane associated RNA degradation	Ermard	0.54	0.10127
ENSMUSG00000025266	guanine nucleotide binding protein-like 3 (nucleolar)-like	Gnl3l	0.539	0.04376
ENSMUSG00000035559	MPV17 mitochondrial membrane protein-like 2	Mpv17l2	0.539	0.05191
ENSMUSG00000014763	family with sequence similarity 120, member B	Fam120b	0.539	0.05249
ENSMUSG00000009076	zinc finger, matrin type 5	Zmat5	0.539	0.07453
ENSMUSG00000061207	serine/threonine kinase 19	Stk19	0.539	0.07453
ENSMUSG00000078945	NLR family, apoptosis inhibitory protein 2	Naip2	0.536	0.04432
ENSMUSG00000066258	tripartite motif-containing 12A	Trim12a	0.536	0.06311
ENSMUSG00000051853	ADP-ribosylation factor 3	Arf3	0.536	0.0788
ENSMUSG00000096780	transmembrane protein 181B, pseudogene	Tmem181b-ps	0.535	0.04894
ENSMUSG00000037466	tubulin epsilon and delta complex 1	Tedc1	0.535	0.07165
ENSMUSG00000020758	integrin beta 4	Itgb4	0.535	0.07849
ENSMUSG00000029145	eukaryotic translation initiation factor 2B, subunit 4 delta	Eif2b4	0.535	0.09235
ENSMUSG00000053286	tRNA methyltransferase 1 like	Trmt1l	0.534	0.05249
ENSMUSG00000024750	zinc finger, AN1-type domain 5	Zfand5	0.534	0.06405
ENSMUSG00000022175	low-density lipoprotein receptor-related protein 10	Lrp10	0.533	0.09836
ENSMUSG00000021945	zinc finger, MYM-type 2	Zmym2	0.532	0.09805
ENSMUSG00000056692	DNA segment, Chr 17, Wayne State University 92, expressed	D17Wsu92e	0.531	0.04376
ENSMUSG00000085890	tumor necrosis factor (ligand) superfamily, member 13, opposite strand	Tnfsf13os	0.53	0.0788
ENSMUSG00000025762	La ribonucleoprotein domain family, member 1B	Larp1b	0.529	0.04376
ENSMUSG00000105361	cDNA sequence AY036118	AY036118	0.529	0.04376
ENSMUSG00000061288	TAO kinase 3	Taok3	0.529	0.05003
ENSMUSG00000026269	arginyl aminopeptidase (aminopeptidase B)-like 1	Rnpepl1	0.529	0.06525
ENSMUSG00000020412	activating signal cointegrator 1 complex subunit 2	Ascc2	0.529	0.07453
ENSMUSG00000039501	zinc finger, NFX1-type containing 1	Znfx1	0.529	0.07453
ENSMUSG00000021375	kinesin family member 13A	Kif13a	0.529	0.09235
ENSMUSG00000047557	latexin	Lxn	0.528	0.02871

ENSMUSG00000001942	sialic acid acetylesterase	Siae	0.528	0.04663
ENSMUSG00000098178	predicted gene, 42418	Gm42418	0.527	0.05638
ENSMUSG00000018412	KAT8 regulatory NSL complex subunit 1	Kansl1	0.527	0.09505
ENSMUSG00000018821	arginine vasopressin-induced 1	Avpi1	0.526	0.04376
ENSMUSG00000023143	N-acetylglucosamine-1-phosphodiester alpha-N-acetylglucosaminidase	Nagpa	0.526	0.04376
ENSMUSG00000057594	ADP-ribosylation factor-like 16	Arl16	0.526	0.09836
ENSMUSG00000045095	membrane associated guanylate kinase, WW and PDZ domain containing 1	Magi1	0.525	0.09836
ENSMUSG00000028821	SYF2 homolog, RNA splicing factor (S. cerevisiae)	Syf2	0.524	0.02871
ENSMUSG00000024772	EH-domain containing 1	Ehd1	0.524	0.09805
ENSMUSG00000030970	C-terminal binding protein 2	Ctbp2	0.523	0.05191
ENSMUSG00000000631	myosin XVIIIA	Myo18a	0.523	0.05638
ENSMUSG00000034931	DEAH (Asp-Glu-Ala-His) box polypeptide 8	Dhx8	0.523	0.09235
ENSMUSG00000040505	ATP binding cassette subfamily G member 5	Abcg5	0.522	0.0516
ENSMUSG00000006589	adenine phosphoribosyl transferase	Aprt	0.521	0.02871
ENSMUSG00000075595	zinc finger protein 652	Zfp652	0.521	0.05636
ENSMUSG00000027412	lipin 3	Lpin3	0.521	0.05638
ENSMUSG00000006519	cytochrome b-245, alpha polypeptide	Cyba	0.521	0.10127
ENSMUSG00000008855	histone deacetylase 5	Hdac5	0.52	0.06311
ENSMUSG00000000759	tubulin, gamma complex associated protein 3	Tubgcp3	0.52	0.06829
ENSMUSG00000059810	regulator of G-protein signaling 3	Rgs3	0.52	0.09235
ENSMUSG00000041571	selenoprotein W	Selenow	0.519	0.05249
ENSMUSG00000041268	Dmx-like 2	Dmxl2	0.519	0.05636
ENSMUSG00000038213	TAP binding protein-like	Tapbpl	0.519	0.07625
ENSMUSG00000028413	UDP-Gal:betaGlcNAc beta 1,4- galactosyltransferase, polypeptide 1	B4galt1	0.518	0.04376
ENSMUSG00000040525	Casitas B-lineage lymphoma c	Cblc	0.518	0.04376
ENSMUSG00000033124	autophagy related 9A	Atg9a	0.518	0.05191
ENSMUSG00000038534	oxysterol binding protein-like 7	Osbpl7	0.518	0.05638
ENSMUSG00000037661	G protein-coupled receptor 160	Gpr160	0.518	0.07412
ENSMUSG00000057229	distal membrane arm assembly complex 2	Dmac2	0.518	0.07412
ENSMUSG00000068290	DDRGK domain containing 1	Ddrgk1	0.518	0.0751
ENSMUSG00000034254	1-acylglycerol-3-phosphate O-acyltransferase 1 (lysophosphatidic acid acyltransferas	Agpat1	0.518	0.0788
ENSMUSG00000037652	polyhomeotic 3	Phc3	0.518	0.10127
ENSMUSG00000013539	transport and golgi organization 2	Tango2	0.517	0.04663
ENSMUSG00000034057	myelin regulatory factor-like	Myrfl	0.517	0.0788
ENSMUSG00000002625	A kinase (PRKA) anchor protein 8-like	Akap8l	0.516	0.07453
ENSMUSG00000025207	sema domain, immunoglobulin domain (Ig), transmembrane domain (TM) and short	Sema4g	0.516	0.09235
ENSMUSG00000031540	K(lysine) acetyltransferase 6A	Kat6a	0.515	0.06525
ENSMUSG00000047909	ankyrin repeat domain 16	Ankrd16	0.513	0.07165
ENSMUSG00000020454	eukaryotic translation initiation factor 4E nuclear import factor 1	Eif4enif1	0.513	0.0788
ENSMUSG00000085287	RIKEN cDNA 4833418N02 gene	4833418N02Rik	0.513	0.0788
ENSMUSG00000066036	ubiquitin protein ligase E3 component n-recognin 4	Ubr4	0.513	0.09235
ENSMUSG00000031447	lysosomal-associated membrane protein 1	Lamp1	0.512	0.07453
ENSMUSG00000024042	salt inducible kinase 1	Sik1	0.51	0.06606
ENSMUSG00000024033	radial spoke head 1 homolog (Chlamydomonas)	Rsph1	0.509	0.09836
ENSMUSG00000010045	transmembrane protein 115	Tmem115	0.508	0.05003
ENSMUSG00000062822	RIKEN cDNA 4833420G17 gene	4833420G17Rik	0.508	0.05896
ENSMUSG00000018377	vascular endothelial zinc finger 1	Vezf1	0.508	0.10127
ENSMUSG00000036398	protein phosphatase 1, regulatory inhibitor subunit 11	Ppp1r11	0.507	0.06918
ENSMUSG00000029404	ADP-ribosylation factor-like 6 interacting protein 4	Arl6ip4	0.506	0.05638
ENSMUSG00000025408	DNA-damage inducible transcript 3	Ddit3	0.506	0.07777
ENSMUSG00000014349	ubiquitin-conjugating enzyme E2Z	Ube2z	0.505	0.05003
ENSMUSG00000039983	coiled-coil domain containing 32	Ccdc32	0.505	0.09836
ENSMUSG00000040997	abhydrolase domain containing 4	Abhd4	0.504	0.0788
ENSMUSG00000016664	protein kinase C and casein kinase substrate in neurons 2	Pacsin2	0.503	0.04894
ENSMUSG00000020455	tripartite motif-containing 11	Trim11	0.503	0.05637
ENSMUSG00000024457	tripartite motif-containing 26	Trim26	0.503	0.0788
ENSMUSG00000033170	caspase recruitment domain family, member 10	Card10	0.502	0.04376
ENSMUSG00000045665	major facilitator superfamily domain containing 5	Mfsd5	0.502	0.09394
ENSMUSG00000022503	nucleotide binding protein 1	Nubp1	0.501	0.07453
ENSMUSG00000026473	glutamate-ammonia ligase (glutamine synthetase)	Glul	0.501	0.07777
ENSMUSG00000053483	ubiquitin specific peptidase 21	Usp21	0.5	0.05265
ENSMUSG00000021156	zinc finger, MYND domain containing 11	Zmynd11	0.499	0.06124
ENSMUSG00000044881	cytochrome c oxidase assembly factor 4	Coa4	0.499	0.10127
ENSMUSG00000032826	ankyrin 2, brain	Ank2	0.498	0.05638
ENSMUSG00000024180	transmembrane protein 8	Tmem8	0.498	0.06124
ENSMUSG00000035248	terminal uridylyl transferase 7	Tut7	0.497	0.04376
ENSMUSG00000034714	tweety family member 2	Ttyh2	0.496	0.04376
ENSMUSG00000046822	solute carrier family 39 (zinc transporter), member 3	Slc39a3	0.496	0.05637
ENSMUSG00000081534	solute carrier family 48 (heme transporter), member 1	Slc48a1	0.496	0.06124
ENSMUSG00000054792	kelch-like 18	Klhl18	0.496	0.06918
ENSMUSG00000057738	spectrin alpha, non-erythrocytic 1	Sptan1	0.495	0.02871
ENSMUSG00000024085	mannosidase 2, alpha 1	Man2a1	0.495	0.05003
ENSMUSG00000103034	predicted pseudogene 8797	Gm8797	0.494	0.05003
ENSMUSG00000005078	JNK1/MAPK8-associated membrane protein	Jkamp	0.493	0.05637
ENSMUSG00000056305	ubiquitin specific peptidase 39	Usp39	0.493	0.05637
ENSMUSG00000029071	dishevelled segment polarity protein 1	Dvl1	0.492	0.05249
ENSMUSG00000015776	mediator complex subunit 22	Med22	0.492	0.07453
ENSMUSG00000090100	tau tubulin kinase 2	Ttbk2	0.492	0.0788
ENSMUSG00000030203	dual specificity phosphatase 16	Dusp16	0.492	0.09235
ENSMUSG00000018476	KDM1 lysine (K)-specific demethylase 6B	Kdm6b	0.491	0.06405
ENSMUSG00000050854	transmembrane protein 125	Tmem125	0.49	0.02871
ENSMUSG00000060657	meiosis regulator and mRNA stability 1	Marf1	0.49	0.04894
ENSMUSG00000003746	mannosidase 1, alpha	Man1a	0.49	0.05191
ENSMUSG00000038393	thioredoxin interacting protein	Txnip	0.49	0.05636
ENSMUSG00000022507	RIKEN cDNA 1810013L24 gene	1810013L24Rik	0.49	0.06405
ENSMUSG00000003316	golgi apparatus protein 1	Glg1	0.488	0.02871
ENSMUSG00000072825	centrosomal protein 170B	Cep170b	0.488	0.05527

ENSMUSG00000055320	TEA domain family member 1	Tead1	0.488	0.05637
ENSMUSG00000035649	zinc finger, CCHC domain containing 7	Zcchc7	0.487	0.04432
ENSMUSG00000030815	phosphorylase kinase, gamma 2 (testis)	Phkg2	0.487	0.05003
ENSMUSG00000003119	cyclin-dependent kinase 12	Cdk12	0.487	0.07412
ENSMUSG00000041297	cyclin-dependent kinase 13	Cdk13	0.487	0.07453
ENSMUSG00000008730	homeodomain interacting protein kinase 1	Hipk1	0.486	0.04663
ENSMUSG00000022529	zinc finger protein 263	Zfp263	0.486	0.05638
ENSMUSG00000055553	KxDL motif containing 1	Kxd1	0.486	0.06525
ENSMUSG00000024426	alpha tubulin acetyltransferase 1	Atat1	0.485	0.05003
ENSMUSG00000001751	alpha-N-acetylglucosaminidase (Sanfilippo disease IIIB)	Naglu	0.484	0.05003
ENSMUSG00000025354	DnaJ heat shock protein family (Hsp40) member C14	Dnajc14	0.484	0.05527
ENSMUSG00000033365	importin 13	Ipo13	0.483	0.05638
ENSMUSG00000048701	coiled-coil domain containing 6	Ccdc6	0.483	0.07412
ENSMUSG00000068036	afadin, adherens junction formation factor	Afdn	0.483	0.07412
ENSMUSG00000030315	vestigial like family member 4	Vgll4	0.483	0.07453
ENSMUSG00000030733	SH2B adaptor protein 1	Sh2b1	0.482	0.09235
ENSMUSG00000033499	La ribonucleoprotein domain family, member 4B	Larp4b	0.482	0.09235
ENSMUSG00000038733	WD repeat domain 26	Wdr26	0.481	0.05191
ENSMUSG00000061132	B cell linker	Blnk	0.481	0.05265
ENSMUSG00000036707	calcium binding protein 39	Cab39	0.481	0.07453
ENSMUSG00000034312	IQ motif and Sec7 domain 1	Iqsec1	0.481	0.10127
ENSMUSG00000033955	tankyrase 1 binding protein 1	Tnks1bp1	0.48	0.04894
ENSMUSG00000089832	Sh3kbp1 binding protein 1	Shkbp1	0.48	0.07412
ENSMUSG00000025220	meningioma expressed antigen 5 (hyaluronidase)	Mgea5	0.48	0.09235
ENSMUSG00000002871	transmembrane protein, adipocyte asscociated 1	Tpra1	0.479	0.0516
ENSMUSG00000038280	osteopetrosis associated transmembrane protein 1	Ostm1	0.479	0.09836
ENSMUSG00000034189	hydroxysteroid dehydrogenase like 1	Hsdl1	0.478	0.05873
ENSMUSG00000039262	proline-rich coiled-coil 2B	Prrc2b	0.478	0.06405
ENSMUSG00000040296	DEAD (Asp-Glu-Ala-Asp) box polypeptide 58	Ddx58	0.477	0.05638
ENSMUSG00000019863	glutaminyl-tRNA synthase (glutamine-hydrolyzing)-like 1	Qrsl1	0.476	0.0516
ENSMUSG00000020859	sperm associated antigen 9	Spag9	0.476	0.05636
ENSMUSG00000034563	cell cycle progression 1	Ccpg1	0.476	0.05637
ENSMUSG00000074746	PDZ domain containing 8	Pdzd8	0.476	0.0788
ENSMUSG00000030516	tight junction protein 1	Tjp1	0.476	0.09836
ENSMUSG00000045969	inhibitor of growth family, member 1	Ing1	0.475	0.04376
ENSMUSG00000055670	zinc finger, ZZ-type with EF hand domain 1	Zzef1	0.475	0.06311
ENSMUSG00000020415	pituitary tumor-transforming gene 1	Pttg1	0.475	0.07625
ENSMUSG00000042380	small integral membrane protein 12	Smim12	0.474	0.05637
ENSMUSG00000063273	N(alpha)-acetyltransferase 15, NatA auxiliary subunit	Naa15	0.474	0.06124
ENSMUSG00000003810	microtubule associated serine/threonine kinase 2	Mast2	0.474	0.06525
ENSMUSG00000060038	deoxyhypusine synthase	Dhps	0.474	0.06525
ENSMUSG00000015994	farnesyltransferase, CAAX box, alpha	Fnta	0.474	0.10127
ENSMUSG00000090115	ubiquitin specific peptidase 49	Usp49	0.473	0.05003
ENSMUSG00000042772	Smg-7 homolog, nonsense mediated mRNA decay factor (C. elegans)	Smg7	0.473	0.0516
ENSMUSG00000002748	bromodomain adjacent to zinc finger domain, 1B	Baz1b	0.473	0.09235
ENSMUSG00000030060	5-hydroxymethylcytosine (hmC) binding, ES cell specific	Hmces	0.473	0.09235
ENSMUSG00000093989	ribonuclease, RNase K	Rnasek	0.472	0.07412
ENSMUSG00000028803	NIPA-like domain containing 3	Nipal3	0.472	0.07777
ENSMUSG00000026470	syntaxin 6	Stx6	0.472	0.0788
ENSMUSG00000026197	zinc finger, AN1 type domain 2B	Zfand2b	0.471	0.04432
ENSMUSG00000051586	microtubule associated monooxygenase, calponin and LIM domain containing 3	Mical3	0.47	0.07016
ENSMUSG00000025153	fatty acid synthase	Fasn	0.47	0.10127
ENSMUSG00000032534	centrosomal protein 63	Cep63	0.47	0.10127
ENSMUSG00000048546	transducer of ERBB2, 2	Tob2	0.469	0.05191
ENSMUSG00000029104	huntingtin	Htt	0.467	0.05265
ENSMUSG00000037321	transporter 1, ATP-binding cassette, sub-family B (MDR/TAP)	Tap1	0.467	0.0788
ENSMUSG00000023030	solute carrier family 11 (proton-coupled divalent metal ion transporters), member 2	Slc11a2	0.467	0.09616
ENSMUSG00000061232	histocompatibility 2, K1, K region	H2-K1	0.466	0.05003
ENSMUSG00000020400	TNFAIP3 interacting protein 1	Tnip1	0.466	0.07016
ENSMUSG00000096188	CKLF-like MARVEL transmembrane domain containing 4	Cmtm4	0.465	0.05191
ENSMUSG00000039987	putative homeodomain transcription factor 2	Phtf2	0.464	0.04376
ENSMUSG00000036097	SMC5-SMC6 complex localization factor 2	Slf2	0.462	0.04376
ENSMUSG00000022552	SHANK-associated RH domain interacting protein	Sharpin	0.462	0.04894
ENSMUSG00000032265	terminal nucleotidyltransferase 5A	Tent5a	0.462	0.07165
ENSMUSG00000032410	5'-3' exoribonuclease 1	Xrn1	0.462	0.10127
ENSMUSG00000001583	tyrosine kinase, non-receptor, 1	Tnk1	0.461	0.07625
ENSMUSG00000063015	cyclin I	Ccni	0.461	0.09235
ENSMUSG00000024943	structural maintenance of chromosomes 5	Smc5	0.46	0.05003
ENSMUSG00000031072	ABCE maturation factor	LTO1	0.46	0.05638
ENSMUSG00000007659	BCL2-like 1	Bcl2l1	0.459	0.05265
ENSMUSG00000028080	LPS-responsive beige-like anchor	Lrba	0.459	0.10127
ENSMUSG00000059890	ubiquitination factor E4A	Ube4a	0.458	0.05003
ENSMUSG00000028345	testis expressed gene 10	Tex10	0.458	0.05527
ENSMUSG00000107304	predicted gene 43775	Gm43775	0.457	0.07625
ENSMUSG00000042644	inositol 1,4,5-triphosphate receptor 3	Itpr3	0.456	0.02871
ENSMUSG00000034947	transmembrane protein 106A	Tmem106a	0.455	0.0788
ENSMUSG00000033411	CTD (carboxy-terminal domain, RNA polymerase II, polypeptide A) small phosphatas	Ctdspl2	0.453	0.07016
ENSMUSG00000038902	pogo transposable element with ZNF domain	Pogz	0.453	0.09394
ENSMUSG00000020628	trafficking protein particle complex 12	Trappc12	0.453	0.10127
ENSMUSG00000028957	period circadian clock 3	Per3	0.452	0.02871
ENSMUSG00000002825	queuine tRNA-ribosyltransferase catalytic subunit 1	Qtrt1	0.452	0.04894
ENSMUSG00000019082	solute carrier family 25 (mitochondrial carrier, glutamate), member 22	Slc25a22	0.452	0.05003
ENSMUSG00000028150	RAR-related orphan receptor gamma	Rorc	0.452	0.05265
ENSMUSG00000065037	RNA, 7SK, nuclear	Rn7sk	0.452	0.0788
ENSMUSG00000042097	zinc finger protein 239	Zfp239	0.452	0.09235
ENSMUSG00000034708	granulin	Grn	0.451	0.02871
ENSMUSG00000056216	CCAAT/enhancer binding protein (C/EBP), gamma	Cebpg	0.451	0.04376

ENSMUSG00000024773	autophagy related 2A	Atg2a	0.451	0.09836
ENSMUSG00000042487	Leo1, Paf1/RNA polymerase II complex component	Leo1	0.45	0.0788
ENSMUSG00000045991	one cut domain, family member 2	Onecut2	0.45	0.0788
ENSMUSG00000024899	3'-phosphoadenosine 5'-phosphosulfate synthase 2	Papss2	0.45	0.10127
ENSMUSG00000033161	ATPase, Na+/K+ transporting, alpha 1 polypeptide	Atp1a1	0.449	0.02871
ENSMUSG00000006395	hydroxypyruvate isomerase (putative)	Hyi	0.449	0.05637
ENSMUSG00000018363	SMAD specific E3 ubiquitin protein ligase 2	Smurf2	0.449	0.05794
ENSMUSG00000049086	brain expressed myelocytomatosis oncogene	Bmyc	0.449	0.06124
ENSMUSG00000075376	ring finger and CCCH-type zinc finger domains 2	Rc3h2	0.447	0.04376
ENSMUSG00000041935	expressed sequence AW549877	AW549877	0.447	0.07453
ENSMUSG00000035818	pleckstrin homology domain containing, family S member 1	Plekhs1	0.447	0.09235
ENSMUSG00000052085	dedicator of cytokinesis 8	Dock8	0.447	0.09836
ENSMUSG00000039737	Prkr interacting protein 1 (IL11 inducible)	Prkrip1	0.446	0.05636
ENSMUSG00000022994	adenylate cyclase 6	Adcy6	0.446	0.07016
ENSMUSG00000073402	predicted gene 8909	Gm8909	0.446	0.0788
ENSMUSG00000033581	insulin-like growth factor 2 mRNA binding protein 2	Igf2bp2	0.445	0.06311
ENSMUSG00000007817	zinc finger, MIZ-type containing 1	Zmiz1	0.444	0.05638
ENSMUSG00000023952	GTP binding protein 2	Gtpbp2	0.444	0.07165
ENSMUSG00000094614			0.444	0.07412
ENSMUSG00000001054	required for meiotic nuclear division 5 homolog B	Rmnd5b	0.443	0.09235
ENSMUSG00000036098	myelin regulatory factor	Myrf	0.443	0.10127
ENSMUSG00000043909	transformation related protein 53 binding protein 1	Trp53bp1	0.442	0.04894
ENSMUSG00000033760	RNA binding motif protein 4B	Rbm4b	0.439	0.04376
ENSMUSG00000029471	calcium/calmodulin-dependent protein kinase kinase 2, beta	Camkk2	0.438	0.04894
ENSMUSG00000079557	membrane-associated ring finger (C3HC4) 2	March2	0.438	0.05638
ENSMUSG00000045038	protein kinase C, epsilon	Prkce	0.438	0.07412
ENSMUSG00000063171	ribosomal protein S4-like	Rps4l	0.438	0.07453
ENSMUSG00000024008	copine V	Cpne5	0.437	0.05638
ENSMUSG00000053877	Snf2-related CREBBP activator protein	Srcap	0.437	0.0577
ENSMUSG00000033352	mitogen-activated protein kinase kinase 4	Map2k4	0.437	0.06124
ENSMUSG00000028048	glucosidase, beta, acid	Gba	0.437	0.07453
ENSMUSG00000020827	misshapen-like kinase 1 (zebrafish)	Mink1	0.436	0.07234
ENSMUSG00000025521	transmembrane protein 192	Tmem192	0.436	0.07453
ENSMUSG00000028643	small vasohibin binding protein	Svbp	0.435	0.04432
ENSMUSG00000037486	additional sex combs like 2, transcriptional regulator	Asxl2	0.435	0.05637
ENSMUSG00000010914	pyruvate dehydrogenase complex, component X	Pdhx	0.435	0.07453
ENSMUSG00000025231	SUFU negative regulator of hedgehog signaling	Sufu	0.435	0.09836
ENSMUSG00000043592	unc-5 family C-terminal like	Unc5cl	0.434	0.0788
ENSMUSG00000022938	family with sequence similarity 3, member B	Fam3b	0.434	0.0975
ENSMUSG00000020740	golgi associated, gamma adaptin ear containing, ARF binding protein 3	Gga3	0.433	0.04894
ENSMUSG00000028634	human immunodeficiency virus type I enhancer binding protein 3	Hivep3	0.433	0.06015
ENSMUSG00000040631	docking protein 4	Dok4	0.433	0.09235
ENSMUSG00000067825	peroxisomal biogenesis factor 26	Pex26	0.433	0.09235
ENSMUSG00000037685	ATPase, aminophospholipid transporter (APLT), class I, type 8A, member 1	Atp8a1	0.432	0.04376
ENSMUSG00000022564	glutamate receptor, ionotropic, N-methyl D-aspartate-associated protein 1 (glutama	Grina	0.43	0.04376
ENSMUSG00000028954	negative regulator of ubiquitin-like proteins 1	Nub1	0.43	0.04894
ENSMUSG00000069844	SCO1 cytochrome c oxidase assembly protein	Sco1	0.43	0.07777
ENSMUSG00000025314	protein tyrosine phosphatase, receptor type, J	Ptprj	0.429	0.04376
ENSMUSG00000026307	selenocysteine lyase	Scly	0.429	0.04663
ENSMUSG00000071659	heterogeneous nuclear ribonucleoprotein U-like 2	Hnrnpul2	0.429	0.05003
ENSMUSG00000062519	zinc finger protein 398	Zfp398	0.428	0.051
ENSMUSG00000024151	mutS homolog 2	Msh2	0.428	0.05527
ENSMUSG00000030701	pleckstrin homology domain containing, family B (evectins) member 1	Plekhb1	0.428	0.0788
ENSMUSG00000032898	F-box protein 21	Fbxo21	0.427	0.10127
ENSMUSG00000033355	receptor transporter protein 4	Rtp4	0.425	0.05637
ENSMUSG00000036104	RAB3 GTPase activating protein subunit 1	Rab3gap1	0.425	0.07625
ENSMUSG00000038342	MLX interacting protein	Mlxip	0.425	0.10127
ENSMUSG00000024371	complement component 2 (within H-2S)	C2	0.424	0.05003
ENSMUSG00000030228	phosphatidylinositol-4-phosphate 3-kinase catalytic subunit type 2 gamma	Pik3c2g	0.424	0.05637
ENSMUSG00000037973	ITPR interacting domain containing 1	Itprid1	0.424	0.05873
ENSMUSG00000027411	VSP16 CORVET/HOPS core subunit	Vps16	0.423	0.0516
ENSMUSG00000023170	G protein pathway suppressor 2	Gps2	0.423	0.07453
ENSMUSG00000022100	exportin 7	Xpo7	0.423	0.10127
ENSMUSG00000030088	aldehyde dehydrogenase 1 family, member L1	Aldh1l1	0.422	0.06311
ENSMUSG00000026222	nuclear antigen Sp100	Sp100	0.421	0.04376
ENSMUSG00000015149	sirtuin 2	Sirt2	0.42	0.05191
ENSMUSG00000029119	mannosidase 2, alpha B2	Man2b2	0.42	0.0788
ENSMUSG00000019820	utrophin	Utrn	0.419	0.0516
ENSMUSG00000027272	ubiquitin protein ligase E3 component n-recognin 1	Ubr1	0.419	0.07453
ENSMUSG00000059714	flotillin 1	Flot1	0.418	0.05637
ENSMUSG00000025484	Bet1 golgi vesicular membrane trafficking protein like	Bet1l	0.418	0.07777
ENSMUSG00000042814	malignant T cell amplified sequence 2	Mcts2	0.418	0.09616
ENSMUSG00000033909	ubiquitin specific peptidase 36	Usp36	0.418	0.09836
ENSMUSG00000067336	bone morphogenetic protein receptor, type II (serine/threonine kinase)	Bmpr2	0.417	0.04376
ENSMUSG00000079065	cDNA sequence BC005561	BC005561	0.417	0.0788
ENSMUSG00000031389	Rho GTPase activating protein 4	Arhgap4	0.416	0.0516
ENSMUSG00000022974	PAX3 and PAX7 binding protein 1	Paxbp1	0.416	0.10127
ENSMUSG00000024847	aryl-hydrocarbon receptor-interacting protein	Aip	0.414	0.04376
ENSMUSG00000032305	family with sequence similarity 219, member B	Fam219b	0.414	0.06311
ENSMUSG00000028841	connector enhancer of kinase suppressor of Ras 1	Cnksr1	0.413	0.04376
ENSMUSG00000026104	signal transducer and activator of transcription 1	Stat1	0.413	0.0516
ENSMUSG00000027514	Z-DNA binding protein 1	Zbp1	0.413	0.05636
ENSMUSG00000024456	diaphanous related formin 1	Diaph1	0.412	0.06606
ENSMUSG00000021770	sterile alpha motif domain containing 8	Samd8	0.412	0.10127
ENSMUSG00000003352	calcium channel, voltage-dependent, beta 3 subunit	Cacnb3	0.411	0.02871
ENSMUSG00000020064	hect domain and RLD 4	Herc4	0.411	0.05636
ENSMUSG00000035569	ankyrin repeat domain 11	Ankrd11	0.411	0.06829

ENSMUSG00000038949	consortin, connexin sorting protein	Cnst	0.41	0.06525
ENSMUSG00000033862	cyclin-dependent kinase 10	Cdk10	0.41	0.07412
ENSMUSG00000041235	chromodomain helicase DNA binding protein 7	Chd7	0.409	0.06311
ENSMUSG00000030539	sema domain, immunoglobulin domain (Ig), transmembrane domain (TM) and short	Sema4b	0.408	0.04894
ENSMUSG00000017776	v-crk avian sarcoma virus CT10 oncogene homolog	Crk	0.408	0.05191
ENSMUSG00000037085	tRNA methyltranferase 12	Trmt12	0.408	0.09836
ENSMUSG00000019132	cDNA sequence BC005537	BC005537	0.407	0.04663
ENSMUSG00000061689	DLG associated protein 4	Dlgap4	0.407	0.0496
ENSMUSG00000006010	odr4 GPCR localization factor homolog	Odr4	0.407	0.07453
ENSMUSG00000035851	YTH domain containing 1	Ythdc1	0.407	0.0788
ENSMUSG00000042870	target of myb1 trafficking protein	Tom1	0.406	0.06311
ENSMUSG00000029389	DEAD (Asp-Glu-Ala-Asp) box polypeptide 55	Ddx55	0.405	0.05636
ENSMUSG00000034135	SIK family kinase 3	Sik3	0.405	0.10127
ENSMUSG00000026663	activating transcription factor 6	Atf6	0.404	0.04376
ENSMUSG00000022946	DOP1 leucine zipper like protein B	Dop1b	0.404	0.06405
ENSMUSG00000001672	MARVEL (membrane-associating) domain containing 3	Marveld3	0.403	0.04376
ENSMUSG00000041354	ral guanine nucleotide dissociation stimulator-like 2	Rgl2	0.403	0.07016
ENSMUSG00000023764	Sfi1 homolog, spindle assembly associated (yeast)	Sfi1	0.403	0.07412
ENSMUSG00000031592	pericentriolar material 1	Pcm1	0.403	0.09836
ENSMUSG00000021699	phosphodiesterase 4D, cAMP specific	Pde4d	0.402	0.09836
ENSMUSG00000027770	DEAH (Asp-Glu-Ala-His) box polypeptide 36	Dhx36	0.402	0.09836
ENSMUSG00000078853	interferon gamma induced GTPase	Igtp	0.401	0.04376
ENSMUSG00000029319	coenzyme Q2 4-hydroxybenzoate polyprenyltransferase	Coq2	0.401	0.04663
ENSMUSG00000054814	ubiquitin specific peptidase 46	Usp46	0.401	0.05003
ENSMUSG00000027639	SAM domain and HD domain, 1	Samhd1	0.401	0.05249
ENSMUSG00000040097	FLYWCH-type zinc finger 1	Flywch1	0.401	0.05636
ENSMUSG00000030530	furin (paired basic amino acid cleaving enzyme)	Furin	0.401	0.05638
ENSMUSG00000038671	ADP-ribosylation factor related protein 1	Arfrp1	0.401	0.05638
ENSMUSG00000035401	EMSY, BRCA2-interacting transcriptional repressor	Emsy	0.401	0.06311
ENSMUSG00000001786	F-box protein 7	Fbxo7	0.401	0.09235
ENSMUSG00000040537	a disintegrin and metallopeptidase domain 22	Adam22	0.401	0.09235
ENSMUSG00000037847	nicotinamide riboside kinase 1	Nmrk1	0.4	0.04376
ENSMUSG00000033542	Rho guanine nucleotide exchange factor (GEF) 5	Arhgef5	0.4	0.06525
ENSMUSG00000050390	expressed sequence C77080	C77080	0.399	0.05249
ENSMUSG00000021767	K(lysine) acetyltransferase 6B	Kat6b	0.399	0.05637
ENSMUSG00000042659	arrestin domain containing 4	Arrdc4	0.399	0.07165
ENSMUSG00000037110	Ral GTPase activating protein, alpha subunit 2 (catalytic)	Ralgapa2	0.399	0.10127
ENSMUSG00000033213	expressed sequence AA467197	AA467197	0.398	0.04376
ENSMUSG00000030122	parathymosin	Ptms	0.398	0.05638
ENSMUSG00000026626	protein phosphatase 2, regulatory subunit B', alpha	Ppp2r5a	0.398	0.07453
ENSMUSG00000035623	remodeling and spacing factor 1	Rsf1	0.397	0.04376
ENSMUSG00000096929	RIKEN cDNA A330023F24 gene	A330023F24Rik	0.397	0.05637
ENSMUSG00000039450	dicarbonyl L-xylulose reductase	Dcxr	0.397	0.07412
ENSMUSG00000021919	choline acetyltransferase	Chat	0.397	0.0788
ENSMUSG00000010051	hyaluronoglucosaminidase 1	Hyal1	0.396	0.04376
ENSMUSG00000026842	c-abl oncogene 1, non-receptor tyrosine kinase	Abl1	0.396	0.09235
ENSMUSG00000001700	GRAM domain containing 3	Gramd3	0.396	0.10127
ENSMUSG00000032280	transducin-like enhancer of split 3	Tle3	0.394	0.05265
ENSMUSG00000049076	ArfGAP with coiled-coil, ankyrin repeat and PH domains 2	Acap2	0.394	0.09836
ENSMUSG00000038418	early growth response 1	Egr1	0.394	0.10127
ENSMUSG00000047153	KH and NYN domain containing	Khnyn	0.393	0.04894
ENSMUSG00000029291	RUN and FYVE domain containing 3	Rufy3	0.393	0.09836
ENSMUSG00000028465	talin 1	Tln1	0.392	0.0516
ENSMUSG00000035673	strawberry notch 2	Sbno2	0.392	0.05896
ENSMUSG00000066406	A kinase (PRKA) anchor protein 13	Akap13	0.392	0.06918
ENSMUSG00000024222	FK506 binding protein 5	Fkbp5	0.392	0.0788
ENSMUSG00000034422	poly (ADP-ribose) polymerase family, member 14	Parp14	0.391	0.04894
ENSMUSG00000004364	cullin 3	Cul3	0.39	0.07234
ENSMUSG00000020196	calcineurin binding protein 1	Cabin1	0.39	0.0788
ENSMUSG00000064193	predicted gene 4735	Gm4735	0.389	0.07165
ENSMUSG00000078429	CTD (carboxy-terminal domain, RNA polymerase II, polypeptide A) small phosphatase	Ctdsp2	0.388	0.09235
ENSMUSG00000069633	peroxisomal biogenesis factor 11 gamma	Pex11g	0.387	0.04376
ENSMUSG00000041757	pleckstrin homology domain containing, family A member 6	Plekha6	0.387	0.05638
ENSMUSG00000029512	unc-51 like kinase 1	Ulk1	0.387	0.09235
ENSMUSG00000002897	interleukin 17 receptor A	Il17ra	0.386	0.06525
ENSMUSG00000031864	integrator complex subunit 10	Ints10	0.385	0.09836
ENSMUSG00000030232	AE binding protein 2	Aebp2	0.384	0.06606
ENSMUSG00000038000	adrenocortical dysplasia	Acd	0.383	0.10127
ENSMUSG00000027994	mitochondrial calcium uniporter dominant negative beta subunit	Mcub	0.382	0.05637
ENSMUSG00000021357	exocyst complex component 2	Exoc2	0.381	0.04376
ENSMUSG00000040865	INO80 complex subunit D	Ino80d	0.381	0.04376
ENSMUSG00000004798	unc-51 like kinase 2	Ulk2	0.381	0.09235
ENSMUSG00000070327	ring finger protein 213	Rnf213	0.38	0.02871
ENSMUSG00000090112	SNF2 histone linker PHD RING helicase	Shprh	0.38	0.09505
ENSMUSG00000044465	family with sequence similarity 160, member A2	Fam160a2	0.379	0.04376
ENSMUSG00000014550	rabenosyn, RAB effector	Rbsn	0.379	0.10127
ENSMUSG00000037960	caspase recruitment domain family, member 19	Card19	0.378	0.04376
ENSMUSG00000046697	ectonucleotide pyrophosphatase/phosphodiesterase 7	Enpp7	0.378	0.10127
ENSMUSG00000073096	leucine rich repeat containing 61	Lrrc61	0.377	0.05191
ENSMUSG00000024382	excision repair cross-complementing rodent repair deficiency, complementation gro	Ercc3	0.377	0.0577
ENSMUSG00000024866	aspartoacylase (aminoacylase) 3	Acy3	0.376	0.05794
ENSMUSG00000026240	COP9 signalosome subunit 7B	Cops7b	0.375	0.05637
ENSMUSG00000029513	protein kinase, AMP-activated, beta 1 non-catalytic subunit	Prkab1	0.375	0.09235
ENSMUSG00000062234	cyclin G associated kinase	Gak	0.375	0.09235
ENSMUSG00000030302	ATPase, Ca++ transporting, plasma membrane 2	Atp2b2	0.375	0.09836
ENSMUSG00000079334	N(alpha)-acetyltransferase 80, NatH catalytic subunit	Naa80	0.373	0.07016
ENSMUSG00000106864	general transcription factor IIIC, polypeptide 2, beta	Gtf3c2	0.372	0.05249

ENSMUSG00000029833	tripartite motif-containing 24	Trim24	0.371	0.04894
ENSMUSG00000035168	tetratricopeptide repeat, ankyrin repeat and coiled-coil containing 1	Tanc1	0.37	0.07165
ENSMUSG00000039578	coiled-coil serine rich 1	Ccser1	0.37	0.07412
ENSMUSG00000031093	dedicator of cytokinesis 11	Dock11	0.369	0.09235
ENSMUSG00000042492	TBC1 domain family, member 10b	Tbc1d10b	0.368	0.05638
ENSMUSG00000061028	CLK4-associating serine/arginine rich protein	Clasrp	0.367	0.09235
ENSMUSG00000072770	proacrosin binding protein	Acrbp	0.367	0.09505
ENSMUSG00000055932	fat mass and obesity associated	Fto	0.366	0.05638
ENSMUSG00000021025	nuclear factor of kappa light polypeptide gene enhancer in B cells inhibitor, alpha	Nfkbia	0.365	0.06328
ENSMUSG00000027580	helicase with zinc finger 2, transcriptional coactivator	Helz2	0.364	0.04432
ENSMUSG00000085152	predicted gene 11496	Gm11496	0.363	0.05638
ENSMUSG00000001542	elongation factor RNA polymerase II 2	Ell2	0.363	0.09394
ENSMUSG00000035629	RUN domain and cysteine-rich domain containing, Beclin 1-interacting protein	Rubcn	0.362	0.069
ENSMUSG00000041642	kinesin family member 21B	Kif21b	0.362	0.09836
ENSMUSG00000038256	B cell CLL/lymphoma 9	Bcl9	0.361	0.07016
ENSMUSG00000027244	autophagy related 13	Atg13	0.361	0.07234
ENSMUSG00000012114	mediator complex subunit 15	Med15	0.361	0.10127
ENSMUSG00000027695	phospholipase D1	Pld1	0.36	0.04376
ENSMUSG00000028756	PTEN induced putative kinase 1	Pink1	0.359	0.04663
ENSMUSG00000030650	transmembrane channel-like gene family 5	Tmc5	0.359	0.04894
ENSMUSG00000029577	ubiquitin protein ligase E3B	Ube3b	0.359	0.05637
ENSMUSG00000032120	C2 calcium-dependent domain containing 2-like	C2cd2l	0.359	0.07584
ENSMUSG00000054021	sirtuin 5	Sirt5	0.358	0.10127
ENSMUSG00000058318	PHD finger protein 21A	Phf21a	0.356	0.05636
ENSMUSG00000097750	predicted gene 4673	Gm4673	0.356	0.06918
ENSMUSG00000038002	cramped chromatin regulator homolog 1	Cramp1l	0.356	0.09836
ENSMUSG00000023809	ribosomal protein S6 kinase, polypeptide 2	Rps6ka2	0.355	0.06606
ENSMUSG00000041777	corepressor interacting with RBPJ, 1	Cir1	0.354	0.07016
ENSMUSG00000056121	fasciculation and elongation protein zeta 2 (zygin II)	Fez2	0.354	0.0788
ENSMUSG00000008200	formin binding protein 4	Fnbp4	0.353	0.09836
ENSMUSG00000039176	polymerase (DNA directed), gamma	Polg	0.352	0.09235
ENSMUSG00000002983	avian reticuloendotheliosis viral (v-rel) oncogene related B	Relb	0.351	0.05003
ENSMUSG00000028060	KH domain containing 4, pre-mRNA splicing factor	Khdc4	0.35	0.04376
ENSMUSG00000072812	AHNAK nucleoprotein 2	Ahnak2	0.349	0.07584
ENSMUSG00000023923	TBC1 domain family, member 5	Tbc1d5	0.349	0.0788
ENSMUSG00000038843	glucosaminyl (N-acetyl) transferase 1, core 2	Gcnt1	0.348	0.04432
ENSMUSG00000024429	guanine nucleotide binding protein-like 1	Gnl1	0.348	0.07453
ENSMUSG00000040761	spen family transcription repressor	Spen	0.348	0.10127
ENSMUSG00000021972	homeobox containing 1	Hmbox1	0.347	0.04663
ENSMUSG00000042249	G protein-coupled receptor kinase 3	Grk3	0.347	0.09235
ENSMUSG00000020109	DnaJ heat shock protein family (Hsp40) member B12	Dnajb12	0.346	0.06124
ENSMUSG00000028042	zinc finger and BTB domain containing 7B	Zbtb7b	0.346	0.07412
ENSMUSG00000102976	zinc finger CCCH type containing 11A	Zc3h11a	0.345	0.04376
ENSMUSG00000017830	DEXH (Asp-Glu-X-His) box polypeptide 58	Dhx58	0.344	0.0516
ENSMUSG00000004846	procollagen-lysine, 2-oxoglutarate 5-dioxygenase 3	Plod3	0.344	0.05249
ENSMUSG00000001229	dipeptidylpeptidase 9	Dpp9	0.343	0.069
ENSMUSG00000028976	solute carrier family 2 (facilitated glucose transporter), member 5	Slc2a5	0.343	0.09505
ENSMUSG00000002833	HDGF like 2	Hdgfl2	0.342	0.0516
ENSMUSG00000061436	homeodomain interacting protein kinase 2	Hipk2	0.341	0.06525
ENSMUSG00000056116	histocompatibility 2, T region locus 22	H2-T22	0.339	0.06311
ENSMUSG00000035545	leukocyte receptor cluster (LRC) member 8	Leng8	0.339	0.06328
ENSMUSG00000029536	glutamyl-tRNA(Gln) amidotransferase, subunit C	Gatc	0.339	0.07412
ENSMUSG00000045098	lysine methyltransferase 5B	Kmt5b	0.338	0.04376
ENSMUSG00000021978	exostoses (multiple)-like 3	Extl3	0.337	0.02871
ENSMUSG00000031823	zinc finger, DHHC domain containing 7	Zdhhc7	0.337	0.09235
ENSMUSG00000024399	lymphotoxin B	Ltb	0.336	0.05637
ENSMUSG00000033253	seizure threshold 2	Szt2	0.336	0.05968
ENSMUSG00000048796	cytochrome b-561 domain containing 1	Cyb561d1	0.336	0.07016
ENSMUSG00000032497	leucine rich repeat (in FLII) interacting protein 2	Lrrfip2	0.333	0.10127
ENSMUSG00000032322	proline-serine-threonine phosphatase-interacting protein 1	Pstpip1	0.332	0.05638
ENSMUSG00000038811	guanine nucleotide binding protein (G protein), gamma transducing activity polypept	Gngt2	0.332	0.09836
ENSMUSG00000028420	transmembrane protein 38B	Tmem38b	0.331	0.04432
ENSMUSG00000022095	family with sequence similarity 160, member B2	Fam160b2	0.331	0.04894
ENSMUSG00000025499	Harvey rat sarcoma virus oncogene	Hras	0.33	0.04894
ENSMUSG00000031902	nuclear factor of activated T cells, cytoplasmic, calcineurin dependent 3	Nfatc3	0.33	0.05265
ENSMUSG00000009555	cyclin-dependent kinase 9 (CDC2-related kinase)	Cdk9	0.33	0.09235
ENSMUSG00000035835	phospholipid phosphatase related 3	Plppr3	0.33	0.0975
ENSMUSG00000028826	macoilin 1	Maco1	0.329	0.05896
ENSMUSG00000026565	POU domain, class 2, transcription factor 1	Pou2f1	0.329	0.07016
ENSMUSG00000064220	histone cluster 2, H2aa1	Hist2h2aa1	0.328	0.06248
ENSMUSG00000057346	apolipoprotein L 9a	Apol9a	0.327	0.04376
ENSMUSG00000024163	mitogen-activated protein kinase 8 interacting protein 3	Mapk8ip3	0.327	0.05638
ENSMUSG00000036555	IQ motif containing E	Iqce	0.327	0.0788
ENSMUSG00000040721	zinc finger homeobox 2	Zfhx2	0.327	0.09235
ENSMUSG00000037235	Max dimerization protein 4	Mxd4	0.325	0.09836
ENSMUSG00000038013	WAS/WASL interacting protein family, member 2	Wipf2	0.324	0.04376
ENSMUSG00000042116	von Willebrand factor A domain containing 1	Vwa1	0.324	0.09235
ENSMUSG00000059981	TAO kinase 2	Taok2	0.323	0.07412
ENSMUSG00000046718	bone marrow stromal cell antigen 2	Bst2	0.322	0.04376
ENSMUSG00000054484	transmembrane protein 62	Tmem62	0.322	0.05638
ENSMUSG00000020806	rhomboid 5 homolog 2	Rhbdf2	0.322	0.0788
ENSMUSG00000020755	SAP30 binding protein	Sap30bp	0.321	0.04376
ENSMUSG00000066800	ribonuclease L (2', 5'-oligoisoadenylate synthetase-dependent)	Rnasel	0.321	0.07412
ENSMUSG00000040327	cullin 9	Cul9	0.319	0.07412
ENSMUSG00000034190	charged multivesicular body protein 7	Chmp7	0.319	0.07453
ENSMUSG00000026600	sterol O-acyltransferase 1	Soat1	0.318	0.04376
ENSMUSG00000002325	interferon regulatory factor 9	Irf9	0.317	0.04376

ENSMUSG00000026102	inositol polyphosphate-1-phosphatase	Inpp1	0.317	0.06405
ENSMUSG00000036333	kinase D-interacting substrate 220	Kidins220	0.317	0.07412
ENSMUSG00000005698	CCCTC-binding factor	Ctcf	0.316	0.0516
ENSMUSG00000054385	carcinoembryonic antigen-related cell adhesion molecule 2	Ceacam2	0.315	0.07625
ENSMUSG00000030727	rabaptin, RAB GTPase binding effector protein 2	Rabep2	0.314	0.04894
ENSMUSG00000041263	RUN and SH3 domain containing 1	Rusc1	0.314	0.05638
ENSMUSG00000061536	SEC22 homolog C, vesicle trafficking protein	Sec22c	0.314	0.07849
ENSMUSG00000044072	echinoderm microtubule associated protein like 6	Eml6	0.313	0.05003
ENSMUSG00000041769	protein phosphatase 2, regulatory subunit B, delta	Ppp2r2d	0.312	0.06606
ENSMUSG00000044786	zinc finger protein 36	Zfp36	0.311	0.07412
ENSMUSG00000024483	ankyrin repeat and KH domain containing 1	Ankhd1	0.31	0.04376
ENSMUSG00000049739	zinc finger protein 646	Zfp646	0.31	0.07016
ENSMUSG00000020100	solute carrier family 29 (nucleoside transporters), member 3	Slc29a3	0.309	0.04376
ENSMUSG00000071647	echinoderm microtubule associated protein like 3	Eml3	0.309	0.05249
ENSMUSG00000024052	lipin 2	Lpin2	0.309	0.09394
ENSMUSG00000029647	PAN3 poly(A) specific ribonuclease subunit	Pan3	0.308	0.04376
ENSMUSG00000000131	exportin 6	Xpo6	0.306	0.05746
ENSMUSG00000031617	transmembrane protein 184C	Tmem184c	0.306	0.0788
ENSMUSG00000035498	CUB domain containing protein 1	Cdcp1	0.305	0.04894
ENSMUSG00000070576	meningioma 1	Mn1	0.305	0.04894
ENSMUSG00000026014	Ras association (RalGDS/AF-6) and pleckstrin homology domains 1	Raph1	0.304	0.02871
ENSMUSG00000035202	leucyl-tRNA synthetase, mitochondrial	Lars2	0.304	0.04376
ENSMUSG00000052707	trinucleotide repeat containing 6a	Tnrc6a	0.304	0.04376
ENSMUSG00000032548	solute carrier organic anion transporter family, member 2a1	Slco2a1	0.304	0.04663
ENSMUSG00000038324	transient receptor potential cation channel, subfamily C, member 4 associated prote	Trpc4ap	0.303	0.04432
ENSMUSG00000022983	SR-related CTD-associated factor 4	Scaf4	0.303	0.0788
ENSMUSG00000027762	succinate receptor 1	Sucnr1	0.303	0.0788
ENSMUSG00000024807	synovial apoptosis inhibitor 1, synoviolin	Syvn1	0.302	0.06525
ENSMUSG00000028760	eukaryotic translation initiation factor 4 gamma, 3	Eif4g3	0.301	0.04894
ENSMUSG00000013973	death effector domain-containing	Dedd	0.301	0.05003
ENSMUSG00000004815	diacylglycerol kinase, theta	Dgkq	0.3	0.069
ENSMUSG00000026991	plakophilin 4	Pkp4	0.299	0.07412
ENSMUSG00000033460	armadillo repeat containing, X-linked 1	Armcx1	0.299	0.09514
ENSMUSG00000104291	RIKEN cDNA A130071D04 gene	A130071D04Rik	0.298	0.06525
ENSMUSG00000052751	replication initiator 1	Repin1	0.298	0.07625
ENSMUSG00000021944	GATA binding protein 4	Gata4	0.297	0.09836
ENSMUSG00000029860	zyxin	Zyx	0.297	0.10127
ENSMUSG00000071203	NLR family, apoptosis inhibitory protein 5	Naip5	0.296	0.04894
ENSMUSG00000063450	spectrin repeat containing, nuclear envelope 2	Syne2	0.295	0.069
ENSMUSG00000038495	OTU domain containing 7B	Otud7b	0.294	0.04663
ENSMUSG00000045252	zinc finger protein 574	Zfp574	0.293	0.04663
ENSMUSG00000032046	abhydrolase domain containing 12	Abhd12	0.293	0.05794
ENSMUSG00000038784	CCR4-NOT transcription complex, subunit 4	Cnot4	0.292	0.04376
ENSMUSG00000007987	intraflagellar transport 22	Ift22	0.292	0.05003
ENSMUSG00000031298	adhesion G protein-coupled receptor G2	Adgrg2	0.292	0.06405
ENSMUSG00000003573	homer scaffolding protein 3	Homer3	0.292	0.10127
ENSMUSG00000037627	regulator of G-protein signalling 22	Rgs22	0.29	0.05638
ENSMUSG00000022768	coiled-coil domain containing 116	Ccdc116	0.29	0.09836
ENSMUSG00000039759	THAP domain containing, apoptosis associated protein 3	Thap3	0.286	0.07453
ENSMUSG00000041836	protein tyrosine phosphatase, receptor type, E	Ptpre	0.285	0.04432
ENSMUSG00000047228	alpha-2-macroglobulin like 1	A2ml1	0.281	0.05638
ENSMUSG00000045211	nudix (nucleoside diphosphate linked moiety X)-type motif 18	Nudt18	0.281	0.0788
ENSMUSG00000101599	predicted gene, 20342	Gm20342	0.281	0.10127
ENSMUSG00000062604	serine/arginine-rich protein specific kinase 2	Srpk2	0.28	0.09235
ENSMUSG00000029408	ATP-binding cassette, sub-family B (MDR/TAP), member 9	Abcb9	0.279	0.04376
ENSMUSG00000040651	family with sequence similarity 208, member A	Fam208a	0.278	0.02871
ENSMUSG00000034621	G patch domain containing 8	Gpatch8	0.278	0.05527
ENSMUSG00000028186	urate oxidase	Uox	0.278	0.05637
ENSMUSG00000027219	solute carrier family 28 (sodium-coupled nucleoside transporter), member 2	Slc28a2	0.275	0.05637
ENSMUSG00000028556	dedicator of cytokinesis 7	Dock7	0.275	0.09836
ENSMUSG00000000148	BRCA1-associated ATM activator 1	Brat1	0.273	0.05637
ENSMUSG00000020329	polymerase (RNA) mitochondrial (DNA directed)	Polrmt	0.271	0.04376
ENSMUSG00000020604	arylsulfatase G	Arsg	0.271	0.05638
ENSMUSG00000005886	nuclear receptor coactivator 2	Ncoa2	0.271	0.09235
ENSMUSG00000030655	SMG1 homolog, phosphatidylinositol 3-kinase-related kinase (C. elegans)	Smg1	0.27	0.05191
ENSMUSG00000102163	predicted gene, 36945	Gm36945	0.27	0.07453
ENSMUSG00000026103	glutaminase	Gls	0.27	0.09394
ENSMUSG00000032727	MIER family member 3	Mier3	0.269	0.05638
ENSMUSG00000026811	ST6 (alpha-N-acetyl-neuraminyl-2,3-beta-galactosyl-1,3)-N-acetylgalactosaminide alp	St6galnac6	0.268	0.05896
ENSMUSG00000040033	signal transducer and activator of transcription 2	Stat2	0.267	0.04376
ENSMUSG00000037103	DDB1 and CUL4 associated factor 15	Dcaf15	0.264	0.05637
ENSMUSG00000046020	protein O-fucosyltransferase 1	Pofut1	0.264	0.09235
ENSMUSG00000017697	adenosine deaminase	Ada	0.261	0.04376
ENSMUSG00000038371	SET binding factor 2	Sbf2	0.261	0.04376
ENSMUSG00000036591	Rho GTPase activating protein 21	Arhgap21	0.261	0.05637
ENSMUSG00000025366	extended synaptotagmin-like protein 1	Esyt1	0.26	0.07849
ENSMUSG00000013787	euchromatic histone lysine N-methyltransferase 2	Ehmt2	0.259	0.02871
ENSMUSG00000035597	pre-mRNA processing factor 39	Prpf39	0.258	0.07453
ENSMUSG00000025993	solute carrier family 40 (iron-regulated transporter), member 1	Slc40a1	0.257	0.10127
ENSMUSG00000006191	CDK5 regulatory subunit associated protein 1-like 1	Cdkal1	0.256	0.0516
ENSMUSG00000045538	DEAD (Asp-Glu-Ala-Asp) box polypeptide 28	Ddx28	0.255	0.05003
ENSMUSG00000044042	formin 1	Fmn1	0.255	0.10127
ENSMUSG00000020883	F-box and leucine-rich repeat protein 20	Fbxl20	0.254	0.09394
ENSMUSG00000046311	zinc finger protein 62	Zfp62	0.254	0.10127
ENSMUSG00000003345	casein kinase 1, gamma 2	Csnk1g2	0.253	0.0516
ENSMUSG00000063550	nucleoporin 98	Nup98	0.253	0.09836
ENSMUSG00000047084	neugrin, neurite outgrowth associated	Ngrn	0.252	0.04376

ENSMUSG00000027784	protein phosphatase 1 (formerly 2C)-like	Ppm1l	0.252	0.05638
ENSMUSG00000001750	T cell, immune regulator 1, ATPase, H+ transporting, lysosomal V0 protein A3	Tcirg1	0.251	0.05638
ENSMUSG00000021061	spectrin beta, erythrocytic	Sptb	0.251	0.0788
ENSMUSG00000020099	unc-5 netrin receptor B	Unc5b	0.249	0.04663
ENSMUSG00000094103	RIKEN cDNA 1700047I17 gene 2	1700047I17Rik2	0.249	0.05947
ENSMUSG00000025486	sirtuin 3	Sirt3	0.248	0.04376
ENSMUSG00000027739	RAB33B, member RAS oncogene family	Rab33b	0.248	0.069
ENSMUSG00000022389	thyrotroph embryonic factor	Tef	0.246	0.10127
ENSMUSG00000043629	RIKEN cDNA 1700019D03 gene	1700019D03Rik	0.245	0.04376
ENSMUSG00000068205	MACRO domain containing 2	Macrod2	0.245	0.05746
ENSMUSG00000034543	microrchidia 2A	Morc2a	0.244	0.05265
ENSMUSG00000060798	inturned planar cell polarity protein	Intu	0.244	0.06829
ENSMUSG00000046982	teashirt zinc finger family member 1	Tshz1	0.243	0.05003
ENSMUSG00000041609	BICD family like cargo adaptor 1	Bicdl1	0.242	0.07412
ENSMUSG00000033039	microtubule associated monooxygenase, calponin and LIM domain containing -like 1	Micall1	0.241	0.09505
ENSMUSG00000032333	stomatin-like 1	Stoml1	0.239	0.04432
ENSMUSG00000024851	phosphatidylinositol transfer protein, membrane-associated 1	Pitpnm1	0.239	0.05249
ENSMUSG00000037104	suppressor of cytokine signaling 5	Socs5	0.238	0.10127
ENSMUSG00000036002	family with sequence similarity 214, member B	Fam214b	0.237	0.04376
ENSMUSG00000104339	RIKEN cDNA C130089K02 gene	C130089K02Rik	0.237	0.07453
ENSMUSG00000032340	neogenin	Neo1	0.236	0.04894
ENSMUSG00000020173	cordon-bleu WH2 repeat	Cobl	0.235	0.02871
ENSMUSG00000004677	myosin IXb	Myo9b	0.235	0.05636
ENSMUSG00000051413	pleiomorphic adenoma gene-like 2	Plagl2	0.231	0.10127
ENSMUSG00000018398	septin 8	Sept8	0.23	0.05794
ENSMUSG00000014778	formin homology 2 domain containing 1	Fhod1	0.23	0.07412
ENSMUSG00000097715	G protein-coupled receptor 137B, pseudogene	Gpr137b-ps	0.228	0.04663
ENSMUSG00000038145	SNF related kinase	Snrk	0.227	0.04376
ENSMUSG00000032637	ataxin 2-like	Atxn2l	0.227	0.0788
ENSMUSG00000047284	neuralized E3 ubiquitin protein ligase 4	Neurl4	0.227	0.09836
ENSMUSG00000046862	PRAME family member 8	Pramef8	0.226	0.0788
ENSMUSG00000042439	zinc finger protein 532	Zfp532	0.225	0.0788
ENSMUSG00000032690	2'-5' oligoadenylate synthetase 2	Oas2	0.223	0.04376
ENSMUSG00000032036	kirre like nephrin family adhesion molecule 3	Kirrel3	0.223	0.05003
ENSMUSG00000029074	tubulin tyrosine ligase-like family, member 10	Ttll10	0.222	0.05249
ENSMUSG00000020495	smg-8 homolog, nonsense mediated mRNA decay factor (C. elegans)	Smg8	0.222	0.06606
ENSMUSG00000025875	tetraspanin 17	Tspan17	0.221	0.09235
ENSMUSG00000055862	IZUMO family member 4	Izumo4	0.218	0.10127
ENSMUSG00000028468	RAB6A GEF compex partner 1	Rgp1	0.215	0.10127
ENSMUSG00000091475	RIKEN cDNA 2810468N07 gene	2810468N07Rik	0.212	0.07412
ENSMUSG00000032596	ubiquitin-like modifier activating enzyme 7	Uba7	0.212	0.09836
ENSMUSG00000036636	chloride channel, voltage-sensitive 7	Clcn7	0.21	0.02871
ENSMUSG00000037962	refilin A	Rflna	0.209	0.04894
ENSMUSG00000071356	regenerating islet-derived 3 beta	Reg3b	0.208	0.02871
ENSMUSG00000001855	nucleoporin 214	Nup214	0.208	0.04487
ENSMUSG00000031586	RNA binding protein gene with multiple splicing	Rbpms	0.206	0.09235
ENSMUSG00000031816	methenyltetrahydrofolate synthetase domain containing	Mthfsd	0.204	0.04376
ENSMUSG00000005417	myosin phosphatase Rho interacting protein	Mprip	0.204	0.05636
ENSMUSG00000020436	gamma-aminobutyric acid (GABA) A receptor, subunit gamma 2	Gabrg2	0.203	0.05638
ENSMUSG00000021850	coiled-coil domain containing 198	ccdc198	0.201	0.05638
ENSMUSG00000029781	FK506 binding protein 9	Fkbp9	0.201	0.07165
ENSMUSG00000029154	cell wall biogenesis 43 C-terminal homolog	Cwh43	0.199	0.06015
ENSMUSG00000042677	zinc finger CCCH type containing 12A	Zc3h12a	0.196	0.06311
ENSMUSG00000021384	sushi domain containing 3	Susd3	0.194	0.0788
ENSMUSG00000021408	receptor (TNFRSF)-interacting serine-threonine kinase 1	Ripk1	0.188	0.04376
ENSMUSG00000078185	choroideremia-like	Chml	0.188	0.10127
ENSMUSG00000028737	aldehyde dehydrogenase 4 family, member A1	Aldh4a1	0.187	0.04376
ENSMUSG00000106651	predicted gene 42608	Gm42608	0.186	0.09805
ENSMUSG00000038485	suppressor of cytokine signaling 7	Socs7	0.183	0.04894
ENSMUSG00000037526	autophagy related 14	Atg14	0.182	0.0788
ENSMUSG00000067547	predicted pseudogene 7666	Gm7666	0.182	0.09505
ENSMUSG00000060550	histocompatibility 2, Q region locus 7	H2-Q7	0.179	0.02871
ENSMUSG00000103693	predicted gene, 37529	Gm37529	0.175	0.09836
ENSMUSG00000059149	major facilitator superfamily domain containing 4A	Mfsd4a	0.172	0.02871
ENSMUSG00000054426	RIKEN cDNA A930005H10 gene	A930005H10Rik	0.171	0.0788
ENSMUSG00000031669	GINS complex subunit 3 (Psf3 homolog)	Gins3	0.171	0.09235
ENSMUSG00000005534	insulin receptor	Insr	0.169	0.07165
ENSMUSG00000029729	zinc finger with KRAB and SCAN domains 1	Zkscan1	0.168	0.05636
ENSMUSG00000003154	forkhead box J2	Foxj2	0.168	0.05638
ENSMUSG00000071064	zinc finger protein 827	Zfp827	0.168	0.0788
ENSMUSG00000036986	promyelocytic leukemia	Pml	0.167	0.05746
ENSMUSG00000040896	potassium voltage-gated channel, Shal-related family, member 3	Kcnd3	0.165	0.05637
ENSMUSG00000036057	protein tyrosine phosphatase, non-receptor type 23	Ptpn23	0.165	0.0788
ENSMUSG00000036672	centromere protein T	Cenpt	0.165	0.09836
ENSMUSG00000034653	YTH domain containing 2	Ythdc2	0.164	0.09836
ENSMUSG00000025722	WD repeat domain 73	Wdr73	0.161	0.06405
ENSMUSG00000034382	expressed sequence AI661453	AI661453	0.161	0.0788
ENSMUSG00000036686	coiled-coil and C2 domain containing 1A	Cc2d1a	0.16	0.09235
ENSMUSG00000042507	ELM2 and Myb/SANT-like domain containing 1	Elmsan1	0.16	0.09235
ENSMUSG00000033434	GTP binding protein 6 (putative)	Gtpbp6	0.157	0.0788
ENSMUSG00000051306	ubiquitin specific peptidase 42	Usp42	0.152	0.07412
ENSMUSG00000049037	C-type lectin domain family 4, member a1	Clec4a1	0.151	0.05637
ENSMUSG00000097101	RIKEN cDNA 1810034E14 gene	1810034E14Rik	0.148	0.09836
ENSMUSG00000001518	integrin alpha FG-GAP repeat containing 2	Itfg2	0.145	0.10127
ENSMUSG00000040253	guanylate binding protein 7	Gbp7	0.141	0.06311
ENSMUSG00000019518	adaptor-related protein complex AP-4, mu 1	Ap4m1	0.139	0.05003
ENSMUSG00000049323	Smith-Magenis syndrome chromosome region, candidate 8 homolog (human)	Smcr8	0.138	0.04376

ENSMUSG00000047635	RIKEN cDNA 2810006K23 gene	2810006K23Rik	0.138	0.09836
ENSMUSG00000038305	spermatogenesis associated, serine-rich 2-like	Spats2l	0.137	0.05265
ENSMUSG00000062646	glucosidase, alpha; neutral C	Ganc	0.136	0.02871
ENSMUSG00000027134	lysophosphatidylcholine acyltransferase 4	Lpcat4	0.136	0.09235
ENSMUSG00000026499	acyl-Coenzyme A binding domain containing 3	Acbd3	0.136	0.09836
ENSMUSG00000018765	fragile X mental retardation, autosomal homolog 2	Fxr2	0.135	0.0788
ENSMUSG00000035930	carbohydrate sulfotransferase 4	Chst4	0.135	0.09394
ENSMUSG00000021775	nuclear receptor subfamily 1, group D, member 2	Nr1d2	0.13	0.09836
ENSMUSG00000029673	autism susceptibility candidate 2	Auts2	0.127	0.0788
ENSMUSG00000057691	zinc finger protein 746	Zfp746	0.126	0.10127
ENSMUSG00000062157	interferon lambda receptor 1	Ifnlr1	0.123	0.05638
ENSMUSG00000024219	ankyrin repeat and SAM domain containing 1	Anks1	0.122	0.10127
ENSMUSG00000037791	PHD finger protein 12	Phf12	0.121	0.10127
ENSMUSG00000027457	syntaphilin	Snph	0.115	0.09836
ENSMUSG00000039648	kynurenine aminotransferase 1	Kyat1	0.112	0.09836
ENSMUSG00000010601	apolipoprotein L 7a	Apol7a	0.111	0.04376
ENSMUSG00000022722	ADP-ribosylation factor-like 6	Arl6	0.108	0.09235
ENSMUSG00000036473	TBC1 domain family, member 24	Tbc1d24	0.107	0.09235
ENSMUSG00000021366	human immunodeficiency virus type I enhancer binding protein 1	Hivep1	0.099	0.10127
ENSMUSG00000019699	thymoma viral proto-oncogene 3	Akt3	0.098	0.10127
ENSMUSG00000035953	phosphatidylinositol-4,5-bisphosphate 4-phosphatase 1	Pip4p1	0.097	0.07453
ENSMUSG00000038347	t-complex-associated testis expressed 2	Tcte2	0.096	0.07165
ENSMUSG00000028532	cache domain containing 1	Cachd1	0.095	0.05265
ENSMUSG00000038976	protein phosphatase 1, regulatory subunit 9B	Ppp1r9b	0.095	0.0788
ENSMUSG00000037921	DEAD (Asp-Glu-Ala-Asp) box polypeptide 60	Ddx60	0.094	0.09805
ENSMUSG00000040524	zinc finger protein 609	Zfp609	0.093	0.04376
ENSMUSG00000050350	G protein-coupled receptor 18	Gpr18	0.093	0.09235
ENSMUSG00000070462	talin rod domain containing 1	Tlnrd1	0.093	0.09836
ENSMUSG00000032735	actin binding LIM protein family, member 3	Ablim3	0.091	0.07412
ENSMUSG00000042388	DLG associated protein 3	Dlgap3	0.089	0.10127
ENSMUSG00000015597	zinc finger protein 318	Zfp318	0.088	0.10127
ENSMUSG00000071176	Rho guanine nucleotide exchange factor (GEF) 10	Arhgef10	0.082	0.05265
ENSMUSG00000057335	centrosomal protein 170	Cep170	0.081	0.09836
ENSMUSG00000024622	HMG box domain containing 3	Hmgxb3	0.08	0.07165
ENSMUSG00000032496	lactotransferrin	Ltf	0.079	0.04376
ENSMUSG00000005580	adenylate cyclase 9	Adcy9	0.073	0.02871
ENSMUSG00000027519	RAB22A, member RAS oncogene family	Rab22a	0.07	0.07453
ENSMUSG00000092482	predicted gene 20531	Gm20531	0.067	0.09235
ENSMUSG00000057596	tripartite motif-containing 30D	Trim30d	0.065	0.04894
ENSMUSG00000020279	interleukin 9 receptor	Il9r	0.064	0.04376
ENSMUSG00000021782	discs large MAGUK scaffold protein 5	Dlg5	0.054	0.06124
ENSMUSG00000042810	KRAB-A domain containing 1	Krba1	0.054	0.06918
ENSMUSG00000009418	neuron navigator 1	Nav1	0.04	0.07453
ENSMUSG00000026791	solute carrier family 2, (facilitated glucose transporter), member 8	Slc2a8	0.038	0.07412
ENSMUSG00000073437	RIKEN cDNA D330041H03 gene	D330041H03Rik	0.035	0.04663
ENSMUSG00000039989	chromobox 4	Cbx4	0.035	0.05638
ENSMUSG00000001062	VPS9 domain containing 1	Vps9d1	0.031	0.04894
ENSMUSG00000030359	PZP, alpha-2-macroglobulin like	Pzp	0.031	0.05527
ENSMUSG00000073409	histocompatibility 2, Q region locus 6	H2-Q6	0.022	0.04376
ENSMUSG00000026514	cornichon family AMPA receptor auxiliary protein 3	Cnih3	0	0.04376
ENSMUSG00000029167	peroxisome proliferative activated receptor, gamma, coactivator 1 alpha	Ppargc1a	0	0.04376
ENSMUSG00000035529	PR domain containing 4	Prdm4	0	0.04663
ENSMUSG00000066829	zinc finger protein 810	Zfp810	0	0.04663
ENSMUSG00000000409	lymphocyte protein tyrosine kinase	Lck	0	0.0516
ENSMUSG00000004947	deltex 2, E3 ubiquitin ligase	Dtx2	0	0.0516
ENSMUSG00000084960	RIKEN cDNA B430010I23 gene	B430010I23Rik	0	0.0516
ENSMUSG00000000386	MX dynamin-like GTPase 1	Mx1	0	0.05191
ENSMUSG00000027983	cytochrome P450, family 2, subfamily u, polypeptide 1	Cyp2u1	0	0.05637
ENSMUSG00000059187	family with sequence similarity 19, member A1	Fam19a1	0	0.05637
ENSMUSG00000020131	proprotein convertase subtilisin/kexin type 4	Pcsk4	0	0.05638
ENSMUSG00000031165	Wiskott-Aldrich syndrome	Was	0	0.05638
ENSMUSG00000106375	predicted gene 43361	Gm43361	0	0.05638
ENSMUSG00000039934	gamma-secretase activating protein	Gsap	0	0.06124
ENSMUSG00000032028	neurexophilin and PC-esterase domain family, member 2	Nxpe2	0	0.06248
ENSMUSG00000020070	RUN and FYVE domain-containing 2	Rufy2	0	0.06311
ENSMUSG00000031907	zinc finger protein 90	Zfp90	0	0.06311
ENSMUSG00000032298	nei endonuclease VIII-like 1 (E. coli)	Neil1	0	0.06311
ENSMUSG00000060843	catenin (cadherin associated protein), alpha 3	Ctnna3	0	0.06328
ENSMUSG00000037773	PC-esterase domain containing 1A	Pced1a	0	0.06405
ENSMUSG00000014470	ring finger protein 166	Rnf166	0	0.06606
ENSMUSG00000033792	ATPase, Cu++ transporting, alpha polypeptide	Atp7a	0	0.06918
ENSMUSG00000057176	coiled-coil domain containing 189	Ccdc189	0	0.06918
ENSMUSG00000049265	potassium channel, subfamily K, member 3	Kcnk3	0	0.07016
ENSMUSG00000078786	cDNA sequence BC024978	BC024978	0	0.07016
ENSMUSG00000040151	heparan sulfate 2-O-sulfotransferase 1	Hs2st1	0	0.07165
ENSMUSG00000061482	histone cluster 1, H4d	Hist1h4d	0	0.07165
ENSMUSG00000017376	nemo like kinase	Nlk	0	0.07412
ENSMUSG00000055413	histocompatibility 2, Q region locus 5	H2-Q5	0	0.07412
ENSMUSG00000057137	transmembrane protein 140	Tmem140	0	0.07412
ENSMUSG00000063268	poly (ADP-ribose) polymerase family, member 10	Parp10	0	0.07412
ENSMUSG00000031728	zinc finger protein 821	Zfp821	0	0.07453
ENSMUSG00000032243	integrin alpha 11	Itga11	0	0.07849
ENSMUSG00000032839	transient receptor potential cation channel, subfamily C, member 1	Trpc1	0	0.07849
ENSMUSG00000014351	gastric inhibitory polypeptide	Gip	0	0.0788
ENSMUSG00000020805	solute carrier family 13 (sodium-dependent citrate transporter), member 5	Slc13a5	0	0.0788
ENSMUSG00000021214	aldo-keto reductase family 1, member C18	Akr1c18	0	0.0788
ENSMUSG00000045409	tripartite motif-containing 39	Trim39	0	0.0788

ENSMUSG00000051435	forkhead-associated (FHA) phosphopeptide binding domain 1	Fhad1	0	0.0788
ENSMUSG00000085471	RIKEN cDNA 4933423P22 gene	4933423P22Rik	0	0.0788
ENSMUSG00000010936	Vac14 homolog (S. cerevisiae)	Vac14	0	0.09235
ENSMUSG00000026082	REV1, DNA directed polymerase	Rev1	0	0.09235
ENSMUSG00000026548	SLAM family member 9	Slamf9	0	0.09235
ENSMUSG00000027544	nuclear factor of activated T cells, cytoplasmic, calcineurin dependent 2	Nfatc2	0	0.09235
ENSMUSG00000028931	potassium voltage-gated channel, shaker-related subfamily, beta member 2	Kcnab2	0	0.09235
ENSMUSG00000030323	intraflagellar transport 122	Ift122	0	0.09235
ENSMUSG00000032997	chondroitin polymerizing factor	Chpf	0	0.09235
ENSMUSG00000037007	zinc finger protein 113	Zfp113	0	0.09235
ENSMUSG00000039308	N-deacetylase/N-sulfotransferase (heparan glucosaminyl) 2	Ndst2	0	0.09235
ENSMUSG00000049539	histone cluster 1, H1a	Hist1h1a	0	0.09235
ENSMUSG00000063954	histone cluster 2, H2aa2	Hist2h2aa2	0	0.09235
ENSMUSG00000073434	WD repeat domain 90	Wdr90	0	0.09235
ENSMUSG00000086740	predicted gene 17029	Gm17029	0	0.09235
ENSMUSG00000107317	predicted gene, 19719	Gm19719	0	0.09235
ENSMUSG00000107928	predicted gene 45140	Gm45140	0	0.09235
ENSMUSG00000026311	ankyrin repeat and SOCS box-containing 1	Asb1	0	0.09394
ENSMUSG00000029605	2'-5' oligoadenylate synthetase 1B	Oas1b	0	0.09394
ENSMUSG00000037594	clathrin binding box of aftiphilin containing 1	Clba1	0	0.09394
ENSMUSG00000022957	intersectin 1 (SH3 domain protein 1A)	Itsn1	0	0.09505
ENSMUSG00000032733	sorting nexin 33	Snx33	0	0.09505
ENSMUSG00000046985	transmembrane anterior posterior transformation 1	Tapt1	0	0.09505
ENSMUSG00000097730	predicted gene, 26588	Gm26588	0	0.09514
ENSMUSG00000034893	component of oligomeric golgi complex 3	Cog3	0	0.0975
ENSMUSG00000045176	BLOC-1 related complex subunit 6	Borcs6	0	0.09836
ENSMUSG00000057329	B cell leukemia/lymphoma 2	Bcl2	0	0.09836
ENSMUSG00000075327	zinc finger and BTB domain containing 2	Zbtb2	0	0.09836
ENSMUSG00000099931	predicted gene 29358	Gm29358	0	0.09836
ENSMUSG00000048997	ataxin 7-like 2	Atxn7l2	0	0.10127
ENSMUSG00000056268	DENN/MADD domain containing 1B	Dennd1b	0	0.10127
ENSMUSG00000095105	EDAR (ectodysplasin-A receptor)-associated death domain	Edaradd	0	0.10127
ENSMUSG00000097320	transmembrane protein 147, opposite strand	Tmem147os	0	0.10127
ENSMUSG00000097601	predicted gene, 26660	Gm26660	0	0.10127

Supplementary data 1: List of differently regulated genes in small intestinal tuft cells under HFD-feeding vs RFD-feeding conditions for 22 weeks.

GeneID	GeneName	GeneSymbol	HFD/RFD 22wk_fold change	*q-value*_ HFD/RFD_22wk
ENSMUSG00000066072	cytochrome P450, family 4, subfamily a, polypeptide 10	Cyp4a10	3108949549	0
ENSMUSG00000072949	acyl-CoA thioesterase 1	Acot1	1460661791	0
ENSMUSG00000032418	malic enzyme 1, NADP(+)-dependent, cytosolic	Me1	383194522.5	0
ENSMUSG00000037348	progestin and adipoQ receptor family member VII	Paqr7	13.659	0
ENSMUSG00000027306	nucleolar and spindle associated protein 1	Nusap1	9.721	0.08294
ENSMUSG00000027875	3-hydroxy-3-methylglutaryl-Coenzyme A synthase 2	Hmgcs2	9.662	0
ENSMUSG00000020918	K(lysine) acetyltransferase 2A	Kat2a	4.897	0.08294
ENSMUSG00000095649	predicted gene 8979	Gm8979	3.862	0.08294
ENSMUSG00000031403	dyskeratosis congenita 1, dyskerin	Dkc1	3.644	0.03808
ENSMUSG00000055114	annexin A13	Anxa13	3.568	0.01426
ENSMUSG00000008540	microsomal glutathione S-transferase 1	Mgst1	3.401	0
ENSMUSG00000022797	transferrin receptor	Tfrc	3.193	0.03808
ENSMUSG00000041506	RRP9, small subunit (SSU) processome component, homolog (yeast)	Rrp9	3.09	0.07129
ENSMUSG00000021696	ELOVL family member 7, elongation of long chain fatty acids (yeast)	Elovl7	3.078	0.01426
ENSMUSG00000075054	Yae1 domain containing 1	Yae1d1	2.995	0.08294
ENSMUSG00000022698	N(alpha)-acetyltransferase 50, NatE catalytic subunit	Naa50	2.843	0.08294
ENSMUSG00000025962	FAST kinase domains 2	Fastkd2	2.747	0.08294
ENSMUSG00000028494	perilipin 2	Plin2	2.691	0
ENSMUSG00000027597	S-adenosylhomocysteine hydrolase	Ahcy	2.58	0
ENSMUSG00000052310	solute carrier family 39 (zinc transporter), member 1	Slc39a1	2.506	0.08294
ENSMUSG00000066232	importin 7	Ipo7	2.406	0.03759
ENSMUSG00000027359	solute carrier family 27 (fatty acid transporter), member 2	Slc27a2	2.391	0
ENSMUSG00000046434	heterogeneous nuclear ribonucleoprotein A1	Hnrnpa1	2.343	0.03808
ENSMUSG00000002944	CD36 molecule	Cd36	2.314	0.08294
ENSMUSG00000022407	adenylosuccinate lyase	Adsl	2.297	0.04483
ENSMUSG00000022336	eukaryotic translation initiation factor 3, subunit E	Eif3e	2.269	0.01426
ENSMUSG00000067194	eukaryotic translation initiation factor 1A, X-linked	Eif1ax	2.256	0.03759
ENSMUSG00000057113	nucleophosmin 1	Npm1	2.221	0.03808
ENSMUSG00000030214	phospholipase B domain containing 1	Plbd1	2.21	0.05228
ENSMUSG00000023571	C1q and tumor necrosis factor related 12	C1qtnf12	2.176	0.01426
ENSMUSG00000013662	ATPase family, AAA domain containing 1	Atad1	2.17	0.01993
ENSMUSG00000041438	UTP4 small subunit processome component	Utp4	2.168	0.01993
ENSMUSG00000026245	phenylalanyl-tRNA synthetase, beta subunit	Farsb	2.162	0.07685
ENSMUSG00000020464	polyribonucleotide nucleotidyltransferase 1	Pnpt1	2.135	0.05228
ENSMUSG00000026798	coenzyme Q4	Coq4	2.098	0.03808
ENSMUSG00000028655	major facilitator superfamily domain containing 2A	Mfsd2a	2.089	0.08294
ENSMUSG00000020089	pyrophosphatase (inorganic) 1	Ppa1	2.087	0
ENSMUSG00000028607	carnitine palmitoyltransferase 2	Cpt2	2.062	0
ENSMUSG00000033186	mitotic spindle organizing protein 1	Mzt1	2.062	0.03759
ENSMUSG00000021131	ERH mRNA splicing and mitosis factor	Erh	2.05	0.03808
ENSMUSG00000030793	PYD and CARD domain containing	Pycard	2.014	0.01993
ENSMUSG00000028383	hydroxysteroid dehydrogenase like 2	Hsdl2	2.011	0
ENSMUSG00000052833	SUMO1 activating enzyme subunit 1	Sae1	1.979	0.02404
ENSMUSG00000091721	GIMAP family P-loop NTPase domain containing 1	Gimd1	1.957	0.01993
ENSMUSG00000023074	motile sperm domain containing 1	Mospd1	1.948	0.08294
ENSMUSG00000026003	acyl-Coenzyme A dehydrogenase, long-chain	Acadl	1.948	0.08294
ENSMUSG00000015961	adenylosuccinate synthetase, non muscle	Adss	1.946	0.01993
ENSMUSG00000058558	ribosomal protein L5	Rpl5	1.944	0.03759
ENSMUSG00000034729	mitochondrial ribosomal protein S10	Mrps10	1.933	0.03759
ENSMUSG00000019891	discoidin, CUB and LCCL domain containing 1	Dcbld1	1.921	0.08294
ENSMUSG00000026005	ribulose-5-phosphate-3-epimerase	Rpe	1.92	0.03759
ENSMUSG00000003131	platelet-activating factor acetylhydrolase, isoform 1b, subunit 2	Pafah1b2	1.907	0.03759
ENSMUSG00000020078	VPS26 retromer complex component A	Vps26a	1.907	0.03808
ENSMUSG00000057561	eukaryotic translation initiation factor 1A	Eif1a	1.907	0.08294
ENSMUSG00000058355	ATP-binding cassette, sub-family E (OABP), member 1	Abce1	1.902	0.0961
ENSMUSG00000051391	tyrosine 3-monooxygenase/tryptophan 5-monooxygenase activation pro	Ywhag	1.896	0.01426
ENSMUSG00000037280	polypeptide N-acetylgalactosaminyltransferase 6	Galnt6	1.893	0.08294
ENSMUSG00000029250	polymerase (RNA) II (DNA directed) polypeptide B	Polr2b	1.888	0.03759
ENSMUSG00000010205	ribonucleoprotein, PTB-binding 1	Raver1	1.877	0.08294
ENSMUSG00000039835	NHS-like 1	Nhsl1	1.877	0.08294
ENSMUSG00000028156	eukaryotic translation initiation factor 4E	Eif4e	1.875	0.01426
ENSMUSG00000025613	chaperonin containing Tcp1, subunit 8 (theta)	Cct8	1.854	0.03759
ENSMUSG00000023452	phosphatidylserine decarboxylase	Pisd	1.853	0.03808
ENSMUSG00000006998	proteasome (prosome, macropain) 26S subunit, non-ATPase, 2	Psmd2	1.835	0.08294
ENSMUSG00000030357	FK506 binding protein 4	Fkbp4	1.833	0.03808
ENSMUSG00000022391	RAN GTPase activating protein 1	Rangap1	1.819	0.01993
ENSMUSG00000073792	asparagine-linked glycosylation 6 (alpha-1,3,-glucosyltransferase)	Alg6	1.811	0.05544
ENSMUSG00000093904	translocase of outer mitochondrial membrane 20	Tomm20	1.805	0.0628
ENSMUSG00000018848	arginyl-tRNA synthetase	Rars	1.801	0.01426
ENSMUSG00000034484	sorting nexin 2	Snx2	1.796	0.03808
ENSMUSG00000004451	v-ral simian leukemia viral oncogene B	Ralb	1.793	0.03759
ENSMUSG00000060373	heterogeneous nuclear ribonucleoprotein C	Hnrnpc	1.774	0.03808
ENSMUSG00000022241	threonyl-tRNA synthetase	Tars	1.772	0.04483
ENSMUSG00000006315	transmembrane protein 147	Tmem147	1.771	0.01426
ENSMUSG00000037601	NME/NM23 nucleoside diphosphate kinase 1	Nme1	1.727	0.01426
ENSMUSG00000079614	SEH1-like (S. cerevisiae	Seh1l	1.726	0.03808
ENSMUSG00000063576	kelch domain containing 3	Klhdc3	1.704	0.03759
ENSMUSG00000031197	von Hippel-Lindau binding protein 1	Vbp1	1.701	0.04483
ENSMUSG00000070372	capping protein (actin filament) muscle Z-line, alpha 1	Capza1	1.686	0.04483
ENSMUSG00000036275	RIKEN cDNA 9530068E07 gene	9530068E07Rik	1.677	0.04483
ENSMUSG00000036550	CCR4-NOT transcription complex, subunit 1	Cnot1	1.676	0.08294
ENSMUSG00000001056	NHP2 ribonucleoprotein	Nhp2	1.674	0.08294

ENSMUSG00000024900	carnitine palmitoyltransferase 1a, liver	Cpt1a	1.669	0
ENSMUSG00000031146	proteolipid protein 2	Plp2	1.667	0.03808
ENSMUSG00000031311	non-POU-domain-containing, octamer binding protein	Nono	1.665	0.03808
ENSMUSG00000030760	alkaline ceramidase 3	Acer3	1.664	0.03308
ENSMUSG00000028622	mitochondrial ribosomal protein L37	Mrpl37	1.655	0.08512
ENSMUSG00000041360	pumilio RNA-binding family member 3	Pum3	1.628	0.03808
ENSMUSG00000050043	thioredoxin-related transmembrane protein 2	Tmx2	1.623	0.04483
ENSMUSG00000020460	ribosomal protein S27A	Rps27a	1.621	0.08294
ENSMUSG00000059447	hydroxyacyl-Coenzyme A dehydrogenase/3-ketoacyl-Coenzyme A thiolas	Hadhb	1.604	0.01993
ENSMUSG00000040354	methionine-tRNA synthetase	Mars	1.602	0.08294
ENSMUSG00000038900	ribosomal protein L12	Rpl12	1.6	0.0961
ENSMUSG00000037470	UDP-glucose glycoprotein glucosyltransferase 1	Uggt1	1.594	0.08294
ENSMUSG00000047260	ER membrane protein complex subunit 6	Emc6	1.588	0
ENSMUSG00000025745	hydroxyacyl-Coenzyme A dehydrogenase/3-ketoacyl-Coenzyme A thiolas	Hadha	1.585	0.08294
ENSMUSG00000068039	t-complex protein 1	Tcp1	1.569	0.07129
ENSMUSG00000037742	eukaryotic translation elongation factor 1 alpha 1	Eef1a1	1.552	0.05228
ENSMUSG00000059811	atlastin GTPase 2	Atl2	1.544	0.01993
ENSMUSG00000024487	Yip1 domain family, member 5	Yipf5	1.535	0.03808
ENSMUSG00000021583	endoplasmic reticulum aminopeptidase 1	Erap1	1.523	0.03308
ENSMUSG00000028699	tetraspanin 1	Tspan1	1.507	0.01426
ENSMUSG00000007458	mannose-6-phosphate receptor, cation dependent	M6pr	1.507	0.08294
ENSMUSG00000041444	Rho GTPase activating protein 32	Arhgap32	1.505	0.03808
ENSMUSG00000027133	NOP10 ribonucleoprotein	Nop10	1.496	0.03808
ENSMUSG00000015357	caseinolytic mitochondrial matrix peptidase chaperone subunit	Clpx	1.495	0.08294
ENSMUSG00000022403	suppression of tumorigenicity 13	St13	1.493	0.03759
ENSMUSG00000053898	enoyl coenzyme A hydratase 1, peroxisomal	Ech1	1.487	0.01426
ENSMUSG00000040774	choline/ethanolaminephosphotransferase 1	Cept1	1.485	0.02404
ENSMUSG00000026750	proteasome (prosome, macropain) subunit, beta type 7	Psmb7	1.471	0.03759
ENSMUSG00000028676	serine/arginine-rich splicing factor 10	Srsf10	1.47	0.0961
ENSMUSG00000033793	ATPase, H+ transporting, lysosomal V1 subunit H	Atp6v1h	1.466	0.08962
ENSMUSG00000017721	phosphatidylinositol glycan anchor biosynthesis, class T	Pigt	1.462	0.03759
ENSMUSG00000063856	glutathione peroxidase 1	Gpx1	1.46	0.03759
ENSMUSG00000036687	transmembrane protein 184a	Tmem184a	1.456	0.01426
ENSMUSG00000090862	ribosomal protein S13	Rps13	1.456	0.02404
ENSMUSG00000019818	CD164 antigen	Cd164	1.456	0.04483
ENSMUSG00000058267	mitochondrial ribosomal protein S14	Mrps14	1.454	0.05077
ENSMUSG00000079435	ribosomal protein L36A	Rpl36a	1.444	0.03308
ENSMUSG00000028691	peroxiredoxin 1	Prdx1	1.444	0.08294
ENSMUSG00000006736	tetraspanin 31	Tspan31	1.437	0.01993
ENSMUSG00000021660	basic transcription factor 3	Btf3	1.432	0.04483
ENSMUSG00000025950	isocitrate dehydrogenase 1 (NADP+), soluble	Idh1	1.432	0.08294
ENSMUSG00000020629	acireductone dioxygenase 1	Adi1	1.431	0.08512
ENSMUSG00000059291	ribosomal protein L11	Rpl11	1.427	0.01426
ENSMUSG00000027195	hydroxysteroid (17-beta) dehydrogenase 12	Hsd17b12	1.426	0.03308
ENSMUSG00000027809	electron transferring flavoprotein, dehydrogenase	Etfdh	1.423	0.05403
ENSMUSG00000008668	ribosomal protein S18	Rps18	1.419	0.01426
ENSMUSG00000031701	DnaJ heat shock protein family (Hsp40) member A2	Dnaja2	1.417	0.03759
ENSMUSG00000060636	ribosomal protein L35A	Rpl35a	1.415	0.08294
ENSMUSG00000057278	small nuclear ribonucleoprotein polypeptide G	Snrpg	1.403	0.01993
ENSMUSG00000024740	damage specific DNA binding protein 1	Ddb1	1.395	0.01426
ENSMUSG00000026766	methylmalonic aciduria (cobalamin deficiency) cblD type, with homocyst	Mmadhc	1.392	0.08294
ENSMUSG00000055302	Morf4 family associated protein 1	Mrfap1	1.384	0.01993
ENSMUSG00000021109	hypoxia inducible factor 1, alpha subunit	Hif1a	1.378	0.05403
ENSMUSG00000032959	phosphatidylethanolamine binding protein 1	Pebp1	1.367	0.08512
ENSMUSG00000005610	eukaryotic translation initiation factor 4, gamma 2	Eif4g2	1.366	0.01426
ENSMUSG00000022285	tyrosine 3-monooxygenase/tryptophan 5-monooxygenase activation pro	Ywhaz	1.362	0.0961
ENSMUSG00000061315	nascent polypeptide-associated complex alpha polypeptide	Naca	1.357	0.08294
ENSMUSG00000009927	ribosomal protein S25	Rps25	1.345	0.08962
ENSMUSG00000028452	valosin containing protein	Vcp	1.344	0.03759
ENSMUSG00000029632	Ndufa4, mitochondrial complex associated	Ndufa4	1.31	0.01993
ENSMUSG00000047187	RAB2A, member RAS oncogene family	Rab2a	1.305	0.08294
ENSMUSG00000015759	cornichon family AMPA receptor auxiliary protein 1	Cnih1	1.296	0.08512
ENSMUSG00000031980	angiotensinogen (serpin peptidase inhibitor, clade A, member 8)	Agt	1.269	0.08294
ENSMUSG00000020048	heat shock protein 90, beta (Grp94), member 1	Hsp90b1	1.265	0.03759
ENSMUSG00000062070	phosphoglycerate kinase 1	Pgk1	1.263	0.05544
ENSMUSG00000030663	RIKEN cDNA 1110004F10 gene	1110004F10Rik	1.24	0.03808
ENSMUSG00000076432	tyrosine 3-monooxygenase/tryptophan 5-monooxygenase activation pro	Ywhaq	1.233	0.03808
ENSMUSG00000020849	tyrosine 3-monooxygenase/tryptophan 5-monooxygenase activation pro	Ywhae	1.233	0.06546
ENSMUSG00000007041	chloride intracellular channel 1	Clic1	0.854	0.08494
ENSMUSG00000033335	dynamin 2	Dnm2	0.835	0.09628
ENSMUSG00000031950	gamma-aminobutyric acid (GABA) A receptor-associated protein-like 2	Gabarapl2	0.827	0.05606
ENSMUSG00000032580	RNA binding motif protein 5	Rbm5	0.827	0.10287
ENSMUSG00000050732	vesicle-associated membrane protein 8	Vamp8	0.825	0.05606
ENSMUSG00000019505	ubiquitin B	Ubb	0.808	0.10287
ENSMUSG00000073420	butyrophilin-like 5, pseudogene	Btnl5-ps	0.789	0.10287
ENSMUSG00000042613	pre B cell leukemia transcription factor interacting protein 1	Pbxip1	0.788	0.07024
ENSMUSG00000095041		AC149090.1	0.781	0.10287
ENSMUSG00000026175	villin 1	Vil1	0.776	0.09715
ENSMUSG00000000915	huntingtin interacting protein 1 related	Hip1r	0.775	0.10287
ENSMUSG00000040943	tet methylcytosine dioxygenase 2	Tet2	0.77	0.10287
ENSMUSG00000022141	NIPBL cohesin loading factor	Nipbl	0.763	0.10287
ENSMUSG00000090231	complement factor B	Cfb	0.754	0.05606
ENSMUSG00000035545	leukocyte receptor cluster (LRC) member 8	Leng8	0.753	0.09628
ENSMUSG00000024661	ferritin heavy polypeptide 1	Fth1	0.744	0.03955
ENSMUSG00000033159	cyclin Pas1/PHO80 domain containing 1	Cnppd1	0.743	0.09317

ENSMUSG00000090841	myosin, light polypeptide 6, alkali, smooth muscle and non-muscle	Myl6	0.742	0.08936
ENSMUSG00000024772	EH-domain containing 1	Ehd1	0.739	0.10287
ENSMUSG00000033916	charged multivesicular body protein 2A	Chmp2a	0.737	0.06574
ENSMUSG00000022175	low-density lipoprotein receptor-related protein 10	Lrp10	0.733	0.07024
ENSMUSG00000024953	peroxiredoxin 5	Prdx5	0.732	0
ENSMUSG00000030172	ELKS/RAB6-interacting/CAST family member 1	Erc1	0.731	0.06132
ENSMUSG00000059323	tonsoku-like, DNA repair protein	Tonsl	0.728	0.06574
ENSMUSG00000028064	sema domain, immunoglobulin domain (Ig), transmembrane domain (TM	Sema4a	0.721	0.06132
ENSMUSG00000074733	zinc finger protein 950	Zfp950	0.721	0.08353
ENSMUSG00000038235	F11 receptor	F11r	0.719	0.10287
ENSMUSG00000047909	ankyrin repeat domain 16	Ankrd16	0.714	0.09628
ENSMUSG00000025504	EPS8-like 2	Eps8l2	0.709	0.06132
ENSMUSG00000036073	galactose-1-phosphate uridyl transferase	Galt	0.707	0.05606
ENSMUSG00000028673	fucosidase, alpha-L- 1, tissue	Fuca1	0.705	0
ENSMUSG00000034647	ankyrin repeat domain 12	Ankrd12	0.7	0.06574
ENSMUSG00000020451	LIM motif-containing protein kinase 2	Limk2	0.697	0.10287
ENSMUSG00000041890	G protein-coupled receptor kinase-interactor 2	Git2	0.695	0.09715
ENSMUSG00000060601	nuclear receptor subfamily 1, group H, member 2	Nr1h2	0.694	0.03969
ENSMUSG00000028821	SYF2 homolog, RNA splicing factor (S. cerevisiae)	Syf2	0.694	0.05606
ENSMUSG00000025337	SBDS ribosome maturation factor	Sbds	0.694	0.08494
ENSMUSG00000036093	ADP-ribosylation factor-like 5A	Arl5a	0.693	0.02506
ENSMUSG00000006095	tubulin folding cofactor B	Tbcb	0.693	0.05606
ENSMUSG00000025207	sema domain, immunoglobulin domain (Ig), transmembrane domain (TM	Sema4g	0.691	0.08353
ENSMUSG00000006395	hydroxypyruvate isomerase (putative)	Hyi	0.686	0.06132
ENSMUSG00000027881	PRP38 pre-mRNA processing factor 38 (yeast) domain containing B	Prpf38b	0.682	0.06132
ENSMUSG00000022971	interferon (alpha and beta) receptor 2	Ifnar2	0.681	0.06574
ENSMUSG00000024187	family with sequence similarity 234, member A	Fam234a	0.679	0
ENSMUSG00000022617	choline kinase beta	Chkb	0.679	0.06132
ENSMUSG00000022472	desumoylating isopeptidase 1	Desi1	0.678	0.08353
ENSMUSG00000028251	thiosulfate sulfurtransferase (rhodanese)-like domain containing 3	Tstd3	0.677	0.10287
ENSMUSG00000085042	abhydrolase domain containing 11, opposite strand	Abhd11os	0.676	0.07024
ENSMUSG00000041936	agrin	Agrn	0.674	0.07801
ENSMUSG00000037999	ArfGAP with RhoGAP domain, ankyrin repeat and PH domain 2	Arap2	0.671	0.09715
ENSMUSG00000002395	unconventional SNARE in the ER 1 homolog (S. cerevisiae)	Use1	0.667	0.0341
ENSMUSG00000038256	B cell CLL/lymphoma 9	Bcl9	0.666	0.07801
ENSMUSG00000106106		CT010467.1	0.665	0.09715
ENSMUSG00000006445	Eph receptor A2	Epha2	0.665	0.10287
ENSMUSG00000061751	kalirin, RhoGEF kinase	Kalrn	0.658	0.03955
ENSMUSG00000054499	death effector domain-containing DNA binding protein 2	Dedd2	0.658	0.06132
ENSMUSG00000039611	transmembrane protein 246	Tmem246	0.656	0.10287
ENSMUSG00000040600	EPS8-like 3	Eps8l3	0.654	0.02506
ENSMUSG00000028465	talin 1	Tln1	0.654	0.07801
ENSMUSG00000005621	zinc finger protein 592	Zfp592	0.652	0.10287
ENSMUSG00000022453	N-acetyl galactosaminidase, alpha	Naga	0.651	0.08353
ENSMUSG00000000823	zinc finger protein 512B	Zfp512b	0.649	0.08353
ENSMUSG00000004864	mitogen-activated protein kinase 13	Mapk13	0.647	0.02506
ENSMUSG00000020733	solute carrier family 9 (sodium/hydrogen exchanger), member 3 regulato	Slc9a3r1	0.647	0.02667
ENSMUSG00000022564	glutamate receptor, ionotropic, N-methyl D-aspartate-associated protein	Grina	0.647	0.03955
ENSMUSG00000073889	interleukin 11 receptor, alpha chain 1	Il11ra1	0.644	0.10287
ENSMUSG00000020644	inhibitor of DNA binding 2	Id2	0.643	0.06132
ENSMUSG00000038766	GA repeat binding protein, beta 2	Gabpb2	0.643	0.09715
ENSMUSG00000020115	TANK-binding kinase 1	Tbk1	0.641	0.09317
ENSMUSG00000041263	RUN and SH3 domain containing 1	Rusc1	0.641	0.10287
ENSMUSG00000058056	palladin, cytoskeletal associated protein	Palld	0.639	0.06132
ENSMUSG00000001672	MARVEL (membrane-associating) domain containing 3	Marveld3	0.638	0.10287
ENSMUSG00000055013	ArfGAP with GTPase domain, ankyrin repeat and PH domain 1	Agap1	0.633	0.07575
ENSMUSG00000052512	neuron navigator 2	Nav2	0.629	0.06132
ENSMUSG00000062352	integrin beta 1 binding protein 1	Itgb1bp1	0.627	0.05606
ENSMUSG00000020827	misshapen-like kinase 1 (zebrafish)	Mink1	0.626	0.07801
ENSMUSG00000067212	histocompatibility 2, T region locus 23	H2-T23	0.625	0
ENSMUSG00000026349	cyclin T2	Ccnt2	0.625	0.07801
ENSMUSG00000071654	ubiquinol-cytochrome c reductase complex assembly factor 3	Uqcc3	0.623	0.06132
ENSMUSG00000003746	mannosidase 1, alpha	Man1a	0.621	0.08353
ENSMUSG00000024371	complement component 2 (within H-2S)	C2	0.617	0.01034
ENSMUSG00000014504	signal recognition particle 19	Srp19	0.616	0.0341
ENSMUSG00000000339	RNA 3'-terminal phosphate cyclase	Rtca	0.615	0.06132
ENSMUSG00000038708	golgi autoantigen, golgin subfamily a, 4	Golga4	0.615	0.07024
ENSMUSG00000030824	nucleobindin 1	Nucb1	0.613	0.06132
ENSMUSG00000032583	MON1 homolog A, secretory traffciking associated	Mon1a	0.613	0.06132
ENSMUSG00000041354	ral guanine nucleotide dissociation stimulator-like 2	Rgl2	0.613	0.09317
ENSMUSG00000026576	ATPase, Na+/K+ transporting, beta 1 polypeptide	Atp1b1	0.611	0
ENSMUSG00000041757	pleckstrin homology domain containing, family A member 6	Plekha6	0.61	0.05606
ENSMUSG00000013787	euchromatic histone lysine N-methyltransferase 2	Ehmt2	0.608	0.10287
ENSMUSG00000037993	DEAH (Asp-Glu-Ala-His) box polypeptide 38	Dhx38	0.608	0.10287
ENSMUSG00000042682	selenoprotein K	Selenok	0.607	0.0341
ENSMUSG00000022994	adenylate cyclase 6	Adcy6	0.603	0
ENSMUSG00000048249	CREB3 regulatory factor	Crebrf	0.6	0.09715
ENSMUSG00000039431	myotubularin related protein 7	Mtmr7	0.597	0.10287
ENSMUSG00000037234	hook microtubule tethering protein 3	Hook3	0.596	0.06132
ENSMUSG00000040033	signal transducer and activator of transcription 2	Stat2	0.596	0.06132
ENSMUSG00000019362	DNA segment, Chr 8, ERATO Doi 738, expressed	D8Ertd738e	0.595	0.07024
ENSMUSG00000004565	patatin-like phospholipase domain containing 6	Pnpla6	0.594	0.04893
ENSMUSG00000038618	Ras association (RalGDS/AF-6) domain family (N-terminal) member 7	Rassf7	0.593	0.03955
ENSMUSG00000002227	Moloney leukemia virus 10	Mov10	0.59	0.09628
ENSMUSG00000072812	AHNAK nucleoprotein 2	Ahnak2	0.586	0.0341
