# Supplementary material for: Small Intestinal Tuft Cell Activity Associates With Energy Metabolism in Diet-Induced Obesity
Source: Front Immunol. 2021 May 28;12:629391. doi: 10.3389/fimmu.2021.629391 (PMC8195285; doi:10.3389/fimmu.2021.629391)
Supplement: Supplementary file 3 [file DataSheet_3.pdf]

Supplementary data 2: List of differently regulated pathways in small intestinal tuft cells under HFD-feeding vs RFD-feeding conditions for 9 weeks.

PathwayName	PathwayID	HFD/RFD (fold change)_9 wks	*q-value*_ 9 wks
Metal sequestration by antimicrobial proteins	R-MMU-6799990	0	0.02949
Phase 1 - inactivation of fast Na+ channels	R-MMU-5576894	0	0.02949
TWIK-releated acid-sensitive K+ channel (TASK)	R-MMU-1299316	0	0.03876
Prostanoid ligand receptors	R-MMU-391908	0	0.06189
Adrenaline signalling through Alpha-2 adrenergic recep	R-MMU-392023	0	0.06592
CD28 co-stimulation	R-MMU-389356	0	0.07723
Digestion of dietary carbohydrate	R-MMU-189085	0	0.08057
Adrenoceptors	R-MMU-390696	0	0.08557
Proton-coupled neutral amino acid transporters	R-MMU-428559	0	0.09349
Signaling by Insulin receptor	R-MMU-74752	0.075	0.03876
VEGF binds to VEGFR leading to receptor dimerization	R-MMU-195399	0.103	0.09349
Activated NTRK3 signals through PI3K	R-MMU-9603381	0.13	0.04809
AKT-mediated inactivation of FOXO1A	R-MMU-211163	0.147	0.09349
IRS activation	R-MMU-74713	0.188	0.03531
GABA A receptor activation	R-MMU-977441	0.232	0.03592
Synthesis of PIPs in the nucleus	R-MMU-8847453	0.253	0.03592
Phospholipase C-mediated cascade: FGFR1	R-MMU-5654219	0.281	0.05347
Role of ABL in ROBO-SLIT signaling	R-MMU-428890	0.297	0.0705
Invadopodia formation	R-MMU-8941237	0.301	0.0705
The NLRP1 inflammasome	R-MMU-844455	0.302	0.03592
Proton/oligopeptide cotransporters	R-MMU-427975	0.303	0.08557
Interleukin-18 signaling	R-MMU-9012546	0.311	0.03876
Activation of Matrix Metalloproteinases	R-MMU-1592389	0.336	0.08557
Antimicrobial peptides	R-MMU-6803157	0.345	0.02949
ARMS-mediated activation	R-MMU-170984	0.361	0.03592
Class C/3 (Metabotropic glutamate/pheromone recept	R-MMU-420499	0.391	0.08057
Phospholipase C-mediated cascade; FGFR4	R-MMU-5654228	0.394	0.05347
Metabolism of ingested SeMet, Sec, MeSec into H2Se	R-MMU-2408508	0.395	0.02949
DAP12 interactions	R-MMU-2172127	0.405	0.04833
RUNX3 regulates YAP1-mediated transcription	R-MMU-8951671	0.412	0.03113
Endosomal/Vacuolar pathway	R-MMU-1236977	0.413	0.02949
Reversal of alkylation damage by DNA dioxygenases	R-MMU-73943	0.417	0.03876
Interaction With The Zona Pellucida	R-MMU-1300644	0.42	0.02949
NGF processing	R-MMU-167060	0.426	0.03531
Negative regulation of TCF-dependent signaling by WN	R-MMU-3772470	0.441	0.08057
ER-Phagosome pathway	R-MMU-1236974	0.446	0.02949
Phase 4 - resting membrane potential	R-MMU-5576886	0.448	0.04809
G2 Phase	R-MMU-68911	0.456	0.05292
CREB1 phosphorylation through the activation of Aden	R-MMU-442720	0.457	0.05691
c-src mediated regulation of Cx43 function and closure	R-MMU-191647	0.467	0.03592
LGI-ADAM interactions	R-MMU-5682910	0.476	0.08557
Phospholipase C-mediated cascade; FGFR2	R-MMU-5654221	0.484	0.03876
Nucleobase catabolism	R-MMU-8956319	0.493	0.03876
Lysosomal oligosaccharide catabolism	R-MMU-8853383	0.498	0.05963
SEMA3A-Plexin repulsion signaling by inhibiting Integri	R-MMU-399955	0.503	0.03876
DAG and IP3 signaling	R-MMU-1489509	0.513	0.02949
Post-transcriptional silencing by small RNAs	R-MMU-426496	0.513	0.04809
L1CAM interactions	R-MMU-373760	0.515	0.03876
Acetylcholine Neurotransmitter Release Cycle	R-MMU-264642	0.516	0.03113
GRB2:SOS provides linkage to MAPK signaling for Integ	R-MMU-354194	0.524	0.02949
The activation of arylsulfatases	R-MMU-1663150	0.524	0.03876
Signaling by EGFR	R-MMU-177929	0.528	0.05249
Glycerophospholipid catabolism	R-MMU-6814848	0.533	0.04833
Antigen Presentation: Folding, assembly and peptide lo	R-MMU-983170	0.535	0.02949
p130Cas linkage to MAPK signaling for integrins	R-MMU-372708	0.537	0.02949
SOS-mediated signalling	R-MMU-112412	0.54	0.071

CS/DS degradation	R-MMU-2024101	0.545	0.03876
Transport of nucleosides and free purine and pyrimidir	R-MMU-83936	0.548	0.03592
Chondroitin sulfate biosynthesis	R-MMU-2022870	0.549	0.08057
HS-GAG degradation	R-MMU-2024096	0.552	0.05347
Class A/1 (Rhodopsin-like receptors)	R-MMU-373076	0.552	0.08557
TP53 Regulates Transcription of Death Receptors and L	R-MMU-6803211	0.56	0.05347
Inhibition of TSC complex formation by PKB	R-MMU-165181	0.563	0.08557
FGFR4 ligand binding and activation	R-MMU-190322	0.567	0.07075
RSK activation	R-MMU-444257	0.569	0.06189
RUNX3 regulates CDKN1A transcription	R-MMU-8941855	0.584	0.04833
N-glycan trimming and elongation in the cis-Golgi	R-MMU-964739	0.587	0.05347
Negative regulation of the PI3K/AKT network	R-MMU-199418	0.589	0.04833
TFAP2 (AP-2) family regulates transcription of cell cycle	R-MMU-8866911	0.592	0.08057
Synthesis of bile acids and bile salts via 27-hydroxycho	R-MMU-193807	0.596	0.08557
Synthesis of PI	R-MMU-1483226	0.597	0.03113
Voltage gated Potassium channels	R-MMU-1296072	0.597	0.07723
DEx/H-box helicases activate type I IFN and inflammato	R-MMU-3134963	0.6	0.03876
Response to elevated platelet cytosolic Ca2+	R-MMU-76005	0.6	0.07723
YAP1- and WWTR1 (TAZ)-stimulated gene expression	R-MMU-2032785	0.601	0.08557
Progressive trimming of alpha-1,2-linked mannose resi	R-MMU-964827	0.606	0.03531
Termination of O-glycan biosynthesis	R-MMU-977068	0.606	0.03592
Signaling by ERBB4	R-MMU-1236394	0.613	0.08557
DSCAM interactions	R-MMU-376172	0.619	0.03876
TICAM1-dependent activation of IRF3/IRF7	R-MMU-9013973	0.621	0.04833
Synthesis of PG	R-MMU-1483148	0.621	0.07723
Fibronectin matrix formation	R-MMU-1566977	0.624	0.04809
Mitochondrial calcium ion transport	R-MMU-8949215	0.633	0.02949
TRAF6 mediated IRF7 activation	R-MMU-933541	0.634	0.03592
TRAF3-dependent IRF activation pathway	R-MMU-918233	0.634	0.04809
Degradation of the extracellular matrix	R-MMU-1474228	0.639	0.03876
Nuclear signaling by ERBB4	R-MMU-1251985	0.639	0.08557
p53-Dependent G1 DNA Damage Response	R-MMU-69563	0.641	0.08057
MyD88 dependent cascade initiated on endosome	R-MMU-975155	0.643	0.03876
Tight junction interactions	R-MMU-420029	0.647	0.02949
GAB1 signalosome	R-MMU-180292	0.648	0.02949
Nicotinamide salvaging	R-MMU-197264	0.653	0.02949
Thyroxine biosynthesis	R-MMU-209968	0.653	0.05347
RUNX1 interacts with co-factors whose precise effect c	R-MMU-8939243	0.658	0.03113
PI3K Cascade	R-MMU-109704	0.66	0.03876
Formyl peptide receptors bind formyl peptides and ma	R-MMU-444473	0.663	0.05963
RUNX3 regulates p14-ARF	R-MMU-8951936	0.665	0.0884
Noncanonical activation of NOTCH3	R-MMU-9017802	0.666	0.08057
Reactions specific to the complex N-glycan synthesis pa	R-MMU-975578	0.667	0.03531
RUNX1 and FOXP3 control the development of regulato	R-MMU-8877330	0.667	0.03876
Integrin alphaIIb beta3 signaling	R-MMU-354192	0.668	0.02949
PI3K events in ERBB2 signaling	R-MMU-1963642	0.671	0.09349
Cyclin A:Cdk2-associated events at S phase entry	R-MMU-69656	0.672	0.02949
EGFR interacts with phospholipase C-gamma	R-MMU-212718	0.675	0.071
Proline catabolism	R-MMU-70688	0.676	0.03876
PI5P Regulates TP53 Acetylation	R-MMU-6811555	0.682	0.071
RUNX1 regulates estrogen receptor mediated transcrip	R-MMU-8931987	0.683	0.03876
Regulation of RUNX1 Expression and Activity	R-MMU-8934593	0.686	0.02949
RUNX1 regulates transcription of genes involved in inte	R-MMU-8939247	0.686	0.09605
FGFR2c ligand binding and activation	R-MMU-190375	0.688	0.05249
Lysosphingolipid and LPA receptors	R-MMU-419408	0.689	0.06088
Receptor-type tyrosine-protein phosphatases	R-MMU-388844	0.69	0.03592
GRB7 events in ERBB2 signaling	R-MMU-1306955	0.694	0.0884
C6 deamination of adenosine	R-MMU-75102	0.695	0.02949
Formation of editosomes by ADAR proteins	R-MMU-77042	0.695	0.02949
AKT phosphorylates targets in the nucleus	R-MMU-198693	0.7	0.08057

FRS-mediated FGFR3 signaling	R-MMU-5654706	0.705	0.09349
Regulation of FOXO transcriptional activity by acetylati	R-MMU-9617629	0.706	0.08057
TNFs bind their physiological receptors	R-MMU-5669034	0.709	0.02949
MET receptor recycling	R-MMU-8875656	0.709	0.03592
Gastrin-CREB signalling pathway via PKC and MAPK	R-MMU-881907	0.714	0.05347
Growth hormone receptor signaling	R-MMU-982772	0.714	0.06088
Synthesis of PIPs at the late endosome membrane	R-MMU-1660517	0.718	0.09605
RUNX1 regulates transcription of genes involved in diff	R-MMU-8939246	0.721	0.04833
HS-GAG biosynthesis	R-MMU-2022928	0.721	0.0705
Interleukin-27 signaling	R-MMU-9020956	0.723	0.05249
Interleukin-35 Signalling	R-MMU-8984722	0.723	0.05963
Cyclin E associated events during G1/S transition	R-MMU-69202	0.728	0.05347
Frs2-mediated activation	R-MMU-170968	0.728	0.08057
Synaptic adhesion-like molecules	R-MMU-8849932	0.729	0.03592
Serotonin Neurotransmitter Release Cycle	R-MMU-181429	0.729	0.05249
Triglyceride biosynthesis	R-MMU-75109	0.729	0.05249
Xenobiotics	R-MMU-211981	0.729	0.0705
Formation of the cornified envelope	R-MMU-6809371	0.729	0.071
Keratinization	R-MMU-6805567	0.729	0.07723
Interleukin-20 family signaling	R-MMU-8854691	0.731	0.02949
Interleukin-9 signaling	R-MMU-8985947	0.733	0.06592
TRAF6 mediated induction of NFkB and MAP kinases u	R-MMU-975138	0.734	0.08057
IRS-mediated signalling	R-MMU-112399	0.736	0.07723
Synthesis of bile acids and bile salts	R-MMU-192105	0.736	0.08057
Phosphorylation of proteins involved in G1/S transition	R-MMU-69200	0.737	0.05249
Glycerophospholipid biosynthesis	R-MMU-1483206	0.738	0.03876
Elastic fibre formation	R-MMU-1566948	0.739	0.07723
Synthesis of pyrophosphates in the cytosol	R-MMU-1855167	0.743	0.0884
Laminin interactions	R-MMU-3000157	0.744	0.0705
GRB2 events in ERBB2 signaling	R-MMU-1963640	0.745	0.08057
Phosphorylation of CD3 and TCR zeta chains	R-MMU-202427	0.746	0.04809
ERBB2 Activates PTK6 Signaling	R-MMU-8847993	0.751	0.08557
Basigin interactions	R-MMU-210991	0.754	0.04809
Amino acid transport across the plasma membrane	R-MMU-352230	0.754	0.04833
TGF-beta receptor signaling in EMT (epithelial to mese	R-MMU-2173791	0.756	0.05347
MET Receptor Activation	R-MMU-6806942	0.762	0.05347
Repression of WNT target genes	R-MMU-4641265	0.762	0.09605
O-linked glycosylation of mucins	R-MMU-913709	0.764	0.02949
Toll Like Receptor 9 (TLR9) Cascade	R-MMU-168138	0.766	0.0705
A tetrasaccharide linker sequence is required for GAG s	R-MMU-1971475	0.766	0.09349
Integrin cell surface interactions	R-MMU-216083	0.767	0.03592
Caspase-mediated cleavage of cytoskeletal proteins	R-MMU-264870	0.767	0.0705
Transport and synthesis of PAPS	R-MMU-174362	0.767	0.09605
SHC1 events in ERBB2 signaling	R-MMU-1250196	0.768	0.09605
Synthesis of bile acids and bile salts via 7alpha-hydroxy	R-MMU-193368	0.769	0.09349
Termination of translesion DNA synthesis	R-MMU-5656169	0.77	0.04833
TRAF6 mediated NF-kB activation	R-MMU-933542	0.771	0.04809
Syndecan interactions	R-MMU-3000170	0.771	0.08557
NF-kB is activated and signals survival	R-MMU-209560	0.772	0.05347
Proton-coupled monocarboxylate transport	R-MMU-433692	0.772	0.071
MET activates RAP1 and RAC1	R-MMU-8875555	0.773	0.07723
Norepinephrine Neurotransmitter Release Cycle	R-MMU-181430	0.777	0.071
Purine salvage	R-MMU-74217	0.78	0.03876
RIP-mediated NFkB activation via ZBP1	R-MMU-1810476	0.781	0.03876
Digestion	R-MMU-8935690	0.781	0.07723
Negative regulators of DDX58/IFIH1 signaling	R-MMU-936440	0.787	0.06592
Glutamate Neurotransmitter Release Cycle	R-MMU-210500	0.787	0.08057
EPH-Ephrin signaling	R-MMU-2682334	0.79	0.06189
Regulation of FZD by ubiquitination	R-MMU-4641263	0.791	0.04809
Sialic acid metabolism	R-MMU-4085001	0.791	0.05691

WNT mediated activation of DVL	R-MMU-201688	0.795	0.09732
CD209 (DC-SIGN) signaling	R-MMU-5621575	0.797	0.03592
TNFR1-induced proapoptotic signaling	R-MMU-5357786	0.801	0.05691
Formation of the beta-catenin:TCF transactivating com	R-MMU-201722	0.801	0.05691
Regulation of TP53 Degradation	R-MMU-6804757	0.802	0.05292
Synthesis of PC	R-MMU-1483191	0.802	0.06088
Tie2 Signaling	R-MMU-210993	0.804	0.08057
VEGFR2 mediated cell proliferation	R-MMU-5218921	0.804	0.09349
CREB phosphorylation	R-MMU-199920	0.806	0.09349
NCAM signaling for neurite out-growth	R-MMU-375165	0.807	0.03592
CD28 dependent PI3K/Akt signaling	R-MMU-389357	0.807	0.04809
Organic cation transport	R-MMU-549127	0.807	0.04809
RET signaling	R-MMU-8853659	0.807	0.08557
Nuclear Receptor transcription pathway	R-MMU-383280	0.808	0.05347
IRS-related events triggered by IGF1R	R-MMU-2428928	0.809	0.07723
EPH-ephrin mediated repulsion of cells	R-MMU-3928665	0.809	0.08557
RUNX3 regulates NOTCH signaling	R-MMU-8941856	0.811	0.09349
Degradation of GLI1 by the proteasome	R-MMU-5610780	0.812	0.06592
Phase 2 - plateau phase	R-MMU-5576893	0.813	0.071
Activation of NF-kappaB in B cells	R-MMU-1169091	0.813	0.08057
Translesion synthesis by REV1	R-MMU-110312	0.814	0.07723
Translesion synthesis by POLI	R-MMU-5656121	0.814	0.09349
cGMP effects	R-MMU-418457	0.815	0.09349
CREB3 factors activate genes	R-MMU-8874211	0.818	0.08557
VLDLR internalisation and degradation	R-MMU-8866427	0.819	0.08557
Signal attenuation	R-MMU-74749	0.821	0.0705
Regulation of TNFR1 signaling	R-MMU-5357905	0.822	0.05249
N-Glycan antennae elongation	R-MMU-975577	0.823	0.03876
Interleukin-12 signaling	R-MMU-9020591	0.823	0.09349
PTK6 Regulates RTKs and Their Effectors AKT1 and DOK	R-MMU-8849469	0.826	0.0705
Type I hemidesmosome assembly	R-MMU-446107	0.826	0.08057
TRAF6-mediated induction of TAK1 complex within TLF	R-MMU-937072	0.827	0.0705
IRAK2 mediated activation of TAK1 complex upon TLR7	R-MMU-975163	0.827	0.07723
ABC transporters in lipid homeostasis	R-MMU-1369062	0.827	0.08557
IRAK2 mediated activation of TAK1 complex	R-MMU-937042	0.828	0.06088
ERK/MAPK targets	R-MMU-198753	0.83	0.03876
Regulation of lipid metabolism by Peroxisome prolifera	R-MMU-400206	0.831	0.05249
Telomere C-strand (Lagging Strand) Synthesis	R-MMU-174417	0.831	0.08057
BH3-only proteins associate with and inactivate anti-ap	R-MMU-111453	0.833	0.04809
Asymmetric localization of PCP proteins	R-MMU-4608870	0.833	0.08557
Cholesterol biosynthesis via desmosterol	R-MMU-6807047	0.833	0.09349
RUNX1 regulates transcription of genes involved in diff	R-MMU-8939236	0.834	0.03876
Nectin/Necl trans heterodimerization	R-MMU-420597	0.834	0.06592
Other interleukin signaling	R-MMU-449836	0.834	0.07723
Regulation of IFNG signaling	R-MMU-877312	0.834	0.07723
Synthesis of PA	R-MMU-1483166	0.835	0.04833
NOSTRIN mediated eNOS trafficking	R-MMU-203641	0.835	0.06592
Downregulation of ERBB2:ERBB3 signaling	R-MMU-1358803	0.839	0.0884
Negative regulation of FGFR1 signaling	R-MMU-5654726	0.84	0.03876
PKMTs methylate histone lysines	R-MMU-3214841	0.84	0.08557
Activated NOTCH1 Transmits Signal to the Nucleus	R-MMU-2122948	0.842	0.08057
Negative regulation of FGFR2 signaling	R-MMU-5654727	0.845	0.0705
Negative regulation of FGFR3 signaling	R-MMU-5654732	0.848	0.06592
Negative regulation of FGFR4 signaling	R-MMU-5654733	0.848	0.07723
TNFR1-induced NFkappaB signaling pathway	R-MMU-5357956	0.849	0.0705
Hedgehog 'on' state	R-MMU-5632684	0.851	0.0792
LDL clearance	R-MMU-8964038	0.852	0.03876
Degradation of DVL	R-MMU-4641258	0.852	0.08057
Regulation of RAS by GAPs	R-MMU-5658442	0.853	0.05691
Activation of IRF3/IRF7 mediated by TBK1/IKK epsilon	R-MMU-936964	0.853	0.071

TP53 Regulates Transcription of DNA Repair Genes	R-MMU-6796648	0.855	0.0705
Gap-filling DNA repair synthesis and ligation in GG-NEF	R-MMU-5696397	0.856	0.0705
Interleukin-6 signaling	R-MMU-1059683	0.858	0.0884
Downregulation of TGF-beta receptor signaling	R-MMU-2173788	0.863	0.07075
Spry regulation of FGF signaling	R-MMU-1295596	0.865	0.08557
Disassembly of the destruction complex and recruitme	R-MMU-4641262	0.866	0.08557
RNA Polymerase II Transcription Elongation	R-MMU-75955	0.868	0.071
TAK1 activates NFkB by phosphorylation and activatior	R-MMU-445989	0.869	0.08557
Ubiquitin Mediated Degradation of Phosphorylated Cd	R-MMU-69601	0.88	0.0884
Negative regulation of MET activity	R-MMU-6807004	0.881	0.06361
Regulation of TLR by endogenous ligand	R-MMU-5686938	0.882	0.08755
CDK-mediated phosphorylation and removal of Cdc6	R-MMU-69017	0.885	0.05347
Degradation of AXIN	R-MMU-4641257	0.885	0.07723
Formation of RNA Pol II elongation complex	R-MMU-112382	0.887	0.07075
TNF signaling	R-MMU-75893	0.889	0.08557
APC/C:Cdc20 mediated degradation of Securin	R-MMU-174154	0.891	0.0705
Activation of G protein gated Potassium channels	R-MMU-1296041	0.899	0.07723
Inhibition of voltage gated Ca2+ channels via Gbeta/ga	R-MMU-997272	0.899	0.07723
Regulation of TP53 Activity through Acetylation	R-MMU-6804758	0.9	0.08557
APC/C:Cdc20 mediated degradation of Cyclin B	R-MMU-174048	0.904	0.08057
Macroautophagy	R-MMU-1632852	0.908	0.03876
Phospholipase C-mediated cascade; FGFR3	R-MMU-5654227	0.91	0.08557
SMAD2/SMAD3:SMAD4 heterotrimer regulates transcı	R-MMU-2173796	0.913	0.06592
Downstream signal transduction	R-MMU-186763	0.921	0.07723
VxPx cargo-targeting to cilium	R-MMU-5620916	0.926	0.0884
Processing of DNA double-strand break ends	R-MMU-5693607	1.047	0.03269
ABC-family proteins mediated transport	R-MMU-382556	1.056	0.03467
Sphingolipid de novo biosynthesis	R-MMU-1660661	1.073	0.04169
MAPK6/MAPK4 signaling	R-MMU-5687128	1.073	0.0631
Neddylation	R-MMU-8951664	1.088	0.02847
Mitochondrial iron-sulfur cluster biogenesis	R-MMU-1362409	1.092	0.0427
E3 ubiquitin ligases ubiquitinate target proteins	R-MMU-8866654	1.106	0.03097
EPHB-mediated forward signaling	R-MMU-3928662	1.113	0.03269
Ion transport by P-type ATPases	R-MMU-936837	1.116	0.05206
IKK complex recruitment mediated by RIP1	R-MMU-937041	1.119	0.02016
RHO GTPases Activate WASPs and WAVEs	R-MMU-5663213	1.119	0.04791
Acyl chain remodelling of PE	R-MMU-1482839	1.138	0.0431
Cdc20:Phospho-APC/C mediated degradation of Cyclin	R-MMU-174184	1.142	0.0431
UCH proteinases	R-MMU-5689603	1.147	0.00453
Golgi Cisternae Pericentriolar Stack Reorganization	R-MMU-162658	1.152	0.04169
SUMOylation of intracellular receptors	R-MMU-4090294	1.157	0.00849
Formation of TC-NER Pre-Incision Complex	R-MMU-6781823	1.16	0.00453
Prolactin receptor signaling	R-MMU-1170546	1.164	0.01158
VEGFA-VEGFR2 Pathway	R-MMU-4420097	1.167	0.04791
mRNA Splicing - Major Pathway	R-MMU-72163	1.187	0.01158
APC-Cdc20 mediated degradation of Nek2A	R-MMU-179409	1.187	0.01494
Acyl chain remodelling of PC	R-MMU-1482788	1.188	0.02847
COPII-mediated vesicle transport	R-MMU-204005	1.189	0.0427
RMTs methylate histone arginines	R-MMU-3214858	1.19	0
Synthesis of PIPs at the Golgi membrane	R-MMU-1660514	1.196	0.04456
Neurofascin interactions	R-MMU-447043	1.199	0.05801
Cargo concentration in the ER	R-MMU-5694530	1.2	0.08775
Formation of Incision Complex in GG-NER	R-MMU-5696395	1.203	0
Glyoxylate metabolism and glycine degradation	R-MMU-389661	1.203	0.02847
Reduction of cytosolic Ca++ levels	R-MMU-418359	1.207	0.02747
Peroxisomal protein import	R-MMU-9033241	1.214	0
Unblocking of NMDA receptors, glutamate binding anc	R-MMU-438066	1.214	0.0671
G beta:gamma signalling through CDC42	R-MMU-8964616	1.216	0.02631
MET activates STAT3	R-MMU-8875791	1.218	0.02747
Activation of BAD and translocation to mitochondria	R-MMU-111447	1.223	0.00453

SUMOylation of DNA damage response and repair prot	R-MMU-3108214	1.224	0.05801
Regulation of actin dynamics for phagocytic cup forma	R-MMU-2029482	1.225	0.00453
Oxidative Stress Induced Senescence	R-MMU-2559580	1.232	0
TP53 Regulates Metabolic Genes	R-MMU-5628897	1.237	0.00453
Ub-specific processing proteases	R-MMU-5689880	1.237	0.00453
Autodegradation of Cdh1 by Cdh1:APC/C	R-MMU-174084	1.237	0.00453
Synthesis of very long-chain fatty acyl-CoAs	R-MMU-75876	1.24	0.02747
SUMOylation of chromatin organization proteins	R-MMU-4551638	1.245	0.00453
RHO GTPases activate PKNs	R-MMU-5625740	1.247	0.00453
Acyl chain remodelling of PI	R-MMU-1482922	1.247	0.01755
PKA activation	R-MMU-163615	1.251	0.04169
Protein methylation	R-MMU-8876725	1.253	0.02847
Chk1/Chk2(Cds1) mediated inactivation of Cyclin B:Cdl	R-MMU-75035	1.256	0.00453
Regulation of localization of FOXO transcription factors	R-MMU-9614399	1.259	0.00453
Platelet degranulation	R-MMU-114608	1.259	0.02016
Deadenylation of mRNA	R-MMU-429947	1.26	0.00453
Regulation of cytoskeletal remodeling and cell spreadi	R-MMU-446388	1.264	0.06512
Pentose phosphate pathway	R-MMU-71336	1.264	0.08018
CDT1 association with the CDC6:ORC:origin complex	R-MMU-68827	1.266	0.00453
ISG15 antiviral mechanism	R-MMU-1169408	1.266	0.01755
Senescence-Associated Secretory Phenotype (SASP)	R-MMU-2559582	1.268	0.00453
DCC mediated attractive signaling	R-MMU-418885	1.271	0.00453
Pyrimidine salvage	R-MMU-73614	1.271	0.01755
Ca2+ pathway	R-MMU-4086398	1.274	0.04169
RUNX1 regulates genes involved in megakaryocyte diff	R-MMU-8936459	1.276	0.00453
Attenuation phase	R-MMU-3371568	1.276	0.04169
DNA Damage/Telomere Stress Induced Senescence	R-MMU-2559586	1.281	0
Acyl chain remodelling of PS	R-MMU-1482801	1.282	0.05502
Glutathione conjugation	R-MMU-156590	1.285	0.02318
Estrogen-dependent gene expression	R-MMU-9018519	1.286	0
Apoptotic execution phase	R-MMU-75153	1.287	0.00849
Factors involved in megakaryocyte development and p	R-MMU-983231	1.293	0
mRNA 3'-end processing	R-MMU-72187	1.298	0.02747
ADP signalling through P2Y purinoceptor 1	R-MMU-418592	1.298	0.08775
Long-term potentiation	R-MMU-9620244	1.299	0.07316
G alpha (i) signalling events	R-MMU-418594	1.305	0.06412
FGFR2 alternative splicing	R-MMU-6803529	1.306	0.00849
Cleavage of Growing Transcript in the Termination Reg	R-MMU-109688	1.306	0.0427
SUMOylation of ubiquitinylation proteins	R-MMU-3232142	1.315	0.02747
Tetrahydrobiopterin (BH4) synthesis, recycling, salvage	R-MMU-1474151	1.319	0.08775
Detoxification of Reactive Oxygen Species	R-MMU-3299685	1.321	0.01494
VEGFR2 mediated vascular permeability	R-MMU-5218920	1.322	0.032
Cooperation of PDCL (PhLP1) and TRiC/CCT in G-protei	R-MMU-6814122	1.323	0.00453
SUMO is transferred from E1 to E2 (UBE2I, UBC9)	R-MMU-3065678	1.325	0.00453
Methylation	R-MMU-156581	1.326	0.00453
eNOS activation	R-MMU-203615	1.327	0.0431
Dissolution of Fibrin Clot	R-MMU-75205	1.339	0.01158
Interconversion of nucleotide di- and triphosphates	R-MMU-499943	1.339	0.02847
Beta-oxidation of very long chain fatty acids	R-MMU-390247	1.339	0.03094
TNFR1-mediated ceramide production	R-MMU-5626978	1.342	0.00849
Translocation of SLC2A4 (GLUT4) to the plasma membr	R-MMU-1445148	1.352	0
Arachidonic acid metabolism	R-MMU-2142753	1.361	0.0134
SUMOylation of transcription factors	R-MMU-3232118	1.363	0.00453
B-WICH complex positively regulates rRNA expression	R-MMU-5250924	1.369	0.00453
Sema3A PAK dependent Axon repulsion	R-MMU-399954	1.377	0.01755
PI and PC transport between ER and Golgi membranes	R-MMU-1483196	1.377	0.02747
Oxygen-dependent asparagine hydroxylation of Hypox	R-MMU-1234162	1.383	0.02318
Biosynthesis of the N-glycan precursor (dolichol lipid-li	R-MMU-446193	1.39	0.04169
SUMOylation of DNA replication proteins	R-MMU-4615885	1.393	0.01158
The NLRP3 inflammasome	R-MMU-844456	1.393	0.02847

Condensation of Prophase Chromosomes	R-MMU-2299718	1.394	0
Signaling by Retinoic Acid	R-MMU-5362517	1.394	0.00453
FCERI mediated Ca+2 mobilization	R-MMU-2871809	1.396	0.02631
Import of palmitoyl-CoA into the mitochondrial matrix	R-MMU-200425	1.398	0
Purine catabolism	R-MMU-74259	1.4	0.01755
HSF1 activation	R-MMU-3371511	1.4	0.02847
Mitochondrial protein import	R-MMU-1268020	1.403	0
Glycogen breakdown (glycogenolysis)	R-MMU-70221	1.403	0.03269
Citric acid cycle (TCA cycle)	R-MMU-71403	1.405	0.00453
Hyaluronan uptake and degradation	R-MMU-2160916	1.416	0.02747
Regulation of pyruvate dehydrogenase (PDH) complex	R-MMU-204174	1.416	0.09874
RNA Polymerase I Chain Elongation	R-MMU-73777	1.417	0
Amplification of signal from unattached kinetochores	R-MMU-141444	1.417	0.00453
SUMO is proteolytically processed	R-MMU-3065679	1.422	0.01158
Transcriptional regulation by small RNAs	R-MMU-5578749	1.426	0.00453
MHC class II antigen presentation	R-MMU-2132295	1.43	0
Synthesis of IP3 and IP4 in the cytosol	R-MMU-1855204	1.431	0.03094
SUMOylation of SUMOylation proteins	R-MMU-4085377	1.432	0.00453
COPI-mediated anterograde transport	R-MMU-6807878	1.442	0
phospho-PLA2 pathway	R-MMU-111995	1.444	0.03467
snRNP Assembly	R-MMU-191859	1.446	0
APC/C:Cdc20 mediated degradation of mitotic protein	R-MMU-176409	1.446	0.02631
Inactivation of APC/C via direct inhibition of the APC/C	R-MMU-141430	1.446	0.02747
Activated PKN1 stimulates transcription of AR (androg	R-MMU-5625886	1.452	0.00453
Hydrolysis of LPC	R-MMU-1483115	1.458	0.01755
Regulation of PLK1 Activity at G2/M Transition	R-MMU-2565942	1.461	0
Protein repair	R-MMU-5676934	1.466	0.08775
Separation of Sister Chromatids	R-MMU-2467813	1.47	0
RNA Polymerase I Promoter Opening	R-MMU-73728	1.471	0
Major pathway of rRNA processing in the nucleolus an	R-MMU-6791226	1.474	0
NoRC negatively regulates rRNA expression	R-MMU-427413	1.475	0
RHO GTPases Activate Formins	R-MMU-5663220	1.476	0
SIRT1 negatively regulates rRNA expression	R-MMU-427359	1.477	0
PRC2 methylates histones and DNA	R-MMU-212300	1.481	0.00453
ESR-mediated signaling	R-MMU-8939211	1.484	0.02747
Ethanol oxidation	R-MMU-71384	1.485	0.09874
Alpha-oxidation of phytanate	R-MMU-389599	1.487	0.00453
Beta-oxidation of pristanoyl-CoA	R-MMU-389887	1.488	0.00453
PTK6 promotes HIF1A stabilization	R-MMU-8857538	1.495	0.01158
Synthesis of IP2, IP, and Ins in the cytosol	R-MMU-1855183	1.501	0.01755
Anchoring of the basal body to the plasma membrane	R-MMU-5620912	1.505	0
G alpha (q) signalling events	R-MMU-416476	1.506	0.04791
Amino acid synthesis and interconversion (transamina	R-MMU-70614	1.524	0.04456
WNT ligand biogenesis and trafficking	R-MMU-3238698	1.531	0
Association of TriC/CCT with target proteins during bio	R-MMU-390471	1.532	0
Selenocysteine synthesis	R-MMU-2408557	1.533	0.00453
Purine ribonucleoside monophosphate biosynthesis	R-MMU-73817	1.534	0.01158
Recruitment of mitotic centrosome proteins and comp	R-MMU-380270	1.535	0
Ribosomal scanning and start codon recognition	R-MMU-72702	1.541	0
L13a-mediated translational silencing of Ceruloplasmin	R-MMU-156827	1.543	0
Apoptotic cleavage of cellular proteins	R-MMU-111465	1.544	0.04048
COPI-dependent Golgi-to-ER retrograde traffic	R-MMU-6811434	1.546	0
Translation initiation complex formation	R-MMU-72649	1.547	0
Recycling pathway of L1	R-MMU-437239	1.55	0.00673
Abacavir metabolism	R-MMU-2161541	1.562	0.09874
RA biosynthesis pathway	R-MMU-5365859	1.568	0.09874
RHO GTPases activate IQGAPs	R-MMU-5626467	1.572	0
Formation of the ternary complex, and subsequently, t	R-MMU-72695	1.588	0
Formation of a pool of free 40S subunits	R-MMU-72689	1.59	0
Biotin transport and metabolism	R-MMU-196780	1.591	0.03097

Synthesis of Prostaglandins (PG) and Thromboxanes (T	R-MMU-2162123	1.595	0.05206
Loss of proteins required for interphase microtubule o	R-MMU-380284	1.603	0
Loss of Nlp from mitotic centrosomes	R-MMU-380259	1.603	0
Activation of DNA fragmentation factor	R-MMU-211227	1.616	0.00453
Hypusine synthesis from eIF5A-lysine	R-MMU-204626	1.621	0
Negative regulation of activity of TFAP2 (AP-2) family t	R-MMU-8866904	1.63	0.00453
NADE modulates death signalling	R-MMU-205025	1.631	0.01158
Displacement of DNA glycosylase by APEX1	R-MMU-110357	1.634	0.00453
Peptide ligand-binding receptors	R-MMU-375276	1.634	0.02016
Recruitment of NuMA to mitotic centrosomes	R-MMU-380320	1.635	0
Resolution of Sister Chromatid Cohesion	R-MMU-2500257	1.639	0
COPI-independent Golgi-to-ER retrograde traffic	R-MMU-6811436	1.641	0
AURKA Activation by TPX2	R-MMU-8854518	1.643	0
Mitotic Prometaphase	R-MMU-68877	1.647	0
Generation of second messenger molecules	R-MMU-202433	1.654	0.06512
Branched-chain amino acid catabolism	R-MMU-70895	1.662	0.00453
The role of GTSE1 in G2/M progression after G2 checkp	R-MMU-8852276	1.665	0
RHO GTPases Activate Rhotekin and Rhophilins	R-MMU-5666185	1.672	0.00453
CDC6 association with the ORC:origin complex	R-MMU-68689	1.672	0.04791
SUMOylation of DNA methylation proteins	R-MMU-4655427	1.693	0
Metabolism of Angiotensinogen to Angiotensins	R-MMU-2022377	1.697	0.02747
HSP90 chaperone cycle for steroid hormone receptors	R-MMU-3371497	1.709	0
Synthesis of 15-eicosatetraenoic acid derivatives	R-MMU-2142770	1.733	0.01158
Vitamin D (calciferol) metabolism	R-MMU-196791	1.736	0
Interleukin-4 and Interleukin-13 signaling	R-MMU-6785807	1.741	0.01158
CLEC7A/inflammasome pathway	R-MMU-5660668	1.746	0.00453
Intraflagellar transport	R-MMU-5620924	1.782	0
Hedgehog 'off' state	R-MMU-5610787	1.782	0.00453
Synthesis of 12-eicosatetraenoic acid derivatives	R-MMU-2142712	1.785	0.00453
Sulfur amino acid metabolism	R-MMU-1614635	1.812	0
Assembly of the pre-replicative complex	R-MMU-68867	1.826	0.01755
Kinesins	R-MMU-983189	1.862	0
Cleavage of the damaged pyrimidine	R-MMU-110329	1.864	0.00453
Cytosolic tRNA aminoacylation	R-MMU-379716	1.867	0
Resolution of D-loop Structures through Holliday Junct	R-MMU-5693568	1.881	0.02747
Pyrophosphate hydrolysis	R-MMU-71737	1.891	0.00849
Assembly of the ORC complex at the origin of replicatio	R-MMU-68616	1.903	0.02847
SUMOylation of RNA binding proteins	R-MMU-4570464	1.927	0
5-Phosphoribose 1-diphosphate biosynthesis	R-MMU-73843	1.933	0.05232
Cilium Assembly	R-MMU-5617833	1.94	0
Carboxyterminal post-translational modifications of tu	R-MMU-8955332	1.968	0
Switching of origins to a post-replicative state	R-MMU-69052	1.977	0.03097
Androgen biosynthesis	R-MMU-193048	1.998	0.01158
Activation of the mRNA upon binding of the cap-bindir	R-MMU-72662	2.004	0.00849
Microtubule-dependent trafficking of connexons from	R-MMU-190840	2.014	0
Inhibition of the proteolytic activity of APC/C required	R-MMU-141405	2.044	0.09874
Pre-NOTCH Processing in Golgi	R-MMU-1912420	2.06	0.02016
Advanced glycosylation endproduct receptor signaling	R-MMU-879415	2.061	0
Glucuronidation	R-MMU-156588	2.096	0.02016
Chemokine receptors bind chemokines	R-MMU-380108	2.177	0.02016
Acyl chain remodeling of CL	R-MMU-1482798	2.205	0
NADPH regeneration	R-MMU-389542	2.252	0
Beta oxidation of lauroyl-CoA to decanoyl-CoA-CoA	R-MMU-77310	2.253	0
Wax biosynthesis	R-MMU-8848584	2.317	0.01494
Synthesis of Ketone Bodies	R-MMU-77111	2.33	0
Telomere Extension By Telomerase	R-MMU-171319	2.503	0.08018
Mitotic Telophase/Cytokinesis	R-MMU-68884	2.551	0
Glycine degradation	R-MMU-6783984	2.588	0.00453
Triglyceride catabolism	R-MMU-163560	2.589	0.02631
Beta oxidation of myristoyl-CoA to lauroyl-CoA	R-MMU-77285	2.595	0

TET1,2,3 and TDG demethylate DNA	R-MMU-5221030	2.595	0.00453
Synthesis of (16-20)-hydroxyeicosatetraenoic acids (HE	R-MMU-2142816	2.973	0.07272
Sensing of DNA Double Strand Breaks	R-MMU-5693548	3.303	0.00453
Downstream signaling of activated FGFR1	R-MMU-5654687	4.22	0.04527

Supplementary data 2: List of differently regulated pathways in small intestinal tuft cells under HFD-feeding vs RFD-feeding conditions for 22 weeks.

PathwayName	PathwayID	HFD/RFD (fold change)_22 wks	*q-value_* 22 wks
Antimicrobial peptides	R-MMU-6803157	0.135	0.04661
Metal sequestration by anti	R-MMU-6799990	0.39	0.03722
DAP12 interactions	R-MMU-2172127	0.4	0
Signaling by Insulin receptor	R-MMU-74752	0.446	0.05953
GABA B receptor activation	R-MMU-977444	0.451	0
Endosomal/Vacuolar pathw	R-MMU-1236977	0.506	0
Proline catabolism	R-MMU-70688	0.532	0
ER-Phagosome pathway	R-MMU-1236974	0.532	0
DEx/H-box helicases activat	R-MMU-3134963	0.554	0
Antigen Presentation: Foldir	R-MMU-983170	0.557	0
TICAM1-dependent activati	R-MMU-9013973	0.566	0
Immunoregulatory interacti	R-MMU-198933	0.574	0
IRS activation	R-MMU-74713	0.593	0.05953
TRAF6 mediated IRF7 activa	R-MMU-933541	0.607	0
TRAF3-dependent IRF activa	R-MMU-918233	0.607	0
Glycerophospholipid catabo	R-MMU-6814848	0.61	0
DSCAM interactions	R-MMU-376172	0.652	0
Acetylcholine Neurotransmi	R-MMU-264642	0.708	0.04661
DAP12 signaling	R-MMU-2424491	0.722	0.01238
Retinoid metabolism and tra	R-MMU-975634	0.757	0.04974
Tight junction interactions	R-MMU-420029	0.768	0.05953
Activation of C3 and C5	R-MMU-174577	0.781	0.01238
Activation of IRF3/IRF7 med	R-MMU-936964	0.796	0
Synthesis of PI	R-MMU-1483226	0.796	0.02444
Alternative complement act	R-MMU-173736	0.803	0.04661
Reactions specific to the cor	R-MMU-975578	0.805	0
TRAF6 mediated NF-kB activ	R-MMU-933542	0.808	0.09714
TNF receptor superfamily (T	R-MMU-5676594	0.815	0.03373
TRAF6 mediated IRF7 activa	R-MMU-975110	0.821	0
Basigin interactions	R-MMU-210991	0.834	0.02444
PKMTs methylate histone ly	R-MMU-3214841	0.841	0.05029
Interleukin-20 family signali	R-MMU-8854691	0.852	0.05953
HDMs demethylate histones	R-MMU-3214842	0.865	0.01238
HDACs deacetylate histones	R-MMU-3214815	0.867	0.01238
Negative regulators of DDX5	R-MMU-936440	0.883	0.04974
Transcriptional activation of	R-MMU-2151201	0.885	0.07871
Macroautophagy	R-MMU-1632852	0.906	0.04661
Regulation of TNFR1 signalir	R-MMU-5357905	0.911	0.04661
SUMOylation of transcriptio	R-MMU-3899300	0.949	0.04974
Clathrin-mediated endocyto	R-MMU-8856828	1.034	0.06276
Pink/Parkin Mediated Mitop	R-MMU-5205685	1.036	0.09861
mRNA Splicing - Major Path	R-MMU-72163	1.043	0.07785
CDT1 association with the C	R-MMU-68827	1.048	0.05858
Autodegradation of Cdh1 by	R-MMU-174084	1.05	0.09738
Regulation of actin dynamic	R-MMU-2029482	1.051	0.08254
UCH proteinases	R-MMU-5689603	1.063	0.00655
Neddylation	R-MMU-8951664	1.067	0.01173
Formation of TC-NER Pre-Inc	R-MMU-6781823	1.067	0.01594
DNA Damage Recognition in	R-MMU-5696394	1.08	0.01173
Synthesis of UDP-N-acetyl-g	R-MMU-446210	1.094	0.04754
Synthesis of PIPs at the Golg	R-MMU-1660514	1.097	0.0248
B-WICH complex positively r	R-MMU-5250924	1.099	0.0569
MAP2K and MAPK activation	R-MMU-5674135	1.104	0.0206
Peroxisomal protein import	R-MMU-9033241	1.107	0
Cooperation of PDCL (PhLP1	R-MMU-6814122	1.111	0
Golgi Cisternae Pericentriola	R-MMU-162658	1.117	0
Transport of Mature mRNA	R-MMU-159236	1.117	0.05381
Lysine catabolism	R-MMU-71064	1.118	0.07785

Synthesis of very long-chain	R-MMU-75876	1.119	0
RHO GTPases Activate WASI	R-MMU-5663213	1.123	0.01173
Mitochondrial translation el	R-MMU-5389840	1.124	0.05381
Serine biosynthesis	R-MMU-977347	1.125	0.03748
Mitochondrial translation te	R-MMU-5419276	1.125	0.07785
Gap junction degradation	R-MMU-190873	1.136	0.01594
RHO GTPases activate CIT	R-MMU-5625900	1.139	0.04331
SUMOylation of DNA damag	R-MMU-3108214	1.145	0.08254
Synthesis, secretion, and de	R-MMU-422085	1.145	0.09738
Formation of annular gap ju	R-MMU-196025	1.148	0.01173
FGFR2 alternative splicing	R-MMU-6803529	1.15	0.00655
Vitamin B5 (pantothenate) r	R-MMU-199220	1.158	0.04331
Adherens junctions interacti	R-MMU-418990	1.159	0.00655
Hyaluronan uptake and deg	R-MMU-2160916	1.164	0.0506
RHO GTPases activate IQGA	R-MMU-5626467	1.165	0.0206
Interaction between L1 and	R-MMU-445095	1.176	0.00655
TP53 Regulates Metabolic G	R-MMU-5628897	1.179	0
Cell-extracellular matrix inte	R-MMU-446353	1.179	0
Deadenylation of mRNA	R-MMU-429947	1.194	0
TNFR1-mediated ceramide p	R-MMU-5626978	1.194	0.03748
Detoxification of Reactive O	R-MMU-3299685	1.198	0.01383
SUMO is conjugated to E1 (U	R-MMU-3065676	1.199	0.07785
SUMOylation of DNA replica	R-MMU-4615885	1.202	0.01383
Interconversion of nucleotic	R-MMU-499943	1.203	0.00655
Metabolism of Angiotensinc	R-MMU-2022377	1.207	0.01594
Trafficking and processing o	R-MMU-1679131	1.21	0
Pentose phosphate pathway	R-MMU-71336	1.217	0.00655
RHO GTPases activate PKNs	R-MMU-5625740	1.221	0
Alpha-oxidation of phytanat	R-MMU-389599	1.226	0.03589
Interleukin-4 and Interleukir	R-MMU-6785807	1.234	0.00655
Regulation of gene expressio	R-MMU-1234158	1.234	0.0206
Glyoxylate metabolism and	R-MMU-389661	1.235	0.08254
Activation of BAD and transl	R-MMU-111447	1.237	0
Import of palmitoyl-CoA into	R-MMU-200425	1.237	0
Nuclear Pore Complex (NPC	R-MMU-3301854	1.238	0.0248
KSRP (KHSRP) binds and des	R-MMU-450604	1.239	0
Methylation	R-MMU-156581	1.239	0.0569
RHO GTPases Activate Form	R-MMU-5663220	1.242	0
RHO GTPases Activate Rhote	R-MMU-5666185	1.242	0.07785
Regulation of localization of	R-MMU-9614399	1.243	0
Rap1 signalling	R-MMU-392517	1.243	0
Signaling by Hippo	R-MMU-2028269	1.245	0.01594
Translocation of SLC2A4 (GL	R-MMU-1445148	1.247	0
Recycling pathway of L1	R-MMU-437239	1.249	0
Heme biosynthesis	R-MMU-189451	1.254	0.00655
snRNP Assembly	R-MMU-191859	1.256	0.0248
Regulation of PLK1 Activity a	R-MMU-2565942	1.258	0.00655
Major pathway of rRNA proc	R-MMU-6791226	1.259	0
GP1b-IX-V activation signalli	R-MMU-430116	1.26	0
Chk1/Chk2(Cds1) mediated	R-MMU-75035	1.263	0
SUMO is transferred from E1	R-MMU-3065678	1.265	0.09738
Biosynthesis of the N-glycan	R-MMU-446193	1.268	0.0248
L13a-mediated translational	R-MMU-156827	1.278	0
Formation of the ternary co	R-MMU-72695	1.278	0
MHC class II antigen present	R-MMU-2132295	1.278	0
Branched-chain amino acid	R-MMU-70895	1.279	0.07785
Formation of a pool of free 4	R-MMU-72689	1.28	0
Signaling by Retinoic Acid	R-MMU-5362517	1.282	0.01173
Ribosomal scanning and sta	R-MMU-72702	1.286	0
DCC mediated attractive sig	R-MMU-418885	1.286	0.00655
NOTCH4 Activation and Tran	R-MMU-9013700	1.29	0
Anchoring of the basal body	R-MMU-5620912	1.29	0.00655

Androgen biosynthesis	R-MMU-193048	1.29	0.01383
Association of TriC/CCT with	R-MMU-390471	1.291	0.00655
The fatty acid cycling model	R-MMU-167826	1.291	0.0248
The proton buffering model	R-MMU-167827	1.291	0.03748
Translation initiation comple	R-MMU-72649	1.292	0
Separation of Sister Chroma	R-MMU-2467813	1.293	0
Selenocysteine synthesis	R-MMU-2408557	1.307	0.0569
Recruitment of mitotic centr	R-MMU-380270	1.308	0.00655
COPI-mediated anterograde	R-MMU-6807878	1.31	0.01173
Scavenging by Class A Recep	R-MMU-3000480	1.316	0
Loss of proteins required for	R-MMU-380284	1.321	0.00655
Loss of Nlp from mitotic cen	R-MMU-380259	1.321	0.00655
O-linked glycosylation	R-MMU-5173105	1.321	0.07367
AURKA Activation by TPX2	R-MMU-8854518	1.327	0.00655
Peptide ligand-binding recep	R-MMU-375276	1.327	0.00655
ESR-mediated signaling	R-MMU-8939211	1.335	0.08254
NADE modulates death sign	R-MMU-205025	1.336	0.01173
Synthesis of 15-eicosatetrae	R-MMU-2142770	1.337	0
Recruitment of NuMA to mi	R-MMU-380320	1.338	0
Arachidonic acid metabolism	R-MMU-2142753	1.346	0.07785
Miscellaneous substrates	R-MMU-211958	1.351	0.03928
alpha-linolenic acid (ALA) m	R-MMU-2046106	1.354	0.08746
Mitotic Prometaphase	R-MMU-68877	1.358	0
Resolution of Sister Chroma	R-MMU-2500257	1.361	0
COPI-dependent Golgi-to-ER	R-MMU-6811434	1.362	0
Hypusine synthesis from eIF	R-MMU-204626	1.367	0
Oxygen-dependent asparagi	R-MMU-1234162	1.37	0.00655
COPI-independent Golgi-to-I	R-MMU-6811436	1.374	0.00655
Purine catabolism	R-MMU-74259	1.39	0
Degradation of cysteine and	R-MMU-1614558	1.402	0.00655
Synthesis of 12-eicosatetrae	R-MMU-2142712	1.417	0
Hedgehog 'off' state	R-MMU-5610787	1.418	0.00655
PTK6 promotes HIF1A stabili	R-MMU-8857538	1.419	0
Cobalamin (Cbl, vitamin B12	R-MMU-196741	1.421	0
Eicosanoids	R-MMU-211979	1.422	0.08254
Intraflagellar transport	R-MMU-5620924	1.424	0.01383
HSP90 chaperone cycle for s	R-MMU-3371497	1.426	0
The role of GTSE1 in G2/M p	R-MMU-8852276	1.439	0
SUMOylation of RNA binding	R-MMU-4570464	1.442	0
Beta oxidation of lauroyl-Co	R-MMU-77310	1.449	0
Acyl chain remodeling of CL	R-MMU-1482798	1.462	0
Hydrolysis of LPC	R-MMU-1483115	1.47	0.0506
NADPH regeneration	R-MMU-389542	1.478	0.01173
Propionyl-CoA catabolism	R-MMU-71032	1.491	0.00655
Kinesins	R-MMU-983189	1.492	0.00655
Arachidonate production fro	R-MMU-426048	1.496	0.09738
5-Phosphoribose 1-diphosph	R-MMU-73843	1.517	0.00655
Cilium Assembly	R-MMU-5617833	1.532	0.00655
Purine ribonucleoside monc	R-MMU-73817	1.541	0
Heme degradation	R-MMU-189483	1.545	0.00655
Carboxyterminal post-transl	R-MMU-8955332	1.545	0.00655
Beta oxidation of myristoyl-(	R-MMU-77285	1.546	0
Microtubule-dependent trai	R-MMU-190840	1.569	0.00655
Lipid particle organization	R-MMU-8964572	1.585	0.07215
Advanced glycosylation end	R-MMU-879415	1.604	0.03398
CLEC7A/inflammasome path	R-MMU-5660668	1.607	0
Synthesis of Ketone Bodies	R-MMU-77111	1.615	0
Amino acid synthesis and in	R-MMU-70614	1.642	0.08254
Mitochondrial transcription	R-MMU-163282	1.65	0.00655
CDC6 association with the O	R-MMU-68689	1.651	0.01383
Agmatine biosynthesis	R-MMU-351143	1.832	0
Sensing of DNA Double Strai	R-MMU-5693548	1.856	0.01383

Assembly of the ORC comple	R-MMU-68616		1.881	0.01173
Glucuronidation	R-MMU-156588		1.901	0.00655
Activation of the mRNA upo	R-MMU-72662		1.908	0
Glycine degradation	R-MMU-6783984		1.961	0.09861
Assembly of the pre-replicat	R-MMU-68867		2.013	0.01594
Pyrophosphate hydrolysis	R-MMU-71737		2.069	0
Scavenging by Class B Recep	R-MMU-3000471		2.136	0.00655
Wax biosynthesis	R-MMU-8848584		2.199	0.0206
Abacavir transmembrane tra	R-MMU-2161517		2.248	0.00655
Cytosolic tRNA aminoacylati	R-MMU-379716		2.285	0
TET1,2,3 and TDG demethyla	R-MMU-5221030		2.305	0.05006
Sulfur amino acid metabolis	R-MMU-1614635		2.467	0
Telomere Extension By Telo	R-MMU-171319		2.97	0
Synthesis of (16-20)-hydroxy	R-MMU-2142816		5.424	0

Supplementary data 2: List of differently regulated pathways in small intestinal tuft cells under HFD-feeding vs RFD-feeding conditions for 9 and 22 weeks.

PathwayName	PathwayID	HFD/RFD (fold change)_9 wks	*q-value_* 9wk	shared	HFD/RFD (fold change)_22 wks	q-value_22	shared
Metal sequestratio	R-MMU-67999	0	0.02949	shared	0.39	0.03722	shared
Signaling by Insulin	R-MMU-74752	0.075	0.03876	shared	0.446	0.05953	shared
IRS activation	R-MMU-74713	0.188	0.03531	shared	0.593	0.05953	shared
Antimicrobial pept	R-MMU-68031	0.345	0.02949	shared	0.135	0.04661	shared
DAP12 interactions	R-MMU-21721	0.405	0.04833	shared	0.4	0	shared
Endosomal/Vacuol	R-MMU-12369	0.413	0.02949	shared	0.506	0	shared
ER-Phagosome pat	R-MMU-12369	0.446	0.02949	shared	0.532	0	shared
Acetylcholine Neur	R-MMU-26464	0.516	0.03113	shared	0.708	0.04661	shared
Glycerophospholip	R-MMU-68148	0.533	0.04833	shared	0.61	0	shared
Antigen Presentati	R-MMU-98317	0.535	0.02949	shared	0.557	0	shared
Synthesis of PI	R-MMU-14832	0.597	0.03113	shared	0.796	0.02444	shared
DEx/H-box helicase	R-MMU-31349	0.6	0.03876	shared	0.554	0	shared
DSCAM interaction	R-MMU-37617	0.619	0.03876	shared	0.652	0	shared
TICAM1-dependen	R-MMU-90139	0.621	0.04833	shared	0.566	0	shared
TRAF3-dependent	R-MMU-91823	0.634	0.04809	shared	0.607	0	shared
TRAF6 mediated IR	R-MMU-93354	0.634	0.03592	shared	0.607	0	shared
Tight junction inte	R-MMU-42002	0.647	0.02949	shared	0.768	0.05953	shared
Reactions specific t	R-MMU-97557	0.667	0.03531	shared	0.805	0	shared
Proline catabolism	R-MMU-70688	0.676	0.03876	shared	0.532	0	shared
Interleukin-20 fam	R-MMU-88546	0.731	0.02949	shared	0.852	0.05953	shared
Basigin interaction	R-MMU-21099	0.754	0.04809	shared	0.834	0.02444	shared
TRAF6 mediated N	R-MMU-93354	0.771	0.04809	shared	0.808	0.09714	shared
Negative regulator	R-MMU-93644	0.787	0.06592	shared	0.883	0.04974	shared
Regulation of TNFR	R-MMU-53579	0.822	0.05249	shared	0.911	0.04661	shared
PKMTs methylate h	R-MMU-32148	0.84	0.08557	shared	0.841	0.05029	shared
Activation of IRF3/	R-MMU-93696	0.853	0.071	shared	0.796	0	shared
Macroautophagy	R-MMU-16328	0.908	0.03876	shared	0.906	0.04661	shared
Neddylation	R-MMU-89516	1.088	0.02847	shared	1.067	0.01173	shared
RHO GTPases Activ	R-MMU-56632	1.119	0.04791	shared	1.123	0.01173	shared
UCH proteinases	R-MMU-56896	1.147	0.00453	shared	1.063	0.00655	shared
Golgi Cisternae Per	R-MMU-16265	1.152	0.04169	shared	1.117	0	shared
Formation of TC-NI	R-MMU-67818	1.16	0.00453	shared	1.067	0.01594	shared
mRNA Splicing - Ma	R-MMU-72163	1.187	0.01158	shared	1.043	0.07785	shared
Synthesis of PIPs at	R-MMU-16605	1.196	0.04456	shared	1.097	0.0248	shared
Glyoxylate metabo	R-MMU-38966	1.203	0.02847	shared	1.235	0.08254	shared
Peroxisomal protei	R-MMU-90332	1.214	0	shared	1.107	0	shared
Activation of BAD a	R-MMU-11144	1.223	0.00453	shared	1.237	0	shared
SUMOylation of DN	R-MMU-31082	1.224	0.05801	shared	1.145	0.08254	shared
Regulation of actin	R-MMU-20294	1.225	0.00453	shared	1.051	0.08254	shared
Autodegradation o	R-MMU-17408	1.237	0.00453	shared	1.05	0.09738	shared
TP53 Regulates Me	R-MMU-56288	1.237	0.00453	shared	1.179	0	shared
Synthesis of very lo	R-MMU-75876	1.24	0.02747	shared	1.119	0	shared
RHO GTPases activ	R-MMU-56257	1.247	0.00453	shared	1.221	0	shared
Chk1/Chk2(Cds1) m	R-MMU-75035	1.256	0.00453	shared	1.263	0	shared
Regulation of local	R-MMU-96143	1.259	0.00453	shared	1.243	0	shared
Deadenylation of m	R-MMU-42994	1.26	0.00453	shared	1.194	0	shared
Pentose phosphate	R-MMU-71336	1.264	0.08018	shared	1.217	0.00655	shared
CDT1 association w	R-MMU-68827	1.266	0.00453	shared	1.048	0.05858	shared
DCC mediated attr	R-MMU-41888	1.271	0.00453	shared	1.286	0.00655	shared
FGFR2 alternative s	R-MMU-68035	1.306	0.00849	shared	1.15	0.00655	shared
Detoxification of R	R-MMU-32996	1.321	0.01494	shared	1.198	0.01383	shared
Cooperation of PD	R-MMU-68141	1.323	0.00453	shared	1.111	0	shared
SUMO is transferre	R-MMU-30656	1.325	0.00453	shared	1.265	0.09738	shared
Methylation	R-MMU-15658	1.326	0.00453	shared	1.239	0.0569	shared
Interconversion of	R-MMU-49994	1.339	0.02847	shared	1.203	0.00655	shared
TNFR1-mediated ce	R-MMU-56269	1.342	0.00849	shared	1.194	0.03748	shared
Translocation of SL	R-MMU-14451	1.352	0	shared	1.247	0	shared
Arachidonic acid m	R-MMU-21427	1.361	0.0134	shared	1.346	0.07785	shared
B-WICH complex p	R-MMU-52509	1.369	0.00453	shared	1.099	0.0569	shared
Oxygen-dependent	R-MMU-12341	1.383	0.02318	shared	1.37	0.00655	shared
Biosynthesis of the	R-MMU-44619	1.39	0.04169	shared	1.268	0.0248	shared
SUMOylation of DN	R-MMU-46158	1.393	0.01158	shared	1.202	0.01383	shared
Signaling by Retino	R-MMU-53625	1.394	0.00453	shared	1.282	0.01173	shared
Import of palmitoy	R-MMU-20042	1.398	0	shared	1.237	0	shared
Purine catabolism	R-MMU-74259	1.4	0.01755	shared	1.39	0	shared
Hyaluronan uptake	R-MMU-21609	1.416	0.02747	shared	1.164	0.0506	shared
MHC class II antige	R-MMU-21322	1.43	0	shared	1.278	0	shared

COPI-mediated ant	R-MMU-68078	1.442	0	shared	1.31	0.01173	shared
snRNP Assembly	R-MMU-19185	1.446	0	shared	1.256	0.0248	shared
Hydrolysis of LPC	R-MMU-14831	1.458	0.01755	shared	1.47	0.0506	shared
Regulation of PLK1	R-MMU-25659	1.461	0	shared	1.258	0.00655	shared
Separation of Siste	R-MMU-24678	1.47	0	shared	1.293	0	shared
Major pathway of r	R-MMU-67912	1.474	0	shared	1.259	0	shared
RHO GTPases Activ	R-MMU-56632	1.476	0	shared	1.242	0	shared
ESR-mediated signa	R-MMU-89392	1.484	0.02747	shared	1.335	0.08254	shared
Alpha-oxidation of	R-MMU-38959	1.487	0.00453	shared	1.226	0.03589	shared
PTK6 promotes HIF	R-MMU-88575	1.495	0.01158	shared	1.419	0	shared
Anchoring of the b	R-MMU-56209	1.505	0	shared	1.29	0.00655	shared
Amino acid synthes	R-MMU-70614	1.524	0.04456	shared	1.642	0.08254	shared
Association of TriC	R-MMU-39047	1.532	0	shared	1.291	0.00655	shared
Selenocysteine syn	R-MMU-24085	1.533	0.00453	shared	1.307	0.0569	shared
Purine ribonucleos	R-MMU-73817	1.534	0.01158	shared	1.541	0	shared
Recruitment of mit	R-MMU-38027	1.535	0	shared	1.308	0.00655	shared
Ribosomal scannin	R-MMU-72702	1.541	0	shared	1.286	0	shared
L13a-mediated tra	R-MMU-15682	1.543	0	shared	1.278	0	shared
COPI-dependent G	R-MMU-68114	1.546	0	shared	1.362	0	shared
Translation initiatio	R-MMU-72649	1.547	0	shared	1.292	0	shared
Recycling pathway	R-MMU-43723	1.55	0.00673	shared	1.249	0	shared
RHO GTPases activ	R-MMU-56264	1.572	0	shared	1.165	0.0206	shared
Formation of the te	R-MMU-72695	1.588	0	shared	1.278	0	shared
Formation of a poo	R-MMU-72689	1.59	0	shared	1.28	0	shared
Loss of Nlp from m	R-MMU-38025	1.603	0	shared	1.321	0.00655	shared
Loss of proteins re	R-MMU-38028	1.603	0	shared	1.321	0.00655	shared
Hypusine synthesis	R-MMU-20462	1.621	0	shared	1.367	0	shared
NADE modulates d	R-MMU-20502	1.631	0.01158	shared	1.336	0.01173	shared
Peptide ligand-bind	R-MMU-37527	1.634	0.02016	shared	1.327	0.00655	shared
Recruitment of Nu	R-MMU-38032	1.635	0	shared	1.338	0	shared
Resolution of Sister	R-MMU-25002	1.639	0	shared	1.361	0	shared
COPI-independent	R-MMU-68114	1.641	0	shared	1.374	0.00655	shared
AURKA Activation b	R-MMU-88545	1.643	0	shared	1.327	0.00655	shared
Mitotic Prometaph	R-MMU-68877	1.647	0	shared	1.358	0	shared
Branched-chain am	R-MMU-70895	1.662	0.00453	shared	1.279	0.07785	shared
The role of GTSE1 i	R-MMU-88522	1.665	0	shared	1.439	0	shared
RHO GTPases Activ	R-MMU-56661	1.672	0.00453	shared	1.242	0.07785	shared
CDC6 association w	R-MMU-68689	1.672	0.04791	shared	1.651	0.01383	shared
Metabolism of Ang	R-MMU-20223	1.697	0.02747	shared	1.207	0.01594	shared
HSP90 chaperone c	R-MMU-33714	1.709	0	shared	1.426	0	shared
Synthesis of 15-eic	R-MMU-21427	1.733	0.01158	shared	1.337	0	shared
Interleukin-4 and I	R-MMU-67858	1.741	0.01158	shared	1.234	0.00655	shared
CLEC7A/inflammas	R-MMU-56606	1.746	0.00453	shared	1.607	0	shared
Hedgehog 'off' stat	R-MMU-56107	1.782	0.00453	shared	1.418	0.00655	shared
Intraflagellar trans	R-MMU-56209	1.782	0	shared	1.424	0.01383	shared
Synthesis of 12-eic	R-MMU-21427	1.785	0.00453	shared	1.417	0	shared
Sulfur amino acid r	R-MMU-16146	1.812	0	shared	2.467	0	shared
Assembly of the pr	R-MMU-68867	1.826	0.01755	shared	2.013	0.01594	shared
Kinesins	R-MMU-98318	1.862	0	shared	1.492	0.00655	shared
Cytosolic tRNA ami	R-MMU-37971	1.867	0	shared	2.285	0	shared
Pyrophosphate hyd	R-MMU-71737	1.891	0.00849	shared	2.069	0	shared
Assembly of the OF	R-MMU-68616	1.903	0.02847	shared	1.881	0.01173	shared
SUMOylation of RN	R-MMU-45704	1.927	0	shared	1.442	0	shared
5-Phosphoribose 1	R-MMU-73843	1.933	0.05232	shared	1.517	0.00655	shared
Cilium Assembly	R-MMU-56178	1.94	0	shared	1.532	0.00655	shared
Carboxyterminal p	R-MMU-89553	1.968	0	shared	1.545	0.00655	shared
Androgen biosynth	R-MMU-19304	1.998	0.01158	shared	1.29	0.01383	shared
Activation of the m	R-MMU-72662	2.004	0.00849	shared	1.908	0	shared
Microtubule-deper	R-MMU-19084	2.014	0	shared	1.569	0.00655	shared
Advanced glycosyla	R-MMU-87941	2.061	0	shared	1.604	0.03398	shared
Glucuronidation	R-MMU-15658	2.096	0.02016	shared	1.901	0.00655	shared
Acyl chain remodel	R-MMU-14827	2.205	0	shared	1.462	0	shared
NADPH regeneratio	R-MMU-38954	2.252	0	shared	1.478	0.01173	shared
Beta oxidation of la	R-MMU-77310	2.253	0	shared	1.449	0	shared
Wax biosynthesis	R-MMU-88485	2.317	0.01494	shared	2.199	0.0206	shared
Synthesis of Keton	R-MMU-77111	2.33	0	shared	1.615	0	shared
Telomere Extensio	R-MMU-17131	2.503	0.08018	shared	2.97	0	shared
Glycine degradatio	R-MMU-67839	2.588	0.00453	shared	1.961	0.09861	shared
Beta oxidation of n	R-MMU-77285	2.595	0	shared	1.546	0	shared
TET1,2,3 and TDG c	R-MMU-52210	2.595	0.00453	shared	2.305	0.05006	shared
