# Supplementary material for: Small Intestinal Tuft Cell Activity Associates With Energy Metabolism in Diet-Induced Obesity
Source: Front Immunol. 2021 May 28;12:629391. doi: 10.3389/fimmu.2021.629391 (PMC8195285; doi:10.3389/fimmu.2021.629391)
Supplement: Supplementary file 4 [file DataSheet_4.pdf]

Supplementary data 3: List of immune and/or metabolism related orphan genes in small intestinal tuft cells under HFD-feeding vs RFD-feeding conditions for 9 and 22 weeks.

GeneSymbol	GeneID	GeneName	HFD/RFD_fold change	*q-value*_ HFD/RFD	shared	PathwayID	PathwayName	Timepoint
Oasl2	ENSMUSG00000029561	2'-5' oligoaden	0.488	0	no			22 wks
A2ml1	ENSMUSG00000047228	alpha-2-macro	0.286	0.05154	no			9 wks
A430033K04Rik	ENSMUSG00000056014	RIKEN cDNA A4	0.175	0.09095	no			9 wks
Ablim3	ENSMUSG00000032735	actin binding L	0.093	0.07623	shared			9 wks
Ahnak2	ENSMUSG00000072812	AHNAK nucleo	0.337	0.05803	shared			9 wks
Akap11	ENSMUSG00000022016	A kinase (PRKA	0.399	0.09095	no			9 wks
Ankhd1	ENSMUSG00000024483	ankyrin repeat	0.297	0.07764	no			9 wks
Arid3a	ENSMUSG00000019564	AT rich interac	0	0.07764	no			9 wks
Arrdc4	ENSMUSG00000042659	arrestin domai	0.452	0.07764	no			9 wks
Atp5g1	ENSMUSG00000006057	ATP synthase,	2.036	0.09907	no			9 wks
Brat1	ENSMUSG00000000148	BRCA1-associa	0.266	0.05154	no			9 wks
Card10	ENSMUSG00000033170	caspase recruit	0.48	0.0329	no			9 wks
Cblc	ENSMUSG00000040525	Casitas B-linea	0.416	0.03228	no			9 wks
Cc2d1a	ENSMUSG00000036686	coiled-coil and	0.172	0.0865	no			9 wks
Ccpg1	ENSMUSG00000034563	cell cycle progr	0.444	0.05666	no			9 wks
Rpl5	ENSMUSG00000058558	ribosomal prot	1.934	0.01898	shared			22 wks
C1qtnf12	ENSMUSG00000023571	C1q and tumor	2.196	0.01898	no			22 wks
Atad1	ENSMUSG00000013662	ATPase family,	2.2	0.01898	no			22 wks
Hnrnpa1	ENSMUSG00000046434	heterogeneous	2.338	0.01898	shared			22 wks
Cct6a	ENSMUSG00000029447	chaperonin co	2.344	0.01017	no			9 wks
Nupr1l	ENSMUSG00000095789	nuclear protei	0.22	0.02064	no			22 wks
Zbp1	ENSMUSG00000027514	Z-DNA binding	0.355	0.02064	shared			22 wks
Mxd1	ENSMUSG00000001156	MAX dimerizat	0.382	0.02064	no			22 wks
Oas1g	ENSMUSG00000066861	2'-5' oligoaden	0.466	0.02064	no			22 wks
H2-T22	ENSMUSG00000056116	histocompatib	0.267	0.02231	shared			22 wks
Svbp	ENSMUSG00000028643	small vasohibi	0.548	0.02231	shared			22 wks
Cdk10	ENSMUSG00000033862	cyclin-depend	0.405	0.07058	no			9 wks
Cdkal1	ENSMUSG00000006191	CDK5 regulato	0.265	0.04816	no			9 wks
Cebpg	ENSMUSG00000056216	CCAAT/enhanc	0.49	0.04621	no			9 wks
Mprip	ENSMUSG00000005417	myosin phosp	0.41	0.02722	shared			22 wks
Ahnak2	ENSMUSG00000072812	AHNAK nucleo	0.568	0.02722	shared			22 wks
Mapk8ip3	ENSMUSG00000024163	mitogen-activa	0.408	0.03206	shared			22 wks
Chd7	ENSMUSG00000041235	chromodomai	0.414	0.07764	no			9 wks
Cic	ENSMUSG00000005442	capicua transc	0.459	0.0865	no			9 wks
Cir1	ENSMUSG00000041777	corepressor in	0.316	0.04621	no			9 wks
Clasrp	ENSMUSG00000061028	CLK4-associati	0.379	0.09095	no			9 wks
Cnst	ENSMUSG00000038949	consortin, con	0.397	0.07058	no			9 wks
Crtc1	ENSMUSG00000003575	CREB regulate	0.432	0.0865	no			9 wks
Ctcf	ENSMUSG00000005698	CCCTC-binding	0.278	0.04914	no			9 wks
Ctdsp2	ENSMUSG00000078429	CTD (carboxy-t	0.377	0.09095	no			9 wks
Ctdspl2	ENSMUSG00000033411	CTD (carboxy-t	0.426	0.05666	no			9 wks
Cwh43	ENSMUSG00000029154	cell wall bioge	0.205	0.05803	no			9 wks
Cyb561d1	ENSMUSG00000048796	cytochrome b-	0.351	0.07343	no			9 wks
Dcaf15	ENSMUSG00000037103	DDB1 and CUL	0.238	0.05803	shared			9 wks
Ddx60	ENSMUSG00000037921	DEAD (Asp-Glu	0.088	0.09642	shared			9 wks
Gbp6	ENSMUSG00000104713	guanylate bind	0.103	0.03822	no			22 wks
Med13l	ENSMUSG00000018076	mediator com	0.374	0.03822	no			22 wks
Oas2	ENSMUSG00000032690	2'-5' oligoaden	0.405	0.03822	shared			22 wks
Dedd	ENSMUSG00000013973	death effector	0.306	0.04029	no			9 wks
Dhx58	ENSMUSG00000017830	DEXH (Asp-Glu	0.353	0.04816	no			9 wks
Dhx8	ENSMUSG00000034931	DEAH (Asp-Glu	0.495	0.09095	no			9 wks
Dlg5	ENSMUSG00000021782	discs large MA	0.055	0.04816	no			9 wks
Dnajb12	ENSMUSG00000020109	DnaJ heat shoc	0.328	0.05154	no			9 wks
Dpp9	ENSMUSG00000001229	dipeptidylpept	0.362	0.07372	no			9 wks
Eif4a1	ENSMUSG00000059796	eukaryotic tra	2.518	0	no			9 wks
Eif4enif1	ENSMUSG00000020454	eukaryotic tra	0.417	0.05803	no			9 wks
Emsy	ENSMUSG00000035401	EMSY, BRCA2-i	0.399	0.09299	no			9 wks
Extl3	ENSMUSG00000021978	exostoses (mu	0.327	0.04029	no			9 wks
Fbxo33	ENSMUSG00000035329	F-box protein 3	0.456	0.09095	no			9 wks
Fkrp	ENSMUSG00000048920	fukutin related	0.436	0.07764	no			9 wks
Foxj2	ENSMUSG00000003154	forkhead box J	0.158	0.07058	no			9 wks
Fxr2	ENSMUSG00000018765	fragile X menta	0.16	0.0865	no			9 wks
Ganc	ENSMUSG00000062646	glucosidase, al	0.127	0.03228	no			9 wks
Gbp7	ENSMUSG00000040253	guanylate bind	0.074	0.05803	no			9 wks
Gm45140	ENSMUSG00000107928	predicted gene	0	0.0865	no			9 wks
Gsap	ENSMUSG00000039934	gamma-secreta	0	0.07504	no			9 wks
H2-T22	ENSMUSG00000056116	histocompatib	0.36	0.0865	shared			9 wks
Hdac5	ENSMUSG00000008855	histone deacet	0.447	0.07058	no			9 wks
Hivep3	ENSMUSG00000028634	human immun	0.425	0.04621	no			9 wks

Hmbox1	ENSMUSG00000021972	homeobox cor	0.362	0.04029	no	9 wks
Hmces	ENSMUSG00000030060	5-hydroxymetl	0.484	0.0865	no	9 wks
Hmgb1	ENSMUSG00000066551	high mobility g	2.356	0	no	9 wks
Hnrnpa1	ENSMUSG00000046434	heterogeneous	1.953	0.07721	shared	9 wks
Hspd1	ENSMUSG00000025980	heat shock pro	2.736	0.02495	no	9 wks
Htt	ENSMUSG00000029104	huntingtin	0.497	0.04816	no	9 wks
Igf2bp2	ENSMUSG00000033581	insulin-like gro	0.463	0.07058	no	9 wks
Ing1	ENSMUSG00000045969	inhibitor of gro	0.487	0.03865	no	9 wks
Jkamp	ENSMUSG00000005078	JNK1/MAPK8-a	0.499	0.0865	no	9 wks
Khdc4	ENSMUSG00000028060	KH domain cor	0.339	0.03865	no	9 wks
Ddx60	ENSMUSG00000037921	DEAD (Asp-Glu	0.196	0.04691	shared	22 wks
Naip5	ENSMUSG00000071203	NLR family, apo	0.465	0.04691	shared	22 wks
Lars2	ENSMUSG00000035202	leucyl-tRNA sy	0.301	0.03573	no	9 wks
LTO1	ENSMUSG00000031072	ABCE maturati	0.471	0.05666	no	9 wks
Macrod2	ENSMUSG00000068205	MACRO domai	0.256	0.0719	no	9 wks
Mapk8ip3	ENSMUSG00000024163	mitogen-activa	0.325	0.07058	shared	9 wks
March2	ENSMUSG00000079557	membrane-ass	0.427	0.07058	no	9 wks
March5	ENSMUSG00000023307	membrane-ass	0.484	0.0865	no	9 wks
Marf1	ENSMUSG00000060657	meiosis regula	0.486	0.04029	no	9 wks
Marveld3	ENSMUSG00000001672	MARVEL (mem	0.405	0.04621	no	9 wks
Mcts2	ENSMUSG00000042814	malignant T ce	0.448	0.0865	no	9 wks
Mn1	ENSMUSG00000070576	meningioma 1	0.305	0.04621	no	9 wks
Mprip	ENSMUSG00000005417	myosin phospl	0.191	0.04914	shared	9 wks
Mxd4	ENSMUSG00000037235	Max dimerizati	0.173	0.09095	no	9 wks
Myrf	ENSMUSG00000036098	myelin regulat	0.334	0.03865	no	9 wks
Naa15	ENSMUSG00000063273	N(alpha)-acety	0.464	0.05666	no	9 wks
Naa80	ENSMUSG00000079334	N(alpha)-acety	0.37	0.07343	no	9 wks
Npm1	ENSMUSG00000057113	nucleophosmi	2.133	0.0564	shared	22 wks
Naip5	ENSMUSG00000071203	NLR family, apo	0.319	0.04029	shared	9 wks
Neo1	ENSMUSG00000032340	neogenin	0.241	0.04029	no	9 wks
Neurl4	ENSMUSG00000047284	neuralized E3 u	0.09	0.07058	no	9 wks
Ngrn	ENSMUSG00000047084	neugrin, neurit	0.259	0.03228	no	9 wks
Npm1	ENSMUSG00000057113	nucleophosmi	2.124	0.01469	shared	9 wks
Oas2	ENSMUSG00000032690	2'-5' oligoaden	0.214	0.03573	shared	9 wks
Onecut2	ENSMUSG00000045991	one cut domai	0.463	0.07764	no	9 wks
Parp12	ENSMUSG00000038507	poly (ADP-ribo	0.397	0.09095	no	9 wks
Paxbp1	ENSMUSG00000022974	PAX3 and PAX7	0.421	0.09299	no	9 wks
Pcsk4	ENSMUSG00000020131	proprotein cor	0	0.04029	no	9 wks
Per3	ENSMUSG00000028957	period circadia	0.47	0.02462	no	9 wks
Plagl2	ENSMUSG00000051413	pleiomorphic a	0.24	0.09542	no	9 wks
Pmel	ENSMUSG00000025359	premelanosom	0.384	0.07343	no	9 wks
Polg	ENSMUSG00000039176	polymerase (D	0.345	0.09095	no	9 wks
Ppargc1a	ENSMUSG00000029167	peroxisome pr	0	0.03228	no	9 wks
Ppia	ENSMUSG00000071866	peptidylprolyl	2.761	0.01951	no	9 wks
Ppp1r9b	ENSMUSG00000038976	protein phospl	0.108	0.05803	no	9 wks
Csdc2	ENSMUSG00000042109	cold shock dor	0	0.06843	no	22 wks
Ppp2r2d	ENSMUSG00000041769	protein phospl	0.316	0.07764	no	9 wks
Prdm4	ENSMUSG00000035529	PR domain cor	0	0.04816	no	9 wks
Prkrip1	ENSMUSG00000039737	Prkr interactin	0.452	0.05803	no	9 wks
Prpf39	ENSMUSG00000035597	pre-mRNA pro	0.254	0.07058	no	9 wks
Ptms	ENSMUSG00000030122	parathymosin	0.401	0.04621	no	9 wks
Ptpn23	ENSMUSG00000036057	protein tyrosin	0.169	0.07764	no	9 wks
Ptpre	ENSMUSG00000041836	protein tyrosin	0.291	0.04029	shared	9 wks
Pzp	ENSMUSG00000030359	PZP, alpha-2-m	0.031	0.05803	no	9 wks
Qrsl1	ENSMUSG00000019863	glutaminyl-tRN	0.471	0.04029	no	9 wks
Qtrt1	ENSMUSG00000002825	queuine tRNA-	0.456	0.09095	no	9 wks
Rab11fip5	ENSMUSG00000051343	RAB11 family i	0.098	0.09095	no	9 wks
Rbm4b	ENSMUSG00000033760	RNA binding m	0.321	0.03228	no	9 wks
Rbpms	ENSMUSG00000031586	RNA binding pi	0.207	0.09881	no	9 wks
Rc3h2	ENSMUSG00000075376	ring finger and	0.466	0.03228	no	9 wks
Repin1	ENSMUSG00000052751	replication init	0.306	0.0865	no	9 wks
Rhbdf2	ENSMUSG00000020806	rhomboid 5 ho	0.32	0.0865	no	9 wks
Rmnd5b	ENSMUSG00000001054	required for m	0.468	0.07764	no	9 wks
Rnasek	ENSMUSG00000093989	ribonuclease, I	0.43	0.05803	no	9 wks
Rpl17	ENSMUSG00000062328	ribosomal prot	2.054	0	no	9 wks
Rpl23a	ENSMUSG00000058546	ribosomal prot	2.188	0.01017	no	9 wks
Rpl27	ENSMUSG00000063316	ribosomal prot	2.073	0.01017	no	9 wks
Rpl5	ENSMUSG00000058558	ribosomal prot	2.26	0.0326	shared	9 wks
Ptpre	ENSMUSG00000041836	protein tyrosin	0.379	0.0768	shared	22 wks
Dcaf15	ENSMUSG00000037103	DDB1 and CUL	0.446	0.0768	shared	22 wks
Rpl6	ENSMUSG00000029614	ribosomal prot	2.088	0	no	9 wks
Rpl7	ENSMUSG00000043716	ribosomal prot	2.002	0	no	9 wks
Rpl7a	ENSMUSG00000062647	ribosomal prot	2.042	0	no	9 wks

Rps27a	ENSMUSG00000020460	ribosomal prot	2.254	0	no	9 wks
Rps6	ENSMUSG00000028495	ribosomal prot	2.808	0	no	9 wks
Rsf1	ENSMUSG00000035623	remodeling an	0.399	0.03865	no	9 wks
Rubcn	ENSMUSG00000035629	RUN domain a	0.376	0.05666	no	9 wks
Rusc1	ENSMUSG00000041263	RUN and SH3 c	0.317	0.07764	shared	9 wks
Sap30bp	ENSMUSG00000020755	SAP30 binding	0.32	0.03228	no	9 wks
Sbno2	ENSMUSG00000035673	strawberry not	0.389	0.05803	no	9 wks
Serpini1	ENSMUSG00000027834	serine (or cyste	2.372	0	no	9 wks
Sik3	ENSMUSG00000034135	SIK family kina	0.409	0.07504	no	9 wks
Gal3st2	ENSMUSG00000094651	galactose-3-O-	0.419	0.07845	no	22 wks
Tgtp1	ENSMUSG00000078922	T cell specific G	0	0.07889	no	22 wks
Ssbp4	ENSMUSG00000070003	single strandec	0.414	0.08154	no	22 wks
Tnk1	ENSMUSG00000001583	tyrosine kinase	0.544	0.08154	shared	22 wks
Sirt2	ENSMUSG00000015149	sirtuin 2	0.456	0.05154	no	9 wks
Sirt5	ENSMUSG00000054021	sirtuin 5	0.371	0.09299	no	9 wks
Slf2	ENSMUSG00000036097	SMC5-SMC6 cc	0.468	0.04029	no	9 wks
Smcr8	ENSMUSG00000049323	Smith-Magenis	0.137	0.04029	no	9 wks
Smg1	ENSMUSG00000030655	SMG1 homolog	0.274	0.04029	no	9 wks
Smg8	ENSMUSG00000020495	smg-8 homolog	0.223	0.07058	no	9 wks
Smu1	ENSMUSG00000028409	smu-1 suppres	2.369	0.03526	no	9 wks
Snrk	ENSMUSG00000038145	SNF related kir	0.238	0.03228	no	9 wks
Snx33	ENSMUSG00000032733	sorting nexin 3	0	0.0865	no	9 wks
Socs7	ENSMUSG00000038485	suppressor of c	0.183	0.04029	no	9 wks
Srcap	ENSMUSG00000053877	Snf2-related CI	0.417	0.07264	no	9 wks
Srpk2	ENSMUSG00000062604	serine/arginine	0.273	0.09095	no	9 wks
Ssb	ENSMUSG00000068882	Sjogren syndrc	2.303	0.01017	no	9 wks
Stk38l	ENSMUSG00000001630	serine/threoni	3.308	0.02495	no	9 wks
Svbp	ENSMUSG00000028643	small vasohibir	0.444	0.03865	shared	9 wks
Swt1	ENSMUSG00000052748	SWT1 RNA enc	0.352	0.09095	no	9 wks
Szt2	ENSMUSG00000033253	seizure thresho	0.322	0.05154	no	9 wks
Taok2	ENSMUSG00000059981	TAO kinase 2	0.3	0.0865	no	9 wks
Tbc1d5	ENSMUSG00000023923	TBC1 domain f	0.361	0.09095	no	9 wks
Tmem38b	ENSMUSG00000028420	transmembran	0.324	0.03865	no	9 wks
Tnk1	ENSMUSG00000001583	tyrosine kinase	0.479	0.0865	shared	9 wks
Ifit1bl1	ENSMUSG00000079339	interferon indu	0.218	0.08802	no	22 wks
Tob2	ENSMUSG00000048546	transducer of E	0.465	0.04914	no	9 wks
Trim24	ENSMUSG00000029833	tripartite motif	0.389	0.03865	no	9 wks
Trim26	ENSMUSG00000024457	tripartite motif	0.427	0.0865	no	9 wks
Tshz1	ENSMUSG00000046982	teashirt zinc fir	0.248	0.04621	no	9 wks
Tspan17	ENSMUSG00000025875	tetraspanin 17	0.222	0.0865	no	9 wks
Ubr5	ENSMUSG00000037487	ubiquitin prote	0.43	0.0865	no	9 wks
Ulk2	ENSMUSG00000004798	unc-51 like kin	0.298	0.05154	no	9 wks
Unc5cl	ENSMUSG00000043592	unc-5 family C-	0.467	0.0865	no	9 wks
Uox	ENSMUSG00000028186	urate oxidase	0.269	0.04029	no	9 wks
Usp49	ENSMUSG00000090115	ubiquitin speci	0.393	0.04621	no	9 wks
Vps16	ENSMUSG00000027411	VSP16 CORVET	0.441	0.05666	no	9 wks
Zbp1	ENSMUSG00000027514	Z-DNA binding	0.436	0.07058	shared	9 wks
Zbtb7b	ENSMUSG00000028042	zinc finger and	0.361	0.0865	no	9 wks
Zc3h12a	ENSMUSG00000042677	zinc finger CCC	0.203	0.04816	no	9 wks
Zdhhc7	ENSMUSG00000031823	zinc finger, DH	0.337	0.07764	no	9 wks
Zfhx2	ENSMUSG00000040721	zinc finger hon	0.342	0.07058	no	9 wks
Zfp239	ENSMUSG00000042097	zinc finger prot	0.46	0.09095	no	9 wks
Zfp318	ENSMUSG00000015597	zinc finger prot	0.091	0.09095	no	9 wks
Zfp358	ENSMUSG00000047264	zinc finger prot	0	0.09661	no	9 wks
Zfp36	ENSMUSG00000044786	zinc finger prot	0.314	0.05666	no	9 wks
Zfp444	ENSMUSG00000044876	zinc finger prot	0.357	0.0865	no	9 wks
Zfp609	ENSMUSG00000040524	zinc finger prot	0.093	0.04029	no	9 wks
Zfp821	ENSMUSG00000031728	zinc finger prot	0	0.09095	no	9 wks
Zmiz1	ENSMUSG00000007817	zinc finger, MIZ	0.443	0.04621	no	9 wks
Zmynd11	ENSMUSG00000021156	zinc finger, MY	0.486	0.07058	no	9 wks
